# Ring-Opening Alkyne Metathesis Polymerization Catalyzed by a Bench-Stable Rhenium Complex

**DOI:** 10.1021/jacs.5c19886

**Published:** 2026-01-16

**Authors:** Yinuo Zheng, Ruby L. Y. Chan, Somin Cha, Gregory I. Peterson, Jie Huang, Guochen Jia, Pauline Chiu, Antonio Rizzo

**Affiliations:** † Department of Chemistry and State Key Laboratory of Synthetic Chemistry, 25809 The University of Hong Kong, Pokfulam Road, Kowloon, Hong Kong 999077, P. R. China; ‡ Department of Chemistry and Research Institute of Basic Science, 34958 Incheon National University, Incheon 22012, Republic of Korea; § Department of Chemistry, 58207 The Hong Kong University of Science and Technology, Clear Water Bay, Kowloon, Hong Kong 999077, P. R. China

## Abstract

In the last quarter of a century, ring-opening alkene metathesis polymerization has revolutionized the ability to synthesize functional polymers. In comparison, current methods for ring-opening alkyne metathesis polymerization (ROAMP) remain constrained by a narrow scope of monomers and catalysts that are not user-friendly. Herein, we report that an air-stable d^2^ Re­(V) alkylidyne precatalyst complex mediates ROAMP in a controlled fashion, even tolerating free hydroxyl groups in the monomer. This polymerization exhibits excellent control, with up to 700 degrees of polymerization of nonyne monomers with a low to moderate molecular weight dispersity. The metal can be cleaved from the polymer chain by means of cross-metathesis with commercially available phenylacetylenes. Additionally, this polymerization shows living character, which enables the preparation of block copolymers. These results highlight the potential of Re­(V)-mediated polymerizations to enable a broader use of ROAMP for the synthesis of functional macromolecules.

## Graphical abstract

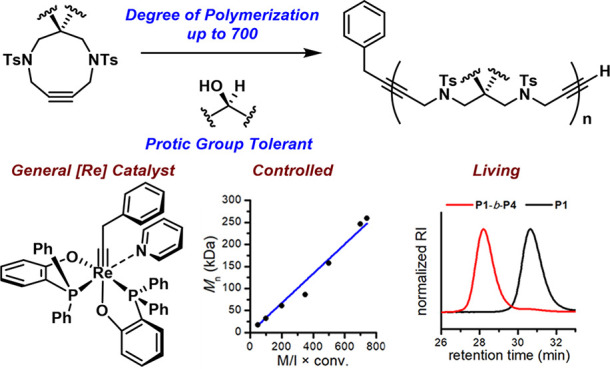

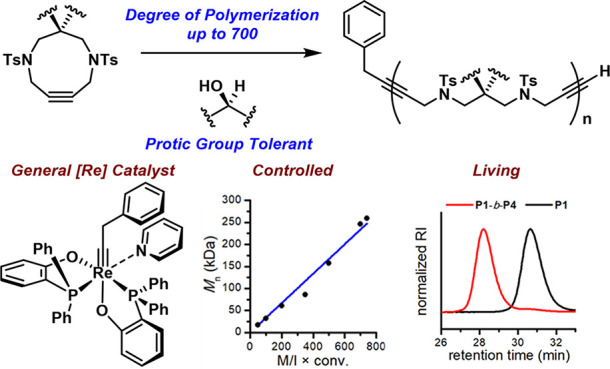

## Introduction

Olefin ring-opening metathesis polymerization (ROMP) has become one of the benchmark methods to prepare various polymeric materials, including degradable/depolymerizable polymers,^1^ biorelated materials,^2^ polymers for imaging,^3^ and biomedicine (Figure 1a). The polymer community has widely adopted ROMP, thanks in large part to the ruthenium complexes developed by late R. H. Grubbs, which exhibit excellent functional group tolerance and convenience of handling and use. On the other hand, ring-opening alkyne metathesis polymerization (ROAMP) has not followed the same trajectory for the lack of these features. Therefore, it would be desirable to address these shortcomings of ROAMP, as has been done with ROMP.

**Figure 1:**
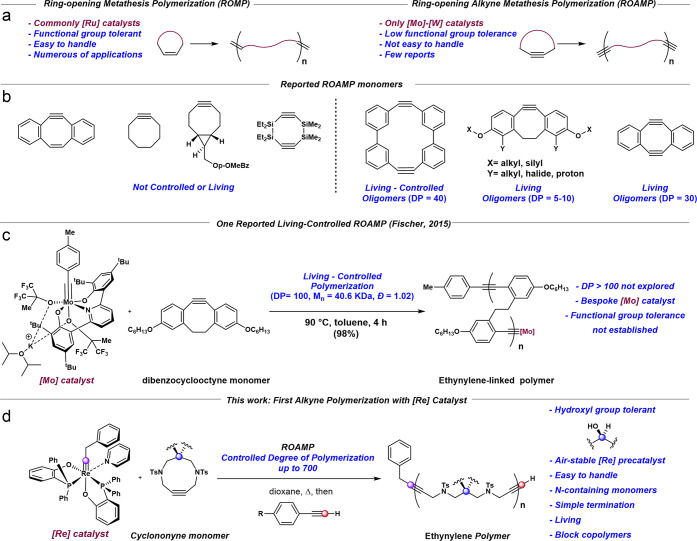
(a) General features of olefin ring-opening metathesis polymerization (ROMP) and ring-opening alkyne metathesis polymerization (ROAMP). (b) Comprehensive list of the monomers used in ROAMP. (c) The sole reported example of controlled, living ROAMP. (d) This work: the first ROAMP catalyzed by a d^2^ Re­(V) alkylidyne catalyst.

The past two decades have seen an acceleration of the development of alkyne metathesis from being a specialized technology carried out only by experts to a method that can be more widely applied to make small molecules and materials.^4^ Efforts that contribute to this evolution have been reported by the groups of Fürstner,^5^ Zhang,^6^ Tamm,^7^ and others^8^ who have tamed the high reactivity of d^0^ molybdenum and tungsten alkylidyne complexes for alkyne metathesis by means of ligand design. These catalysts are now reasonably stable to air and moisture while retaining good reactivity and functional group tolerance. Nevertheless, alkyne metathesis polymerization has seen fewer developments in terms of catalyst design and the breadth of application. Between acyclic diyne metathesis polymerization (ADIMET) and ROAMP, the former has seen wider utilization. Catalyzed by Mo or W alkylidyne complexes, ADIMET has generated ethynylene-linked materials used for ion-transport and light-harvesting,^4a,4b^ conjugated organic polymers,^7^ and rigid 3-D covalent organic polyhedrons.^4c,9^ A comprehensive survey of ROAMP literature, in comparison, reveals a rather limited number of reports. One clear constraint is the number and type of reported monomers, which do not offer many options for diversification and are poorly stable while requiring multistep preparations. These can be divided into two groups (Figure 1b), the first of which was polymerized mainly by [W] alkylidynes in a noncontrolled and nonliving fashion, starting with the seminal report by Krouse and coworkers in 1987.^10^ The second monomer group is mainly composed of compounds whose polymerization is catalyzed by more structurally defined [Mo] catalysts. These polymerizations were found to be living, and in one case controlled, but delivering only oligomeric compounds with a degree of polymerization (DP) less than 50.^4d,10f,11^ Recently, one of these monomers has been used in the only reported ring-expansion alkyne metathesis polymerization (REMP) to date, although not by a living or controlled process.^12^ The exception in this group is represented by the work of Bellone and coworkers, reported in 2015,^13^ that described the preparation of a custom molybdenum ONO pincer complex (Figure 1c) which polymerized dibenzocyclooctyne monomers to deliver the corresponding ethynylene-linked polymers with excellent molecular weight dispersity (*Đ* = 1.02) and up to DP = 100.

These reports indicate substantial opportunities for further exploration and advancement of ROAMP, particularly in the areas of monomer and catalyst design. The current ROAMP catalysts are not easily handled or prepared, and as a result, the community has not extensively used them. In this regard, non-d^0^ metal carbyne complexes and their reactivity have been much less studied and developed.^14^ Only in 2020 did Jia and coworkers unveil the first series of well-defined d^2^ Re­(V) alkylidyne complexes which are competent in catalytic homo- and cross-alkyne metathesis (**[Re]**, Figure 1d).^15^ These complexes show remarkable air and moisture stability as well as excellent functional group tolerance. Indeed, they have been prepared on multigram scales, stored and transported without stringent requirements, and are stable over time. Similar to the trends observed for olefin metathesis,^16^ the Re­(V) late transition metal complex appears to be more stable toward polar groups than [W] or [Mo]. In terms of ease of handling, their manipulation is similar to Grubbs-type catalysts,^17^ and therefore, these Re­(V) catalysts are ideal to employ for polymerization.^18^ Development of this chemistry would provide an orthogonal tool to the current Mo/W systems and expand the underdeveloped ROAMP technology. The d^2^ Re­(V) alkylidynes need to undergo an energetically demanding pyridine dissociation to convert to the catalytically active species, the calculated transition state Δ*G*‡ being ca. 27 kcal/mol at 100 °C.^19^ Under these conditions, the selection and design of monomers could include those that are less-strained than those previously used (Figure 1b). This led us to select *N*-sulfonamide cyclononynes, alkynes previously used for bioconjugation, as monomers.^20^ The cyclononynes are generally crystalline solids that are easily prepared from readily available starting materials on gram scales and readily purified. Moreover, an abundance of analogues are known,^21^ which offer handles for derivatization^22^ or postpolymerization modifications, if required. For example, *N*-4-nitrobenzenesulfonyl cyclononynes^20d^ feature easily deprotectable sites for postpolymerization modification, which would generate *N*–H-rich polymers.

Herein, we report the first rhenium-catalyzed ROAMP of *N*-sulfonamide cyclononyne monomers. The polymerization was found to be controlled with DPs up to 700. Moreover, hydroxylated monomers were also polymerized in a controlled fashion (up to DP 350), underscoring the functional group tolerance of the system. Additionally, our study of the termination shows that commercially available phenyl alkynes can selectively transfer the terminal methylidyne carbon. Finally, the living characteristics of the reaction were demonstrated by the synthesis of amphiphilic block copolymers.

## Results and Discussion

Our first goal was to identify optimal conditions for the **[Re]** catalyst to polymerize monomer **M1** to polymer **P1≡Re** (Figure 2a). Among the reported complexes, **[Re]** was selected, as it featured a good kinetic profile and undergoes rapid pyridine dissociation; it shows excellent bench stability as well as good stability in solution over extended reaction times. Finally, it is readily available as it is synthesized from a commercially available ligand.

**Figure 2:**
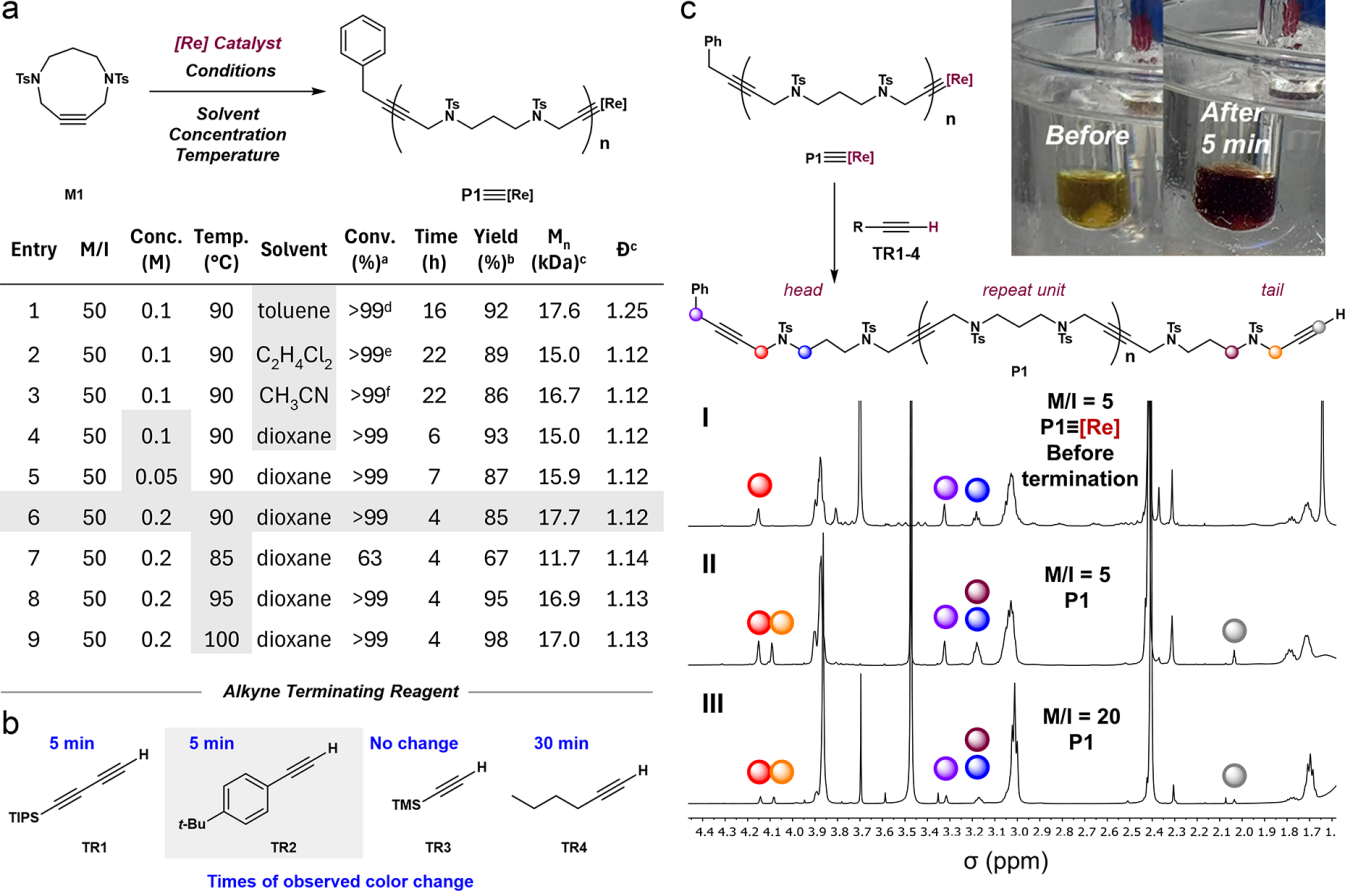
(a) Initial screening for the polymerization of **M1**. Polymerizations were conducted in a degassed solvent. ^a^Determined by ^1^H NMR analysis of the crude mixture. ^b^Isolated yield. ^c^Determined by THF size exclusion chromatography calibrated by polystyrene standards. ^d^86% conversion after 6 h; precipitates observed. ^e^73% conversion after 6 h. ^f^39% conversion after 6 h, precipitates observed. (b) Terminating reagents **TR1–4** with times of observed color change. (c) ^1^H NMR study of the termination of **P1**: stacked ^1^H NMR (400 MHz, CDCl_3_) of (I) crude **P1** (M/I = 5) before addition of terminating reagent, (II) crude **P1** (M/I = 5) after **TR2** addition (5 drops), and (III) crude **P1** (M/I = 20) after **TR2** addition (5 drops).

The initial screening was carried out without termination. We used conditions described by Jia for the homodimerization as the starting point for these optimizations,^15a,15b^ conducting the reaction by heating in toluene that had been degassed by sparging with argon for 15 min (Entry 1, table in Figure 2a).

Monomer **M1** was polymerized with **[Re]** alkylidyne in toluene (ca. 40 mg, monomer-to-initiator ratio (M/I) = 50, 0.1 M in toluene, 90 °C). The monomer was not soluble at room temperature, but upon heating, it dissolved completely to give a homogeneous, bright yellow solution. Through ^1^H NMR analysis of aliquots, the polymerization was found to have reached 86% conversion after 6 h and full conversion in 16 h. Precipitation from methanol gave **P1** as an off-white polymer in 92% yield with a number-average molecular weight (*M*_n_) of 17.6 kDa and a *Đ* of 1.25. This result confirmed the feasibility of the polymerization, but we surmised that the use of a more polar solvent to keep the macromolecule in solution throughout the process should be beneficial. Therefore, dichloroethane, acetonitrile, and dioxane were evaluated as solvents (Entries 2–4). All of these resulted in similar *M*_n_ and *Đ* (*M*_n_ = 15.0–16.7 kDa, *Đ* = 1.12), but the reaction in dioxane was faster. It was the only solvent in which the reaction was complete within 6 h and occurred without obvious precipitation. Moreover, the higher boiling point of dioxane provided a wider range to increase the reaction temperature. Reaction concentrations as low as 0.05 M and as high as 0.2 M were also evaluated (Entries 5–6). The *M*_n_ increased slightly with the higher concentration, while the dispersity was unchanged (*Đ* = 1.12). We selected 0.2 M as the concentration to continue with our experiments as this brought the reaction completion time down to 4 h (Entry 6, 99% conversion). Finally, we tested temperatures ranging from 85 to 100 °C (Entries 7–9). The reaction at 85 °C was incomplete after 4 h (63% conversion) with *M*_n_ = 11.7 kDa and *Đ* = 1.14 (Entry 7), whereas the results of reactions at higher temperatures were not significantly different from those at 90 °C (Entries 6, 8–9). Therefore, we selected the conditions in Entry 6 (0.2 M dioxane, 90 °C) as optimal, with the understanding that slightly increased reaction temperatures would not be particularly deleterious.

To develop a termination protocol, we started by analyzing the ^1^H and ^31^P NMR spectra of **P1** after precipitation and observed that the ligands on the metal dissociated only in part (See Supporting Information, Section 5.2). This, taken together with the persistence of the yellow color during the reaction (Figure 2c, left) and the symmetrical shape of the GPC peaks without significant tailing (See Supporting Information, Section 3.1.5), indicated that the chain-end might still be reactive at the end of the polymerization, so that it might be possible to add a quencher. There have only been two reported terminations of molybdenum ROAMP with alkynes, which successfully dealt with the regioselectivity problem by quenching with polarized ynamides.^4d,11b^ In the case of rhenium complexes related to [Re], it was known that terminal aryl alkynes reacting with a [Re≡CH_2_CPh] complex transferred selectively the methylidyne carbon to form [Re≡CPh] and HC≡CH_2_CPh, a process that is thermodynamically and kinetically favored.^23^ Taking advantage of this, we evaluated several small, alkyne-terminating reagents (**TR1**, **2**, **3**, **4**, Figure 2b) in the polymerization of **M1** (M/I = 5, 0.2 M in dioxane, 90 °C), by adding one alkyne to each of several aliquots after reaction for 1 h and then subjecting the samples to NMR analysis. In the cases of **TR1** and **TR2**, an apparent color change from yellow to brown was observed immediately upon addition. This signaled that the aryl alkyne had very likely transferred to form a [Re≡CPh] complex, as these species are known to be reddish in color.^15a^ No color change was observed with compound **TR3** while the use of **TR4** attained a similar brown tinge after ca. 30 min. A comparison of the ^1^H NMR spectra of crude **P1** before and after addition of **TR1** (**top** and **middle**, Figure 2c) revealed the presence of new signals consistent with a terminal alkyne. When the same process was repeated with an M/I = 20 polymerization (0.2 M in dioxane, 90 °C for 2 h, **bottom**, Figure 2), the same signals were present but were lower in intensity, consistent with the initial interpretation (See Supporting Information, Section 5.2, for more spectral assignments). Since **TR2** is commercially available, we selected it as the termination reagent of choice.

Next, we studied the range of M/I ratios at which the polymerization could reach full conversion for monomers **M1-M4**. We started with **M1**, and we polymerized it under the optimized conditions (0.1 mmol, 0.2 M dioxane, 90 °C), increasing the M/I ratio from 50 (Table 1, Entry 1), which was our optimization benchmark, to M/I = 100. Full conversion of **M1** was observed within 12 h (Entry 2). Upon precipitation, **P1** was isolated with a doubled *M*_n_ = 32.2 kDa and *Đ* = 1.19. We also tried a 1 mmol scale-up of the same polymerization. This reaction was also complete in ca. 12 h, producing **P1** with identical *M*_n_ and *Đ* (Entry 3). The kinetic plot revealed a pseudolinear conversion, which attenuates slightly above 60%. In fact, the mathematical treatment of these data shows first-order kinetics (ln­([M]/[M]_0_) vs time) in the first half of the reaction (See Supporting Information, Section 3.3) to reach full conversion at M/I = 200 required increasing the temperature to 95 °C (<70% conv. at 90 °C). This resulted in a similarly well-behaved polymerization to **P1** (Entry 4, *M*_n_ = 61.1 kDa, *Đ* = 1.21). Experiments with M/I = 350 and M/I = 500 also needed higher reaction temperatures of 100 and 105 °C, respectively, in order to reach full conversion (Entries 5 and 6). The *M*_n_ increased linearly to 86.2 and 157.1 kDa, respectively, with a corresponding increase in *Đ* to 1.67 and 1.57. Finally, polymerizations at M/I = 700 and 1000 were also attempted at 105 °C (Entries 7 and 8). The experiment with M/I = 700 attained full conversion successfully within 48 h, with a *M*_n_ = 246.3 kDa (*Đ* = 1.53), whereas the M/I = 1000 reaction reached 74% conversion in 48 h with *M*_n_ = 258.4 kDa (*Đ* = 1.94). Extending the reaction time beyond 48 h did not result in further conversion. Overall, the ROAMP of **M1** with the **[Re]** precatalyst induced remarkably linear increases in *M*_n_ up to M/I = 700 (Figure 3a). The dispersity remained consistently narrow up to M/I = 200, above which partial termination was evident from the SEC traces.

**Table 1:**
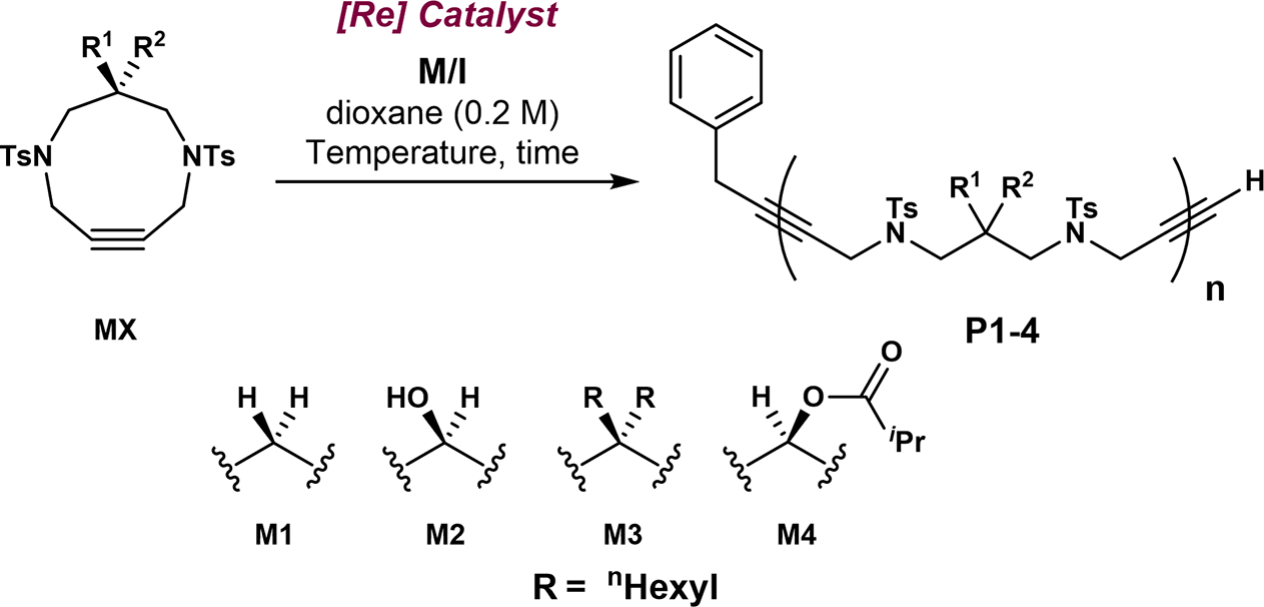
Polymerization of Monomers **M1**-**M4** ^a^

Entry	Monomer	M/I	Temp. (°C)	Conv. (%)^b^	Time (h)	Yield (%)^c^	*M*_n_ (kDa)^d^	*Đ* ^d^
1	M1	50	90	>99	4	81	16.8	1.12
2	M1	100	90	>99	12	96	32.2	1.19
3	M1	100^e^ ^,^ ^f^	90	>99	12	83	32.2	1.19
4	M1	200	95	>99	21	99	61.1	1.21
5	M1	350	100	>99	48	98	86.2	1.67
6	M1	500	105	>99	48	75	157.1	1.57
7	M1	700	105	>99	48	92	246.3	1.53
8	M1	1000	105	74	48	70	258.4	1.94
9	M2	20	90	>99	2	80	8.3	1.2
10	M2	50	90	>99	4	91	21.7	1.2
11	M2	75	90	>99	22	94	28.7	1.38
12	M2	100	90	92	17	89	35.5	1.32
13	M2	125	90	>99	22	92	43.3	1.68
14	M2	200	90	>99	21	89	61.8	1.82
15	M2	250	100	95	24	79	93.2	2.08
16	M2	350	105	95	46	93	118.7	2.08
17	M3	20	100	>99	20	48	8.7	1.13
18	M3	50	100	96	24	43	28.5	1.28
19	M3	75	105	79	43	53	50.6	1.57
20	M4	20	90	>99	2	71	9.1	1.19
21	M4	50	90	>99	4	76	21.3	1.15
22	M4	100	90	>99	17	69	40.3	1.17
23	M4	200	90	>99	17	79	78.3	1.39
24	M4	350	100	>99	23	73	151.2	1.34
25	M4	500	105	>99	23	93	235.2	1.3

a Polymerizations were conducted in degassed dioxane (0.2 M) at 0.2 mmol scale.

b Determined by ^1^H NMR analysis of the crude mixture.

c Isolated yield.

d Determined by THF size exclusion chromatography calibrated by polystyrene standards.

e Polymerization carried out at 1 mmol scale.

f Yield adjusted to account for the removed volume, see SI, Section 3.3 for details.

**Figure 3:**
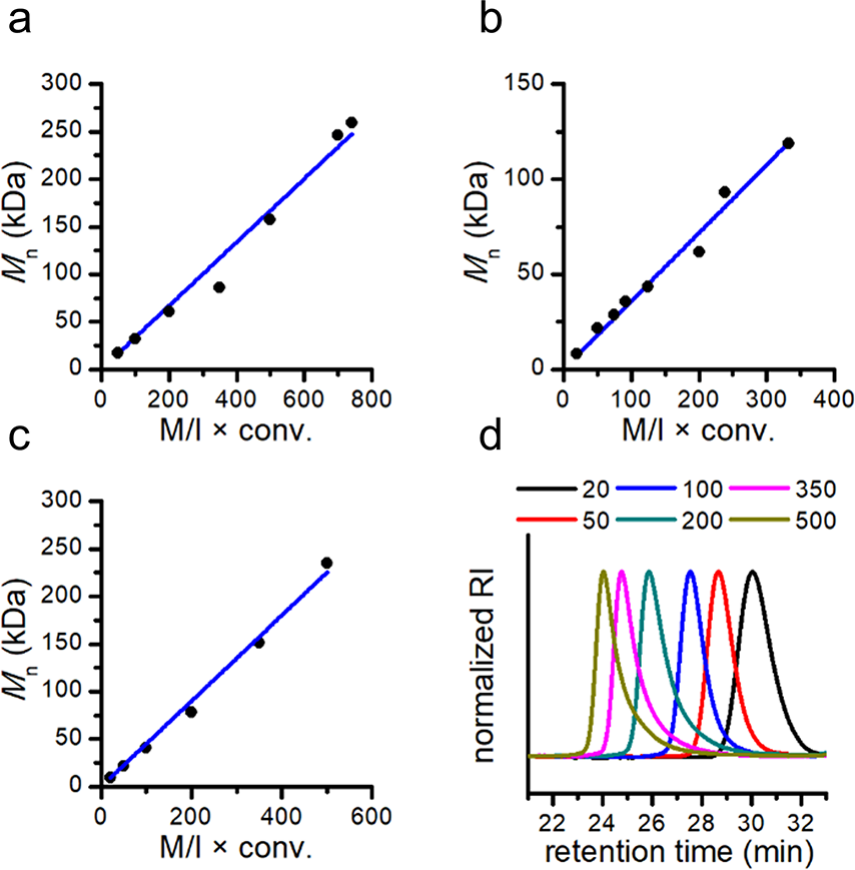
Linear plots of *M*_n_ vs M/I × conversion for (a) **P1**, (b) **P2**, and (c) **P4**. (d) SEC traces of **P4** which correspond to the entries in Table 1. Numbers in the legend indicate the targeted degree of polymerization. Adjusted *R* ^2^ values for (a)-(c) are 0.990, 0.993, and 0.996, respectively.

We then investigated the polymerization of **M2**, which is a monomer with a free hydroxyl group. Although it exhibits a substantially inferior solubility compared to **M1**, the polymerization of **M2** could probe the functional group tolerance of the catalytic system as no ROAMP has reported the polymerization of alcohol monomers to date. Therefore, **M2** was polymerized under the optimized conditions (0.2 M dioxane, 90 °C) at a M/I = 20 (Entry 9). Full consumption of **M2** was attained within 2 h, and we isolated polymer **P2** with *M*_n_ = 8.3 kDa and *Đ* = 1.20. Increasing the ratio to M/I = 50, 75, and 100 (Entries 10–12) produced a linear increase in *M*_n_ (Figure 3b). We found **P2** to be a polymer that aggregates as clumps, instead of the powder form that is **P1**. Further increasing the ratio to M/I = 125, 200, 250, and 350 led to a noticeable increase in the viscosity of the reaction mixture, but all experiments showed conversions that were >90% (Entries 13–16). The *M*_n_ increased linearly, but the dispersity was clearly more broad (*Đ* = 1.68–2.08) due to chain termination (See Supporting Information for **P2** SEC traces, Section 3.1.5).

To further probe the effects of the polymerization induced by substituents on the monomer, we tested **M3**, which bears geminal ^n^hexyl groups. However, the disubstitution of **M3** led to considerably decreased reaction rates, as **M3** at M/I = 20 needed heating at 100 °C for over 20 h to reach full conversion to **P3** (Entry 17, *M*_n_ 8.7 kDa (*Đ* = 1.13)), compared to **M2**, which only needed 4 h at 90 °C (Entry 9). At M/I = 50, the polymerization reached 96% conversion (Entry 18), while at M/I = 75, only a 79% conversion was achieved after 43 h at 105 °C (Entry 19). At higher M/I ratios, the polymerization achieved only moderate conversions of >50%. Presently, we surmise that a conformational difference in the ring of **M3** compared to **M1** led to the observed unsatisfactory reactivity (See Supporting Information, Section 8, Figure S27).

Monomer **M4**, which is obtained from the acylation of **M2**, was polymerized under the standard conditions. We observed that experiments with M/I = 20, 50, and 100 all resulted in full conversions to produce **P4** with narrow dispersities and growing *M*_n_ (Entries 20–22, 9.1–40.3 kDa, *Đ* = 1.19–1.17). Notably, polymerization at an M/I ratio of 200 also achieved complete monomer conversion, accompanied by a consistent increase in *M*_n_ and a slightly broader *Đ* (Entry 23, *M*_n_ = 78.3 kDa, *Đ* = 1.39). Experiments with M/I ratios of 350 and 500 required raising the reaction temperature to 100 and 105 °C, respectively, in order for the polymerizations to attain full conversion (Entries 24–25). The *M*_n_ grew to 151.2 and 235.2 kDa, respectively, while the dispersity remained relatively narrow. Overall, **M4** was polymerized in a controlled manner with a linear increase in *M*_n_ similar to that of **M1**, but with a narrower *Đ* at up to M/I = 500. Overall, this indicates that, in contrast to geminal substitution, monosubstitution on the monomer linker is not problematic (Figure 3c and d). **P4** also presented the possibility of postpolymerization modification, to undergo hydrolysis under mild conditions to generate **P2** (See Supporting Information, Section 7). Finally, although we could not directly compare the measured *M*_n_ values with the theoretical *M*_n_ (the *M*_n_ values were determined relative to a polystyrene standard), we used the ^1^H NMR spectra of the M/I = 20 polymers to determine the degree of polymerization (See Supporting Information, Section 8). For monomers **M1-M4**, the DP was in good agreement with the intended M/I (19–24 range), notwithstanding the limits of accuracy of ^1^H NMR integration being at 95:5.

Taking advantage of the good control and livingness of the **[Re]**-catalyzed ROAMP, we synthesized a block copolymer from **P1** and **P2** (Figure 4a). After preparing **P2** (**M2**, M/I = 20, dioxane 0.2 M, 90 °C, 2 h) with a *M*_n_ of 8.3 kDa (*Đ* = 1.20), the addition of a solution of 50 equiv of **M1** in a 1:1 mixture of DCE:dioxane yielded **P2-** *b* **-P1** with a *M*_n_ of 29.3 kDa and *Đ* of 1.20. A clear shift to the higher molecular weight region in the SEC trace confirmed that block copolymerization was successful (Figure 4c), providing further confirmation of the living nature of the polymerization. The ^1^H NMR data of the block also agreed with the complete incorporation of both monomers, as the ratio of **P2**:**P1** measured by integration was found to be 18.6:50 (See Supporting Information, Section 3.2).

**Figure 4:**
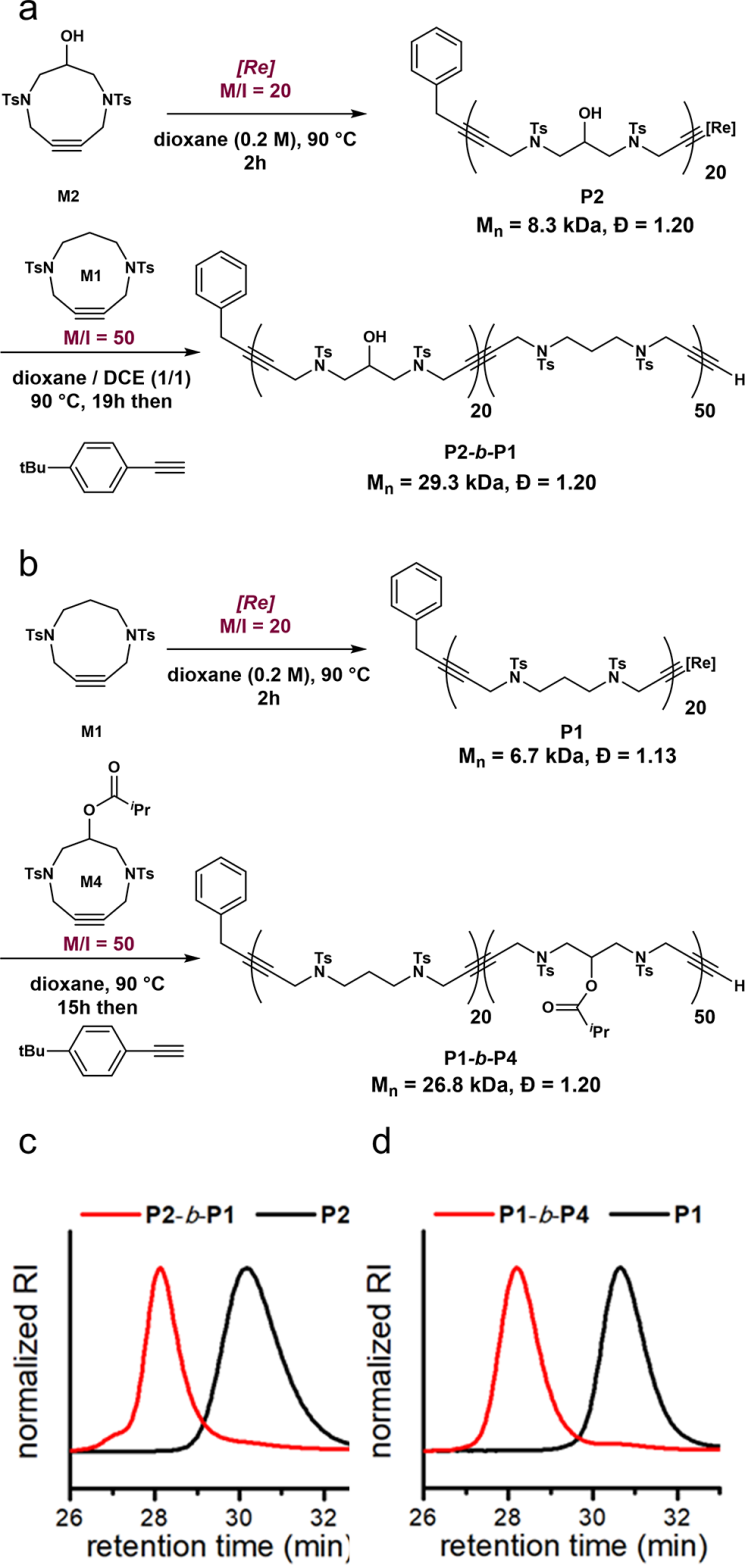
(a) Synthesis of block copolymer, **P2-** *b* **-P1**, and (c) SEC traces for **P2** and **P2-** *b* **-P1**. (b) Synthesis of block copolymer, **P1-** *b* **-P4**, and (d) SEC traces for **P1** and **P1-** *b* **-P4**.

In a separate experiment, we polymerized **P1** (**M1**, M/I = 20, dioxane 0.2 M, 90 °C, 2 h) with a *M*_n_ of 6.7 kDa (*Đ* = 1.13) and then added 50 equiv of **M4** in dry, degassed dioxane. After 15 h, we isolated **P1-** *b* **-P4** with a *M*_n_ of 26.8 kDa and *Đ* of 1.20, where the SEC trace also confirmed successful block copolymerization (Figure 4d, ^1^H NMR ratio of **P1**:**P4** = 17.8:50, See Supporting Information, Section 3.2).

## Conclusions

In conclusion, we successfully applied a [Re] alkylidyne catalyst to polymerize heterocyclic nonyne monomers via a ROAMP mechanism. This is the first application of a well-defined d^2^ Re­(V) alkylidyne complex in ROAMP. We optimized the polymerization initiated by the [Re] complex and observed a controlled process with DPs up to 700. Four types of monomers with different solubilities were investigated, and they showed marked differences in polymerizability. It is worthy to note that this catalytic system can sustain the complete polymerization of hydroxyl-containing monomers in a controlled fashion up to M/I = 350, which demonstrates the unprecedented functional group tolerance of this ROAMP catalyst. In addition, we successfully developed a termination for this polymerization, which involves the addition of commercially available aryl acetylene. We observed that a derivatization of the monomers, as exemplified by the acetylation of **M2** to provide **M4**, can result in the tuning of the reaction properties for maintaining or enhancing polymerization performance. This work provides a blueprint for the application of a user-friendly, functional group-tolerant ROAMP to make ethynylene-linked polymeric materials.

## Supplementary Material



## Notes

Supplemental figures, experimental procedures, and characterization of substrates (PDF)
