# Supplementary material for: Ring-Opening Alkyne Metathesis Polymerization Catalyzed by a Bench-Stable Rhenium Complex
Source: J Am Chem Soc. 2026 Jan 16;148(5):5566–73. doi: 10.1021/jacs.5c19886 (PMC12903850; doi:10.1021/jacs.5c19886)
Supplement: Supplementary file 1 [file ja5c19886_si_001.pdf]

Supporting Information

Ring-opening Alkyne Metathesis Polymerization Catalyzed by a Bench Stable Rhenium Complex

Yinuo Zheng,[‡,a] Ruby L. Y. Chan, [‡,a] Somin Cha,[b] Gregory I. Peterson,[b] Jie Huang,[c] Guochen Jia,[c] Pauline Chiu*[,a] and Antonio Rizzo*[,a]

a Department of Chemistry, and State Key Laboratory of Synthetic Chemistry, The University of Hong Kong, Pokfulam Road, 999077, Hong Kong, P. R. China

b Department of Chemistry and Research Institute of Basic Science, Incheon National University, Incheon 22012, Republic of Korea

c Department of Chemistry, The Hong Kong University of Science and Technology, Clear Water Bay, Kowloon, 999077, Hong Kong, P. R. China

email: anrich@hku.hk (AR), pchiu@hku.hk (PC)

Table of Contents

1. General Information **4**

1.1. Abbreviations 5

2. Catalyst and monomers **6**

2.1. Monomer M1 preparation 7

2.2. Monomer M2 preparation 9

2.3. Monomer M3 preparation 11

2.4. Monomer M4 preparation 14

3. Polymerization Protocols **15**

3.1. Standard Polymerization Protocol 15

3.1.1. P1 15

3.1.2. P2 16

3.1.3. P3 16

3.1.4. P4 17

3.1.5. SEC Traces for Polymers 18

3.1.6. Linear Plots of M_n versus M/I x conversion 19

3.2. Block Copolymer Polymerization 19

3.2.1. P2-*b*-P1 19

3.2.2. P1-*b*-P4 22

3.3. Conversion vs Time Plot Experiment on 1 mmol scale 24

4. ^{1}H NMR and ^{13}C NMR Assignment **28**

5. Initiation and Termination Study **36**

5.1. Initiation Study 36

5.2. Termination Study 38

6. DSC Analysis **46**

7. Hydrolysis of P4 to P2 .... **48**

8. Additional Figures **49**

9. NMR Spectra **51**

9.1 New Compounds NMR Data 51

9.2 Polymers NMR Data 56

10. References 62

1. General Information

Unless specified otherwise, all reactions were performed in oven-dried round-bottomed flasks or vials under a positive pressure of dry argon gas. Reactions were monitored by thin layer chromatography (TLC) using Merck silica gel plates, Kieselgel 60 F_{254} with 0.2 mm thickness. Components were visualized by illumination with 254 nm UV light and/or staining. Flash column chromatography was performed with Merck silica gel 60 (230–400 mesh ASTM).

Unless specified otherwise, chemicals and solvents were purchased and used as received. Anhydrous dioxane (GR grade) was distilled over CaH_2; Anhydrous toluene was dried by passing through drying columns; Anhydrous dichloroethane (DCE) was purchased from Energy Chemicals and used as received; Anhydrous acetonitrile (ACN) was purchased Energy Chemicals used as received. All the solvents for polymerization (dioxane, toluene, DCE, acetonitrile) were freshly degassed with argon for over 15 mins before reaction set-up. Glasswares were dried in an oven (160 °C) overnight and cooled down under dry argon.

NMR: All ^1H, ^{13}C NMR spectra were recorded in $CDCl_3$, with the residual solvent peak as internal standards at ambient temperature on a Bruker 400, 500, or 600 mHz Fourier Transform Spectrometer operating at 400, 500, or 600 mHz for ^1H, and at 100, 125 or 150 mHz for ^{13}C respectively. Spectral features were designated as follows: s = singlet, d = doublet, t = triplet, q = quartet, quint = quintet, m = multiplet and br = broad. Chemical shifts are shown in ppm.

MS: High-resolution ESI-MS measurements were performed on a Bruker impact II high-resolution LC-QTOF mass spectrometer. Accurate masses from high-resolution mass spectra were reported for the molecular ion $[M+H]^+$.

SEC: The SEC setup utilized a Schambek high-pressure liquid chromatography pump (model: S9425ISA), Schambek autosampler (model: S6250) with a sample loop volume of 100 µL, and three PSS SDV size-exclusion columns maintained at 35 °C. For molecular weight characterization, Schambek (model: S2020A) refractive index (RI) detector (maintained at 35 °C) was used. The mobile phase consisted of HPLC-grade THF with a 1.0 mL/min flow rate. Molecular weights were determined via a calibration curve generated from PS standards.

DSC: Measurements of glass transition temperature were completed with a Mettler Toledo DSC 3+ using aluminum pans.

1.1. Abbreviations

DP – degree of polymerization; M_n – number-average molecular weight; T_g – glass transition temperature; *Đ*– dispersity; DCE – dichloroethane; THF – tetrahydrofuran; DBU –1,8-Diazabicyclo [5.4.0]undec-7-ene; 4DMAP– 4-Dimethylaminopyridine.

2. Catalyst and monomers

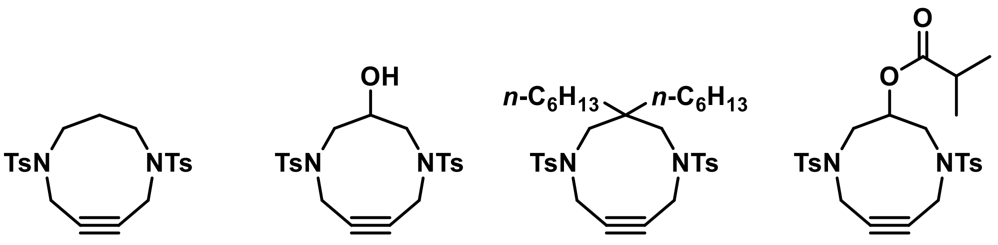

M1 **M2** **M3** **M4**

Reported *Reported* *New* *New*

Figure S1. Monomers used in this study.

Compounds **M1**[1] and **M2**[2] were previously reported on a smaller scale via a two-fold Nicholas reaction[3]. We prepared them by modifying the reported procedures to accommodate the larger scale. Compound **M3** was synthesized by the same two-fold Nicholas reaction. Compound **M4** was synthesized by derivatization step of **M2**.

Monomers **M1**, **M2** and **M4** are white solids whose differ in their solubility: **M1** is sparsely soluble (see Section 7., full dissolution at ca. 70 °C), **M2** is less soluble (full dissolution at ca. 90 °C), while **M4** completely dissolves in dioxane at room temperature. Monomer **M3** is very viscous oil that dissolves at room temperature in toluene or dioxane. Qualitatively, the observed solubility trend is **M3**>**M4**>**M1**>**M2**.

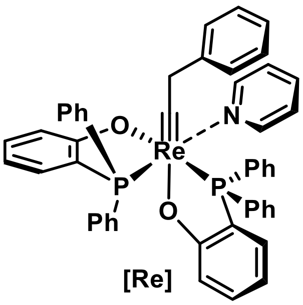

[Re]

Figure S2. Catalyst used in this study.

Complex **[Re]**[4] was synthesized as previously reported on a gram scale and stored in a vial under air without Argon coating.

2.1. Monomer M1 preparation

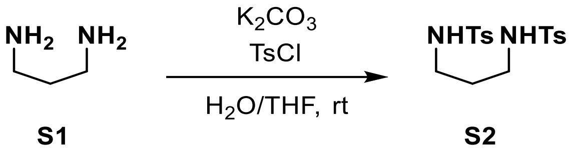

Compound **S1** (5.0 g, 67.5 mmol) and K_2CO_3 (26.1 g, 188.9 mmol) were dissolved in water (100 mL). A THF solution of 4-Me-benzenesulfonyl chloride (TsCl, 30.9 g, 161.9 mmol in 75 mL of THF) was slowly added to this solution using a dropping funnel under air. The reaction mixture was stirred for 12 h. Then, the THF was removed under reduced pressure and the product was precipitated by ice/H_2O addition, filtered and recrystallized from EtOAc/hexanes at 80 °C to give compound **S2** (19.5 g, 51.8 mmol, 76% yield) as a white solid.
The spectroscopical analysis matched the reported literature.[5]

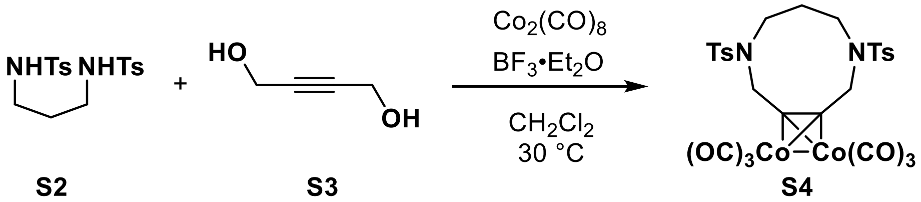

To a solution of **S3** (1.03 g, 12.0 mmol) in dry CH_2Cl_2 (200 mL) was added solid $Co_2(CO)_8$ (4.27 g, 12.5 mmol) at 30 °C and the mixture was stirred for 1 h. Then, the reaction mixture was diluted with additional dry CH_2Cl_2 (500 mL), solid **S2** (3.82 g, 10.0 mmol) was added followed by $BF_3·OEt_2$ (4.9 mL, 40 mmol). The reaction progress was monitored by TLC. Upon completion (ca. 2h), the reaction mixture was quenched by the addition of aqueous sat. $NaHCO_3$ (no gas evolution was observed), the phases were separated. The aqueous phase was extracted with CH_2Cl_2 three times, and the combined organic phase was dried over $MgSO_4$, filtered and the solvent was removed under reduced pressure. The crude dark-red solid **S4** residue was carried on to the next reaction without further purification.

There are several reported methods to decomplex the cobalt from the alkyne. (CAN: **[1]**; TBAF:[6]**;** polyamines:**[7]**) We found that on scale the $Fe(NO_3)_3•(H_2O)_9$ method worked most reliably.[8]

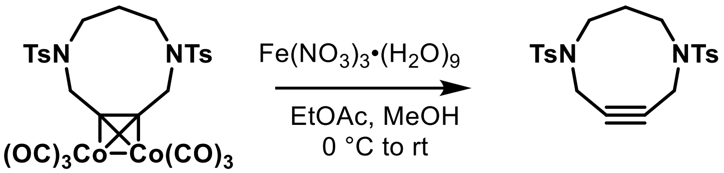

S4 **M1**

Crude **S4** was dissolved in MeOH (170 mL) and EtOAc (50 mL), and cooled to 0 °C. Solid $Fe(NO_3)_3•(H_2O)_9$ (20.2 g, 50 mmol) was added in aliquots over 5 min. The reaction progress was monitored by TLC. Upon completion (ca. 12h), the solvent was removed under reduced pressure and the residue partitioned between H_2O and CH_2Cl_2. The phases were separated, and the water phase was extracted two times with CH_2Cl_2. The combined organic phases were dried over $MgSO_4$, filtered and the solvent removed under reduced pressure. The dark residue was dissolved in a minimal amount of CH_2Cl_2 (ca. 5 mL) and the solution was slowly added to stirring hexane (ca. 200 mL). The precipitated off-white solid was filtered and purified by flash column chromatography (CH_2Cl_2/Hex = 5:1) to afford **M1** as a white solid (3.17 g, 73% yield over two steps).

The spectroscopical analysis matched the reported literature.[1]

Representative ^{1}H NMR of M1 (400 MHz, $CDCl_3$).

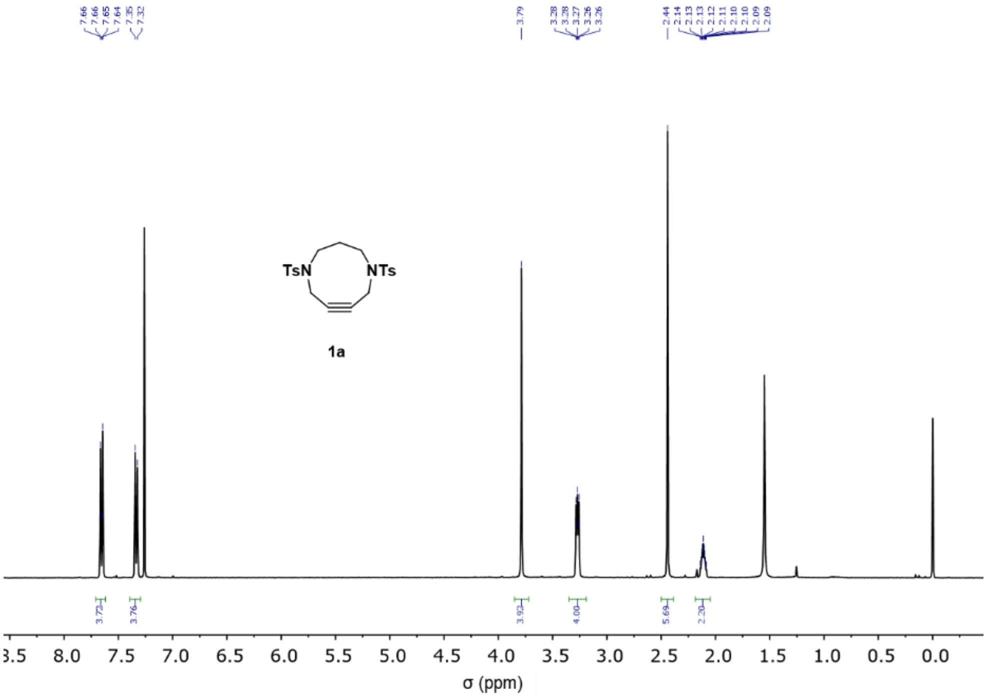

2.2. Monomer M2 preparation

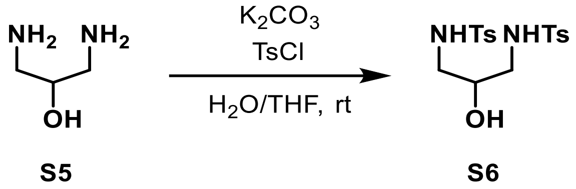

Compound **S5** (5.0 g, 55.5 mmol) and K_2CO_3 (21.5 g, 155.3 mmol) were dissolved in water (100 mL). A THF solution of 4-Me-benzenesulfonyl chloride (TsCl, 25.38 g, 133.2 mmol in 75 mL of THF) was slowly added to this solution using a dropping funnel under air. The reaction mixture was stirred for 12 h. Then, the THF was removed under reduced pressure and the product was precipitated by ice/H_2O addition, filtered and recrystallized from EtOAc/hexanes at 80 °C to give compound **S6** (17.0 g, 42.7 mmol, 77% yield) as a white solid.
The spectroscopical analysis matched the reported literature.[9]

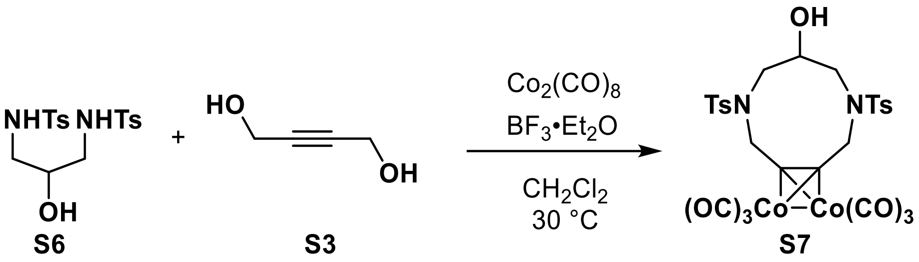

To a solution of **S3** (1.03 g, 12.0 mmol) in dry CH_2Cl_2 (200 mL) was added solid $Co_2(CO)_8$ (4.27 g, 12.5 mmol) at 30 °C and the mixture was stirred for 1 h. Then, the reaction mixture was diluted with additional dry CH_2Cl_2 (500 mL), solid **S6** (3.98 g, 10.0 mmol) was added followed by $BF_3{\cdot}OEt_2$ (4.9 mL, 40 mmol). The reaction progress was monitored by TLC. Upon completion (ca. 2h), the reaction mixture was quenched by the addition of aqueous sat. $NaHCO_3$ (no gas evolution was observed), the phases were separated. The aqueous phase was extracted with CH_2Cl_2 three times, and the organic phase dried over $MgSO_4$, filtered and the solvent was removed under reduced pressure. The crude dark red solid **S7** residue was carried on to the next reaction without further purification.

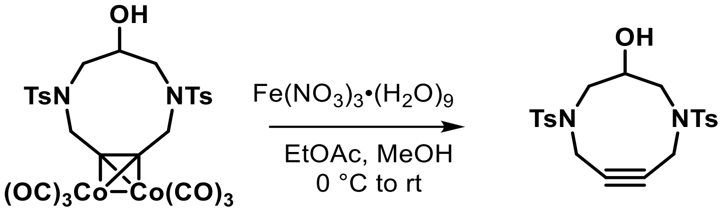

S7 **M2**

Crude **S7** was dissolved in MeOH (170 mL) and EtOAc (50 mL) and cooled to 0 °C. Solid $Fe(NO_3)_3$•$(H_2O)_9$ (20.2 g, 50 mmol) was added in aliquots over 5 min. The reaction progress was monitored by TLC. Upon completion (ca. 12h), the solvent was removed under reduced pressure and the residue partitioned between H_2O and CH_2Cl_2. The phases were separated, and the water phase was extracted two times with CH_2Cl_2. The combined organic phases were dried over $MgSO_4$, filtered and the solvent was removed under reduced pressure. The dark residue was dissolved in a minimal amount of CH_2Cl_2 (ca. 5 mL) and the solution was slowly added to stirring hexane (ca. 200 mL). The precipitated off-white solid was filtered and purified by flash column chromatography (pure CH_2Cl_2) to afford **M2** as a white solid (3.28 g, 73% yield over two steps). The spectroscopical analysis matched the reported literature.[2]

Representative ^1H NMR of M2 (400 MHz, $CDCl_3$).

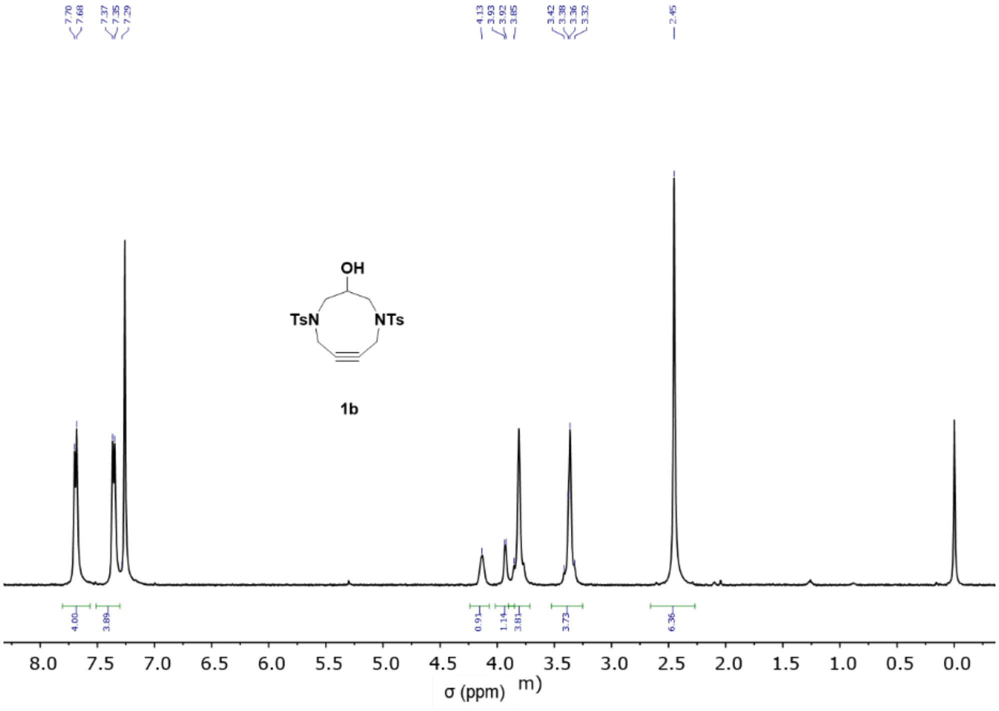

2.3. Monomer M3 preparation

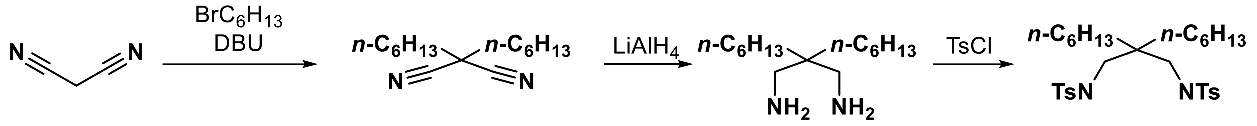

S8 **S9** **S10** **S11**

Dimalonitrile **S8** (4.00 g, 60.55 mmol.) was dissolved in CH_3CN (90.0 mL, technical grade) followed by 1,8-diazabicyclo[5.4.0]undec-7-ene (DBU, 20.28 g, 133.21 mmol., 20.28 mL) and 1-bromohexane (21.99 g, 133.21 mmol., 18.8 mL). The dark mixture was refluxed at 80 °C and stirred overnight. Upon completion (ca. 12h), the solvent was removed under reduced pressure, the residue dissolved in Et_2O/hexanes (2:8, 100 mL) and filtered on a silica pad (sintered glass funnel 10 cm diameter, packed 2 cm height with the same eluent mixture). The solvent was removed under reduced pressure and the crude product **S9** was isolated as a colorless oil (13.90 g, 59.3 mmol., 98% yield).

^{1}H NMR (500 MHz, $CDCl_3$) δ 1.98 – 1.83 (m, 4H), 1.66 (ddt, *J* = 12.2, 8.1, 6.0 Hz, 4H), 1.45 – 1.34 (m, 4H), 1.33 (dd, *J* = 6.8, 4.0 Hz, 8H), 0.90 (td, *J* = 6.8, 5.8, 3.2 Hz, 6H) ppm.

^{13}C NMR (101 MHz, $CDCl_3$) δ 116.0, 38.0, 31.4, 28.7, 25.7, 22.6, 14.1 ppm.

HRMS (ESI, m/z): calculated for $C_{15}H_{27}N_2^+$ (M+H$^+$) 235.2169; Found: 235.2168.

Lithium aluminum hydride (4.86 g, 127.1 mmol.) was mixed with Et_2O (80 mL, technical grade) under nitrogen and cooled in an ice bath. A solution of **S9** (5.00 g, 21.3 mmol.) in Et_2O (20 mL) was added dropwise under vigorous stirring. The ice bath was then removed, and the reaction mixture was allowed to warm to room temperature. The reaction progress was monitored by TLC. Upon completion (ca. 12h), the reaction was cooled using an ice bath, and then 5.0 mL water was added carefully to quench excess hydride. Following, 5.0 mL of 1 M NaOH was added, and the mixture was allowed to warm to room temperature. The solids were filtered, washed with CH_2Cl_2, and the solvent removed under reduced pressure. The crude diamine **S10** was isolated as light-yellow oil (4.57 g, 88% yield).

^{1}H NMR (600 MHz, CD_3OD) δ 2.48 (s, 4H), 1.34 (qd, *J* = 10.4, 8.7, 5.2 Hz, 12H), 1.21 (d, *J* = 2.1 Hz, 8H), 0.93 (t, *J* = 6.7 Hz, 6H) ppm.

^{13}**C NMR** (126 MHz, CD_3OD) δ 46.1, 41.4, 33.3, 33.0, 31.3, 23.9, 23.7, 14.4 ppm.

HRMS (ESI, m/z): calculated for $C_{15}H_{35}N_2^+$ (M+H$^+$) 243.2795; Found: 243.2793.

The crude diamine **S10** (4.57 g, 18.9 mmol) and K_2CO_3 (7.29 g, 52.8 mmol) were dissolved in water/THF (50 mL/ 10 mL). A THF solution of 4-Me-benzenesulfonyl chloride (TsCl, 8.62 g, 45.3 mmol in 30 mL of THF) was slowly added to this solution using a dropping funnel under air. The reaction mixture was stirred for 12 h. Then, the THF was removed under reduced pressure, and the residue partitioned between water and CH_2Cl_2.The phases were separated, the organic phase was dried over $MgSO_4$, and the solvent removed under reduced pressure. The solid residue was recrystallized from EtOAc/hexanes at 80 °C to give compound **S11** (7.2 g, 13.1 mmol, 69% yield) as a white solid.

^{1}**H NMR** (500 MHz, $CDCl_3$) δ 7.76 (d, J = 8.1 Hz, 4H), 7.32 (d, J = 7.6 Hz, 4H), 5.43 – 5.05 (m, 2H), 2.68 (d, J = 7.1 Hz, 4H), 2.45 (d, J = 1.8 Hz, 6H), 1.32 – 1.13 (m, 12H), 1.09 (s, 8H), 0.87 (t, J = 7.1 Hz, 6H) ppm.

^{13}**C NMR** (126 MHz, $CDCl_3$) δ 143.6, 137.0, 129.9, 127.1, 46.6, 40.5, 31.8, 31.4, 30.0, 22.8, 22.5, 21.7, 14.2 ppm.

M.p. 159.1 – 160.0 °C

HRMS (ESI, m/z): calculated for $C_{29}H_{47}N_2O_4S_2^+$ (M+H$^+$) 551.2972; Found: 551.2970.

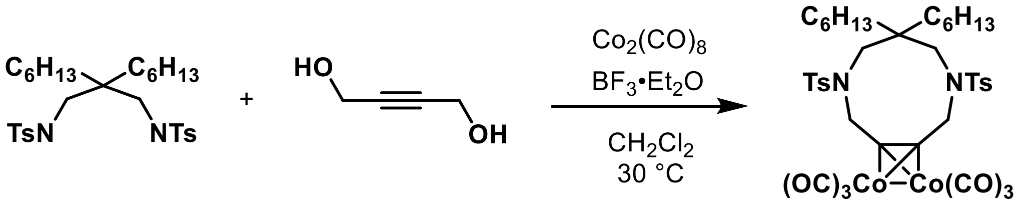

S11 **S3** **S12**

To a solution of **S3** (1.03 g, 12.0 mmol) in dry CH_2Cl_2 (120 mL) was added solid $Co_2(CO)_8$ (4.93 g, 14.4 mmol) at 30 °C and the mixture was stirred for 1.5 h. Then, the reaction mixture was diluted with additional dry CH_2Cl_2 (200 mL), solid **S11** (1.65 g, 3.0 mmol) was added followed by $BF_3{\cdot}OEt_2$ (1.51 mL, 12.0 mmol). The reaction progress was monitored by TLC. Upon completion (ca. 8h), the reaction mixture was quenched by the addition of aqueous $NaHCO_{3sat.}$(no gas evolution was observed), the phases were separated, the organic phase dried over $MgSO_4$,

filtered and the solvent removed under reduced pressure. The crude dark red solid residue was carried on to the next reaction without further purification.

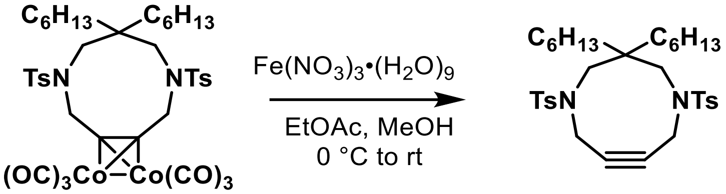

S12 **M3**

Crude **S12** was dissolved in MeOH (100.0 mL, technical grade) and cooled to 0 °C. Solid $Fe(NO_3)_3{\bullet}(H_2O)_9$ (8.08 g, 20.0 mmol.) was added in aliquots over 10 min. The reaction progress was monitored by TLC. Upon completion (ca. 24h), the solvent was removed under reduced pressure and the residue partitioned between H_2O and Et_2O. The phases were separated, and the water phase was extracted two times with Et_2O. The combined organic phases were dried over $MgSO_4$, filtered and the solvent removed under reduced pressure. The dark residue was purified by flash column chromatography (15% Et_2O/hexanes) to afford **M3** as a dense clear oil (0.98 g, 54% yield).

^{1}H NMR (500 MHz, $CDCl_3$) δ 7.67 – 7.61 (m, 4H), 7.36 (d, *J* = 8.0 Hz, 4H), 3.74 (s, 4H), 3.25 (s, 4H), 2.47 (s, 6H), 1.58 (q, *J* = 4.9, 4.4 Hz, 4H), 1.36 (d, *J* = 2.4 Hz, 16H), 1.02 – 0.88 (m, 6H) ppm.

^{13}C NMR (126MHz, $CDCl_3$) δ 144.0, 133.5, 129.9, 127.9, 87.5, 52.9, 42.3, 41.4, 34.7, 31.9, 30.3, 23.4, 22.7, 21.5, 14.1 ppm.

HRMS (ESI, m/z): calculated for $C_{33}H_{49}N_2O_4S_2^+$ (M+H$^+$) 601.3128; Found: 601.3129.

2.4. Monomer M4 preparation

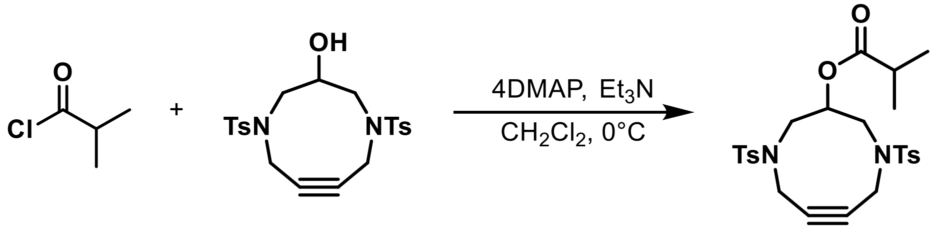

isobutyryl chloride **M2** **M4**

To a solution of **M2** (0.20 g, 0.44 mmol) in dry CH_2Cl_2 (4.0 mL) was added 4DMAP (10.89 mg, 0.089 mmol) and Et_3N (0.19 mL, 1.34 mmol). The reaction mixture was cooled to 0 °C with an ice bath and isobutyryl chloride (0.1 mL, 0.09 mmol) was added dropwise. The reaction progress was monitored by TLC. Upon completion (ca. 2h), the reaction was partitioned between H_2O and CH_2Cl_2. The phases were separated, and the water phase was extracted two times with CH_2Cl_2. The combined organic phases were dried over $MgSO_4$, filtered and the solvent removed under reduced pressure. The crude residue was purified by flash column chromatography (40% EtOAc/hexanes) to afford **M4** as a white solid (0.11 g, 47% yield).

^{1}H NMR (500 MHz, Chloroform-*d*) δ 7.64 (d, 4H), 7.33 (d, *J* = 8.0 Hz, 4H), 5.25 (tt, *J* = 6.8, 3.5 Hz, 1H), 3.82 (s, 4H), 3.57 (dd, *J* = 15.2, 6.7 Hz, 2H), 3.30 (dd, *J* = 15.2, 3.6 Hz, 2H), 2.65 (dq, *J* = 14.0, 7.0 Hz, 1H), 2.43 (s, 6H), 1.22 (d, *J* = 7.0 Hz, 6H) ppm.

^{13}C NMR (126 MHz, Chloroform-*d*) δ 176.4, 144.2, 134.1, 130.0, 127.5, 88.3, 73.2, 47.8, 40.7, 34.0, 21.6, 18.9 ppm.

M.p. 123.9 – 126.3 °C
HRMS (ESI, m/z): calculated for $C_{25}H_{31}N_2O_6S_2^+$ (M+H$^+$) 519.1618; Found: 519.1611.

3. Polymerization Protocols

3.1. Standard Polymerization Protocol

A 2-mL sized screw-cap tube with septum was flame dried and charged with catalyst **[Re]**, monomer **M1-4** (see below for scale) and a magnetic bar. The vial was purged with argon for over 10 mins, and degassed anhydrous solvent was added. (Note: When M/I ratio is over 100:1, catalyst **[Re]** was added as a stock solution in degassed dioxane). The reaction vessel was submerged in a preheated oil bath with the solvent surface below the oil level (for experimental temperature see Table 1, main text) under vigorous stirring. The reaction was quenched by excess 1-(tert-butyl)-4-ethynylbenzene after the desired reaction time and precipitated using the conditions described below. Monomer conversion was calculated from the ^{1}H NMR spectrum of the crude mixture before precipitation.

3.1.1. P1

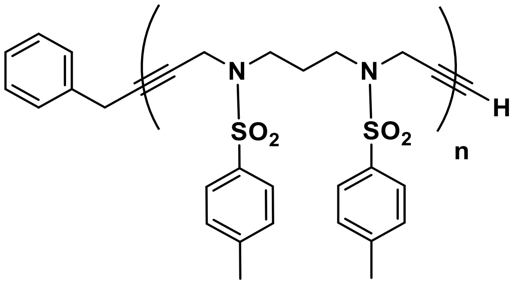

P1

Following the general procedure provided in 3.1.: **[Re]** (depending on feed ratio) and **M1** (86.5 mg, 0.2 mmol) in dioxane (1.0 mL). Precipitation conditions: the crude polymer was dissolved in ca. 0.2 mL of CH_2Cl_2 and added drop wise to MeOH (15.0 mL). **P1** was precipitated and filtered to give a white powder.

^{1}H NMR (600 MHz, $CDCl_3$) δ 7.62 (d), 7.30 (d), 3.87 (s), 3.02 (t), 2.41 (s), 1.70 (m) ppm.

^{13}C NMR (151 MHz, $CDCl_3$) δ 144.1, 135.5, 129.8, 127.8, 78.6, 44.4, 37.1, 26.5, 21.7 ppm.

3.1.2. P2

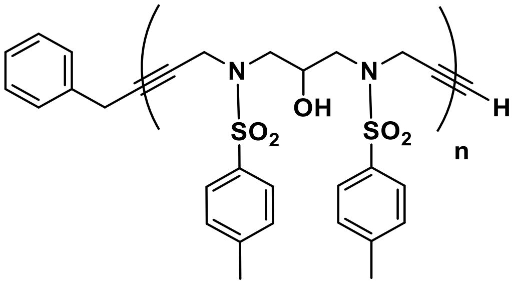

P2

Following the general procedure provided in 3.1.: **[Re]** (depending on feed ratio) and **M2** (89.7 mg, 0.2 mmol) in dioxane (1.0 mL). Precipitation conditions: the crude polymer was dissolved in ca. 0.2 mL of CH_2Cl_2 and added drop wise to MeOH (15.0 mL). **P2** was precipitated and filtered to give a white solid.

^{1}H NMR (600 MHz, $CDCl_3$) δ 7.63 (d), 7.28 (d), 4.03 – 3.88 (m), 3.20 (dt), 3.02 (td), 2.38 (s) ppm.

^{13}C NMR (151 MHz, $CDCl_3$) δ 144.3, 135.1, 129.9, 127.9, 79.1, 69.1, 50.6, 38.9, 21.7 ppm.

3.1.3. P3

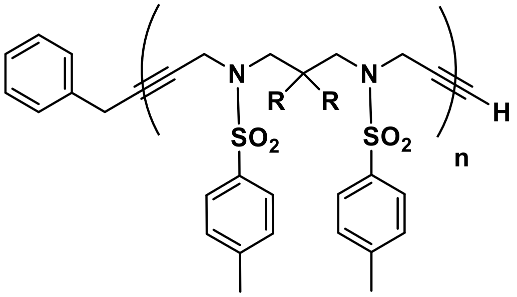

R = *n*-C_6H_{13} **P3**

Following the general procedure provided in 3.1.: **[Re]** (depending on feed ratio) and **M3** (120.2 mg, 0.2 mmol) in dioxane (1.0 mL). Precipitation conditions: the crude polymer was dissolved in ca. 0.2 mL of CH_2Cl_2 and added drop wise to MeOH (15.0 mL). **P3** was precipitated and filtered to give grey solid.

^{1}H NMR (600 MHz, $CDCl_3$) δ 7.69 (d), 7.32 (d), 3.81 (s), 3.15 (s), 2.44 (s), 1.45 – 1.10 (m), 0.89 (t) ppm.

^{13}C NMR (151 MHz, $CDCl_3$) δ 143.8, 135.9, 129.5, 128.2, 79.3, 51.6, 41.8, 39.6, 35.0, 31.9, 30.4, 23.3, 22.9, 21.7, 14.2 ppm.

3.1.4. P4

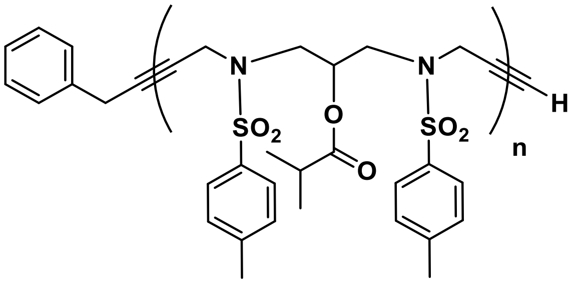

P4

Following the general procedure provided in 3.1.: **[Re]** (depending on feed ratio) and **M4** (51.9 mg, 0.1 mmol) in dioxane (0.5 mL). Precipitation conditions: the crude polymer was dissolved in ca. 0.1 mL of CH_2Cl_2 and added drop wise to MeOH (7.0 mL). **P4** was precipitated and filtered to give a white powder.

^{1}H NMR (600 MHz, $CDCl_3$) δ 7.63 (d), 7.30 (d), 5.14 (dq), 3.91 (d), 3.35 – 3.12 (m), 2.49 (ddd), 2.42 (s), 1.13 (dd) ppm.

^{13}C NMR (151 MHz, $CDCl_3$) δ 176.2, 144.3, 135.6, 129.8, 127.9, 78.8, 69.4, 47.7, 47.6, 38.1, 34.1, 21.7, 19.1 ppm.

3.1.5. SEC Traces for Polymers

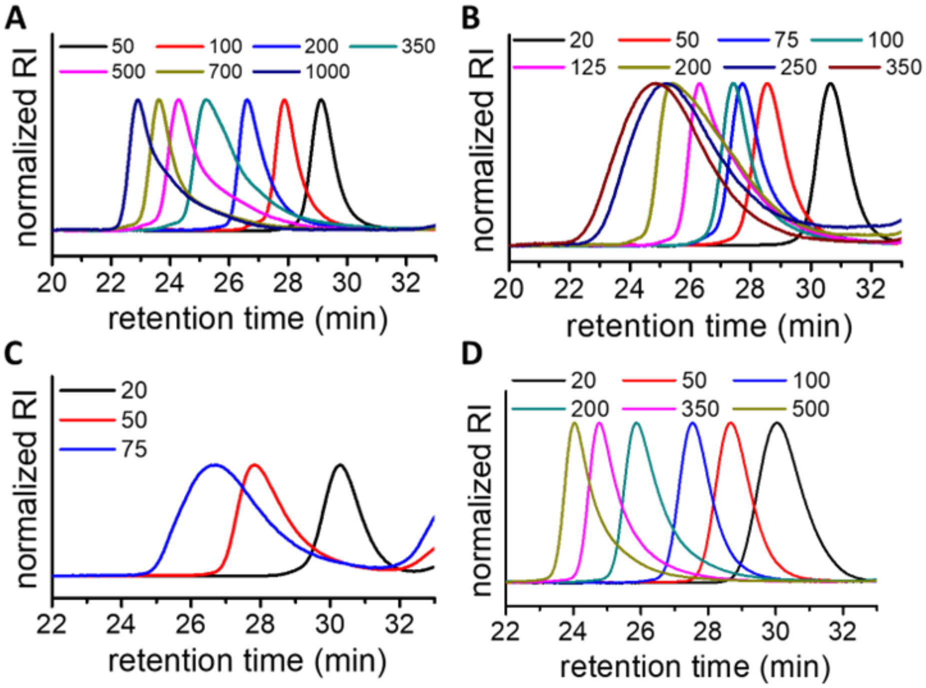

Figure S3. Stacked SEC traces of (A) P1, (B) P2, (C) P3 and (D) P4 which correspond to the entries in Table 1 of the *main text*. Numbers in the legend indicate the targeted degree of polymerization.

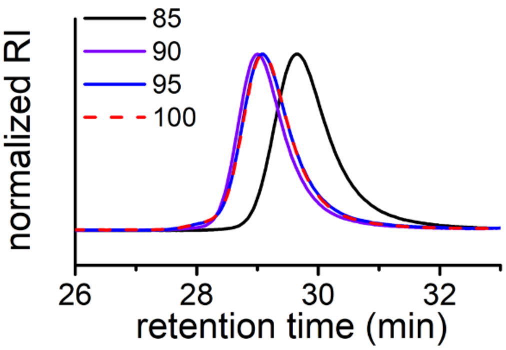

Figure S4. Stacked SEC traces of **P1** which correspond to the entries 6-9 in **Figure 2 (Table)** of the *main text*. Numbers in the legend indicate the temperature (°C) at which the polymerization was conducted.

3.1.6. Linear Plots of M_n versus M/I x conversion

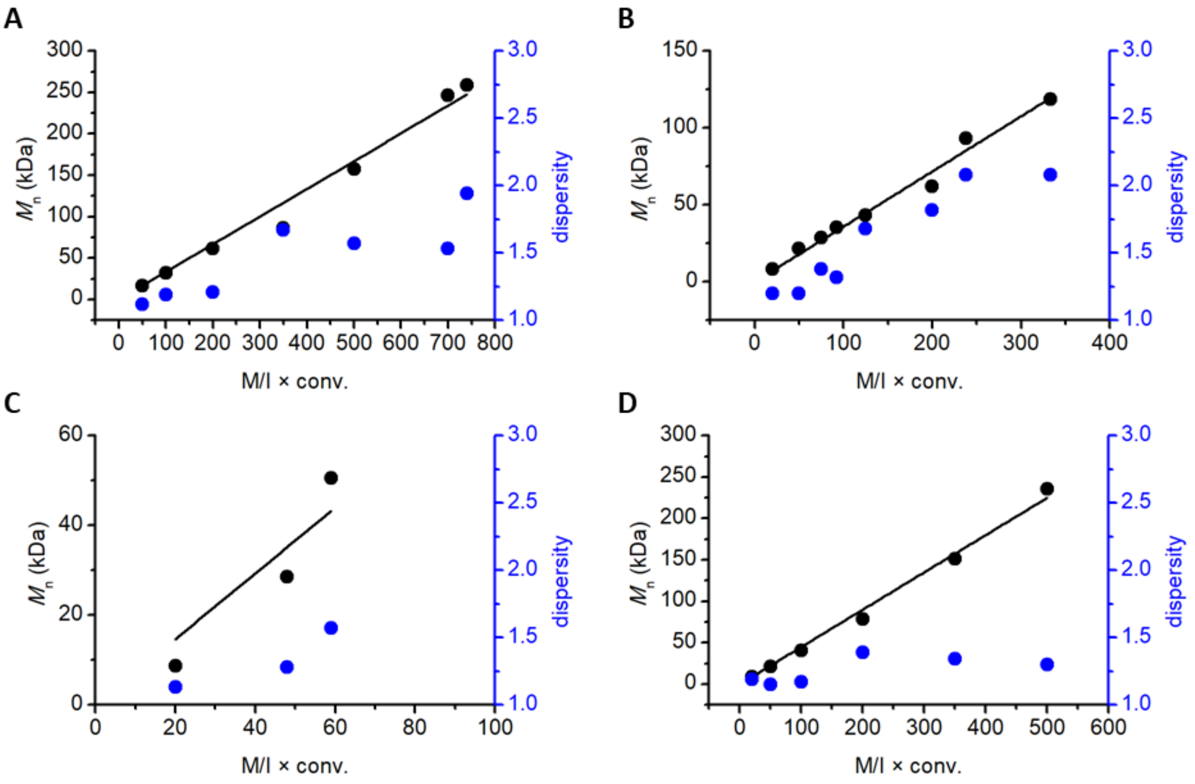

Figure S5. Plots of M_n versus M/I x conversion for (A) **P1**, (B) **P2**, (C) **P3**, and (D) **P4**, which correspond to the entries in **Table 1** of the *main text*. Black circles represent M_n values and blue circles represent $Đ$ values. Linear fits to the M_n data are shown.

3.2. Block Copolymer Polymerization

3.2.1. P2-*b*-P1

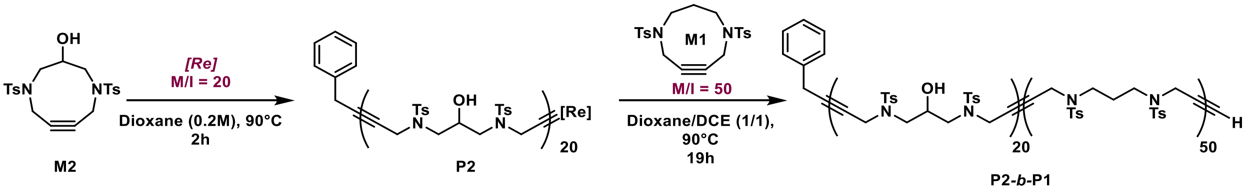

A 5-mL sized test tube with septum was flame dried and charged with catalyst **[Re]** (4.6 mg, 0.005 mmol), monomer **M2** (44.9 mg, 0.1 mmol) and a magnetic bar. The vial was purged with argon for over 10 mins, and degassed anhydrous dioxane (0.5 mL) was added. The reaction vessel was submerged in a preheated oil bath (90 ℃) with the solvent surface below the oil level under vigorous stirring. After 2 h, an aliquot (ca. 50 μL) was removed for analysis (conv. >99%) and a solution of **M1** (108.1 mg, 0.25 mmol) in mixed solvents dioxane-DCE (1:1, 0.75 mL) was added

rapidly (Overall 1.25 mL solvents, Conc.= 0.25 mmol/1.25 mL = 0.2M). The reaction was stirred for 19 h before it was quenched by excess 1-(tert-butyl)-4-ethynylbenzene and the solvent removed under reduced pressure. The crude polymer was dissolved in ca. 0.2 mL of CH_2Cl_2 and added drop wise to MeOH (15.0 mL). **P2-*b*-P1** was precipitated and filtered to give a grey powder. Monomer conversion (>99%) was calculated from the ^{1}H NMR spectrum of the crude mixture before precipitation.

P2: M_n ($Đ$) = 8.3 kDa (1.20).

P2-*b*-P1: M_n ($Đ$) = 29.3 kDa (1.20); Yield (121.7 mg/153.0 mg) = 80%;

^{1}H NMR (600 MHz, $CDCl_3$) δ 7.62 (d), 7.30 (d), 4.03 – 3.90 (m), 3.29 – 3.10 (m), 3.02 (t), 2.41 (s), 1.70 (t) ppm.

^{13}C NMR (151 MHz, $CDCl_3$) δ 144.3, 144.1, 135.5, 135.1, 129.9, 129.8, 127.8, 79.1, 78.6, 69.1, 67.2, 50.6, 44.4, 38.9, 37.1, 31.3, 26.5, 21.7 ppm.

A)

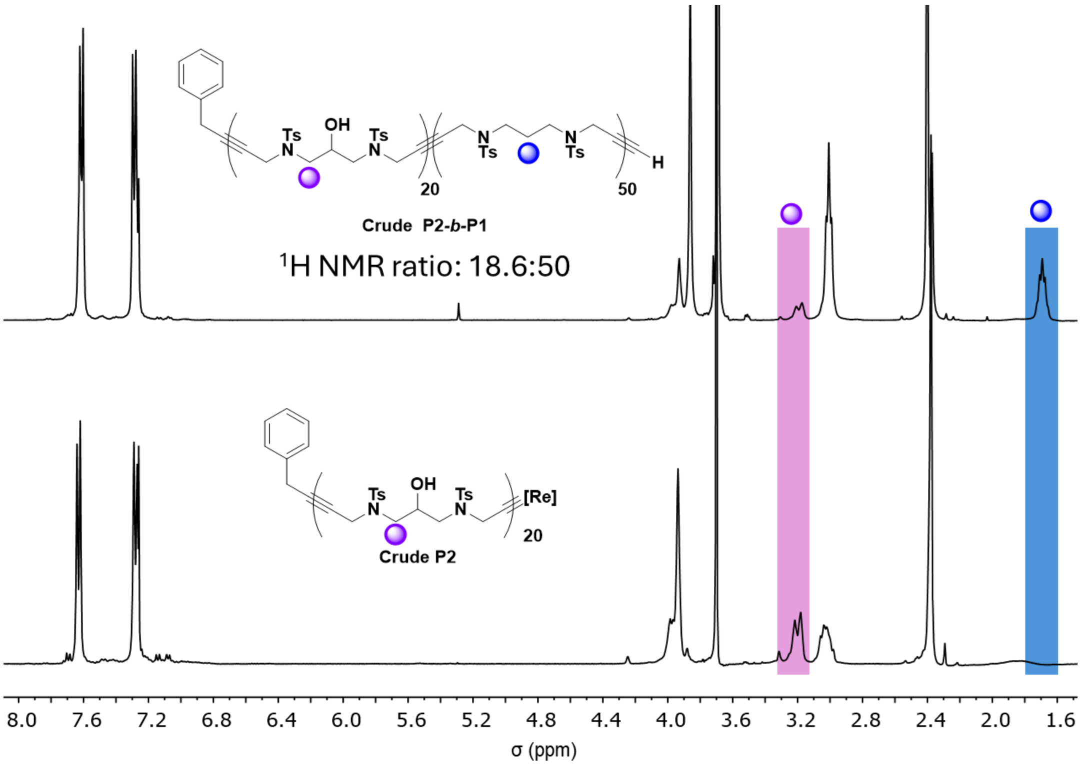

B)

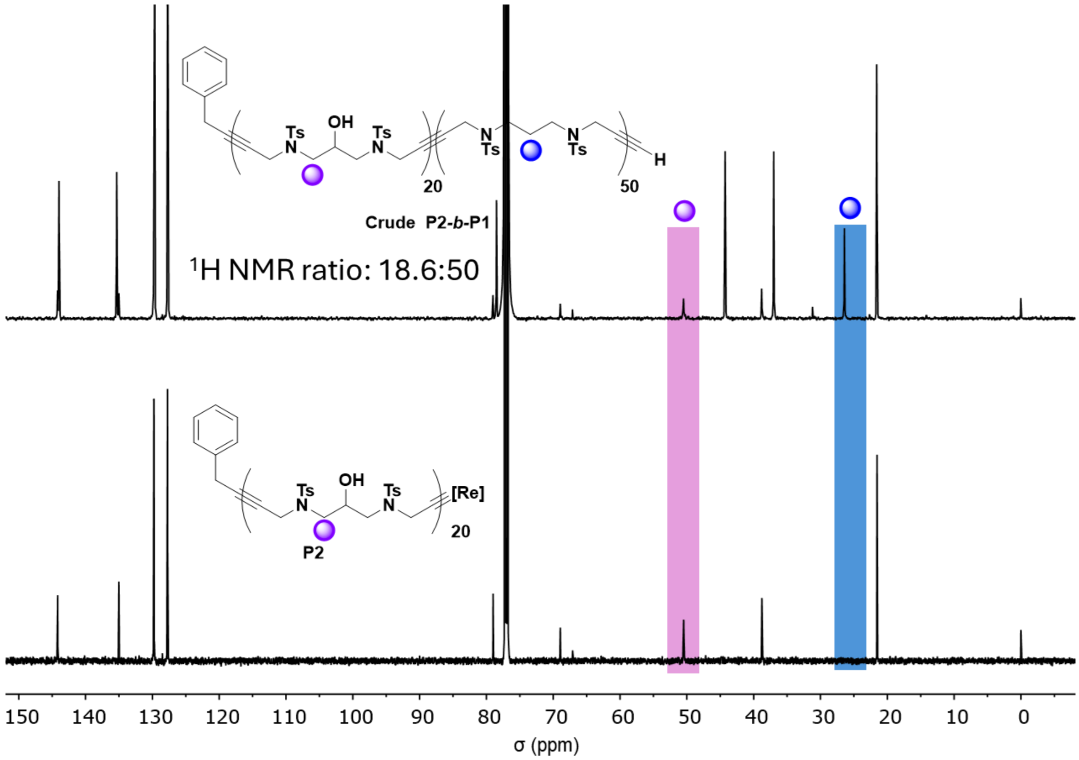

C)

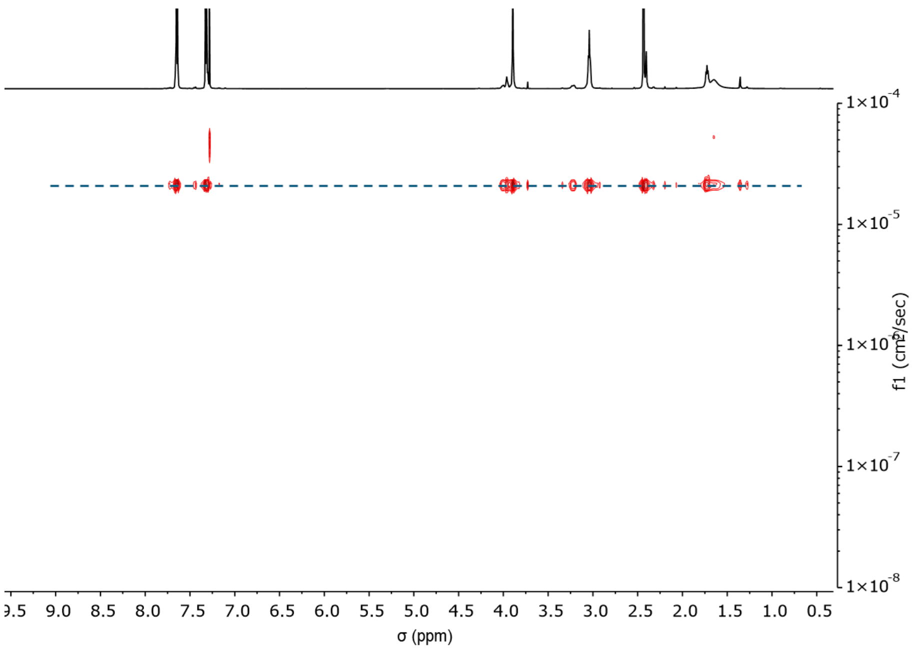

Figure S6. A) Stacked ^{1}H NMR spectra crude aliquot of **P2** (bottom) and crude **P2-*b*-P1** [500 MHz, $CDCl_3$]. B) Stacked ^{13}C NMR spectra of **P2** (bottom) and **P2-*b*-P1** [151 MHz, $CDCl_3$]. C) DOSY NMR of **P2-*b*-P1** [600 MHz, $CDCl_3$].

3.2.2. P1-*b*-P4

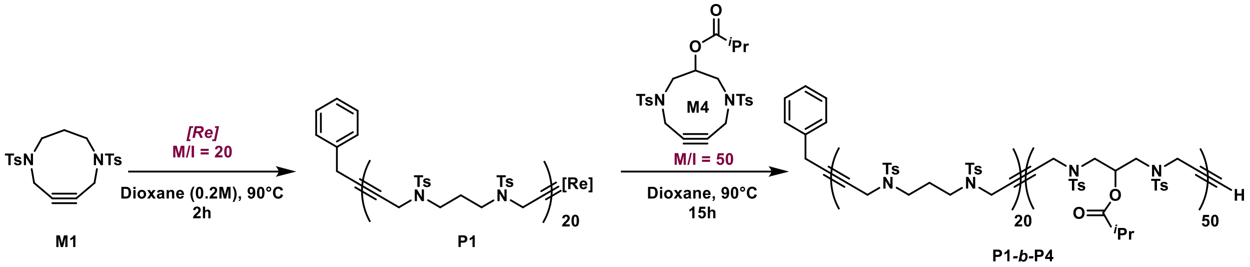

A 5-mL sized test tube with septum was flame dried and charged with catalyst **[Re]** (4.6 mg, 0.005 mmol), monomer **M1** (43.3 mg, 0.1 mmol) and a magnetic bar. The vial was purged with argon for over 10 mins, and degassed anhydrous dioxane (0.5 mL) was added. The reaction vessel was submerged in a preheated oil bath (90 ℃) with the solvent surface below the oil level under vigorous stirring. After 2 h, an aliquot (ca. 50 μL) was removed for analysis (conv. >99%) and a solution of **M4** (129.7 mg, 0.25 mmol) in dioxane (0.75 mL) was added rapidly (Overall 1.25 mL solvents, Conc.= 0.25 mmol/1.25 mL = 0.2M). The reaction was stirred for 15 h before it was quenched by excess 1-(tert-butyl)-4-ethynylbenzene and the solvent removed under reduced pressure. The crude polymer was dissolved in ca. 0.2 mL of CH_2Cl_2 and added drop wise to MeOH (15.0 mL). **P1-*b*-P4** was precipitated and filtered to give a grey powder. Monomer conversion (>99%) was calculated from the ^{1}H NMR spectrum of the crude mixture before precipitation.

P1: M_n ($Đ$) = 6.7 kDa (1.13).

P1-*b*-P4: M_n ($Đ$) = 26.8 kDa (1.20); Yield (158.1 mg/173.0 mg) = 91%;

^{1}H NMR (600 MHz, $CDCl_3$) δ 7.63 (t), 7.30 (d), 5.14 (t), 3.89 (d), 3.38 – 3.17 (m), 3.02 (dt), 2.49 (p), 1.70 (t), 1.13 (d) ppm.

^{13}C NMR (151 MHz, $CDCl_3$) δ 176.2, 144.3, 144.1, 135.6, 135.5, 132.0, 129.8, 129.8, 127.9, 127.8, 125.5, 78.8, 69.4, 67.2, 47.6, 44.4, 38.1, 37.1, 34.1, 31.3, 26.5, 21.7, 19.1 ppm.

A)

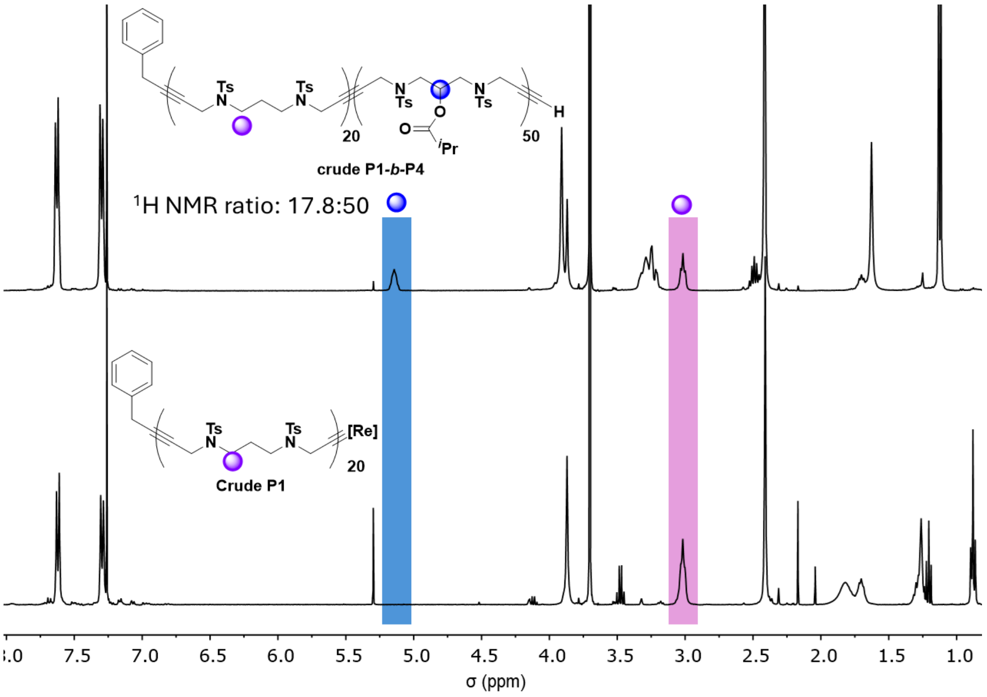
Ts
N
Ts
N
20
N
Ts
O
O
iPr
N
Ts
50
H
crude P1-b-P4
1H NMR ratio: 17.8:50
Ts
N
Ts
N
[Re]
20
Crude P1
7.5 7.0 6.5 6.0 5.5 5.0 4.5 4.0 3.5 3.0 2.5 2.0 1.5 1.0
σ (ppm)

B)

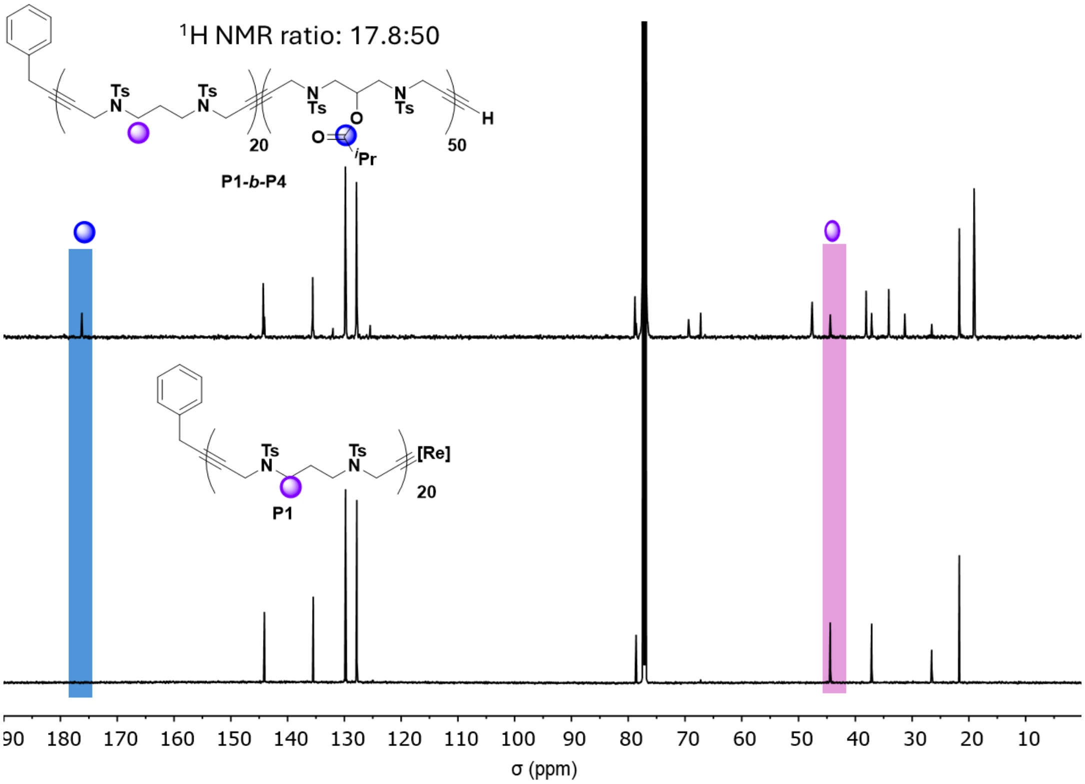
1H NMR ratio: 17.8:50
Ts
N
Ts
N
20
N
Ts
O
O
iPr
N
Ts
50
H
P1-b-P4
Ts
N
Ts
N
[Re]
20
P1
90 180 170 160 150 140 130 120 110 100 90 80 70 60 50 40 30 20 10
σ (ppm)

C)

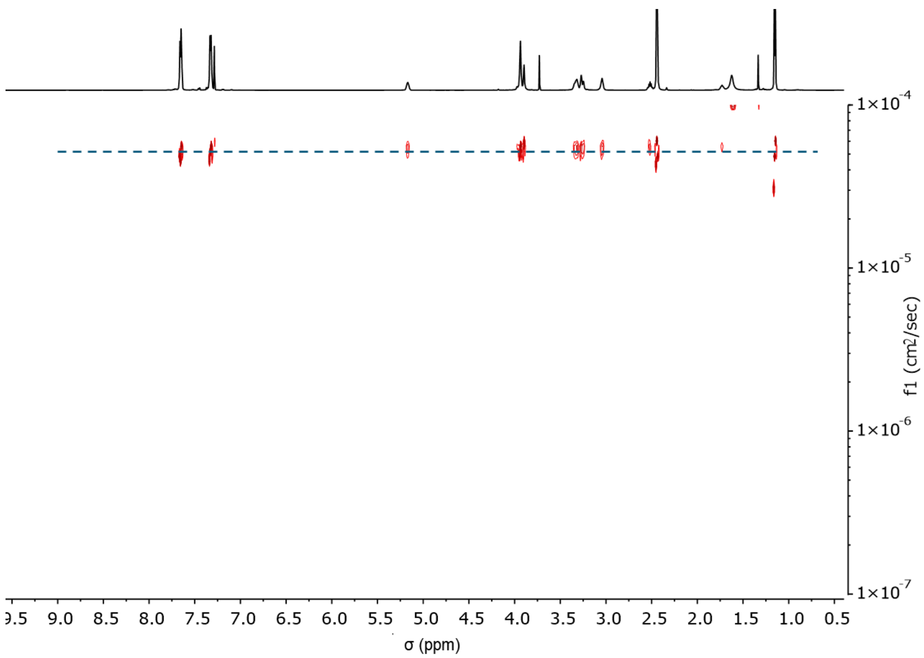

Figure S7. A) Stacked ^{1}H NMR spectra crude aliquot of **P1** (bottom) and crude **P1-*b*-P4** [400 MHz, $CDCl_3$]. B) Stacked ^{13}C NMR spectra of **P1** (bottom) and **P1-*b*-P4** [151 MHz, $CDCl_3$]. C) DOSY NMR of **P1-*b*-P4** [600 MHz, $CDCl_3$].

3.3. Conversion vs Time Plot Experiment on 1 mmol scale

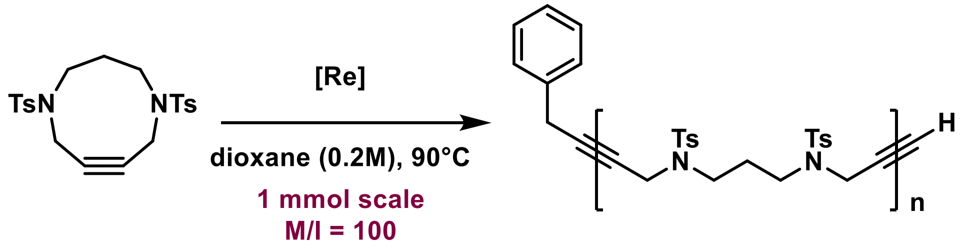

M1 ***P1***

A 25-mL sized screw-cap tube with septum was flame dried and charged with catalyst **[Re]** (9.2 mg), monomer **M1** (432.6 mg) and a magnetic bar. The vessel was purged with argon for >15 mins, and degassed anhydrous dioxane (5 mL) was added. The reaction vessel was submerged in a preheated oil bath (90 °C) with the solvent surface below the oil level under vigorous stirring. The starting time was recorded. Each hour, an aliquot (ca. 50 μL) was removed for analysis. Above 90% conversion, aliquots were removed with a 15-minute frequency. Upon completion, the reaction was quenched by excess 1-(tert-butyl)-4-ethynylbenzene and the solvent removed

under reduced pressure. The crude polymer was dissolved in ca. 2 mL of CH_2Cl_2 and added drop wise to MeOH (50.0 mL). **P1** was precipitated and filtered to give a grey powder. Monomer conversion was calculated from the ^1H NMR spectrum of the crude mixtures.

To calculate the yield of the reaction an adjustment was needed to account for the removal of the aliquots. We removed ca. 50 µL per aliquot 17 times = 0.85 mL. The initial solvent was 5 mL, so 17% of the solution (monomer and polymer) was removed overall.

This makes the theoretical max. yield = 83% of the initial mass balance.

initial mass = 432.6 mg

theoretical max. yield = 83% = 359.0 mg

measured mass = 298.5 mg

Adjusted yield = 83%

a

Entry	Time (h)	Conv. (%)
1	1	9
2	2	22
3	3	33
4	4	42
5	5	51
6	6	59
7	7	68
8	8	76
9	9	83
10	10	90
11	10.25	92
12	10.5	94
13	10.75	95
14	11	95
15	11.25	97
16	11.5	99
17	11.6	99.9

b

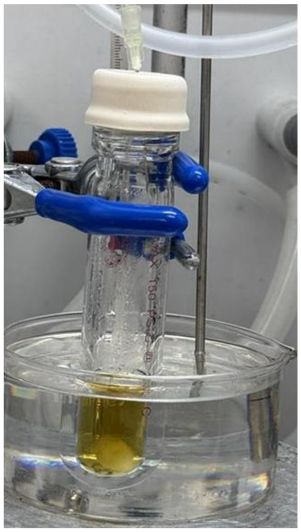

Figure S8. a) Tabulated data from the conversion study. b) Picture of the 1mmol scale polymerization of **M1**.

a

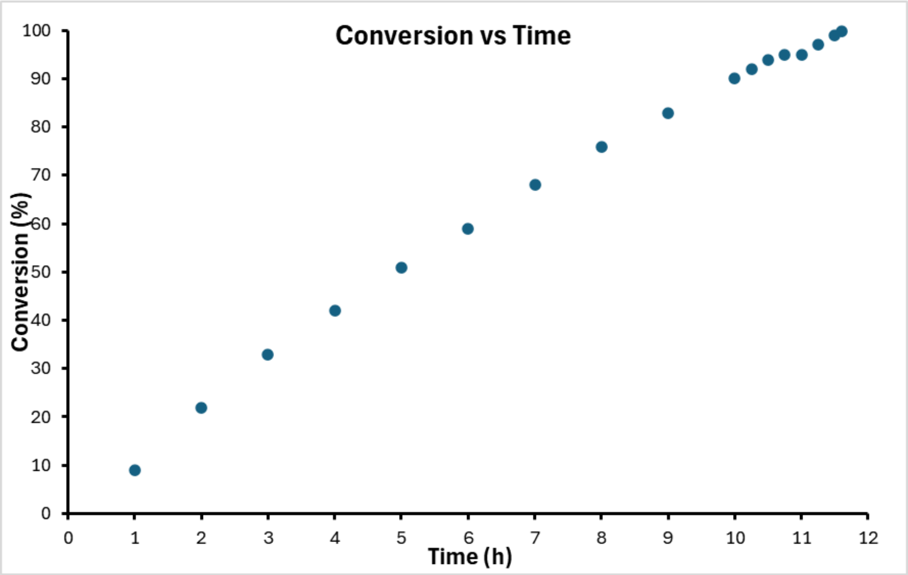

b

Entry	Time (h)	Conv. (%)	M	$\ln([M]/[M_0])$
1	0	0	0.20	0.00
2	1	9	0.18	-0.09
3	2	22	0.16	-0.25
4	3	33	0.13	-0.40
5	4	42	0.12	-0.54
6	5	51	0.10	-0.71

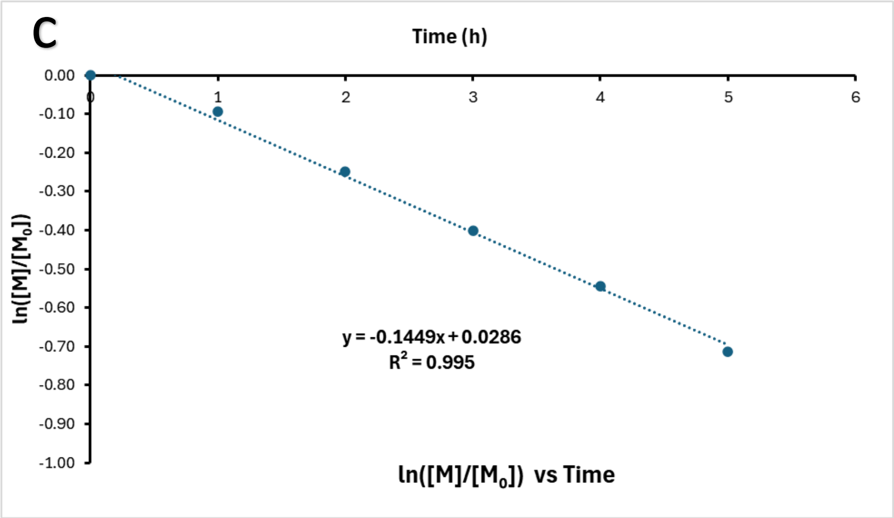

Figure S9. a) Conversion (%) vs time (h) graph of the polymerization of **M1**. b) Tabulated data of calculated **M1** concentration (M) and $\ln([M]/[M_0])$. c) First order kinetic plot of **M1**, $\ln([M]/[M_0])$ vs time (h).

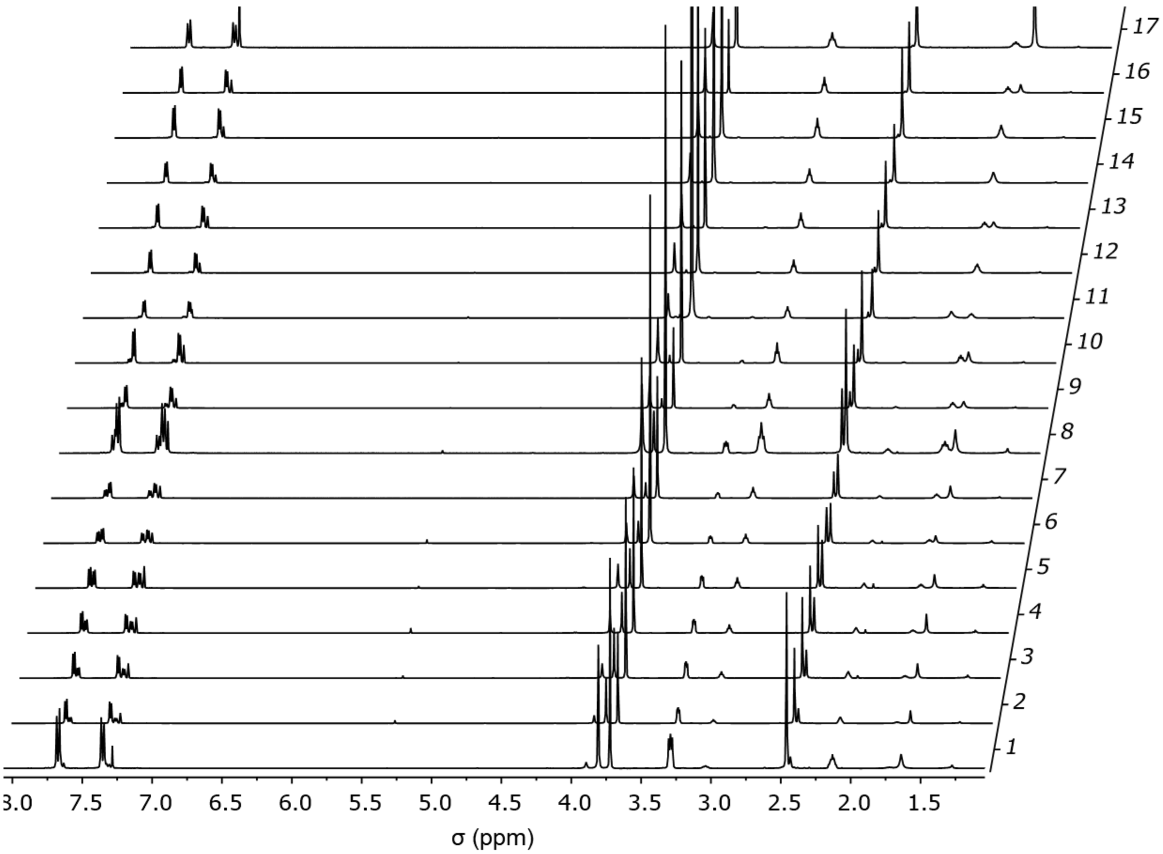

Figure S10. ^{1}H NMR (400 MHz, $CDCl_3$) stacked plot of the polymerization of **M1**. Each entry number (right axis) corresponds to the one in the tabulated data (**Figure S8**).

4. ^{1}H NMR and ^{13}C NMR Assignment

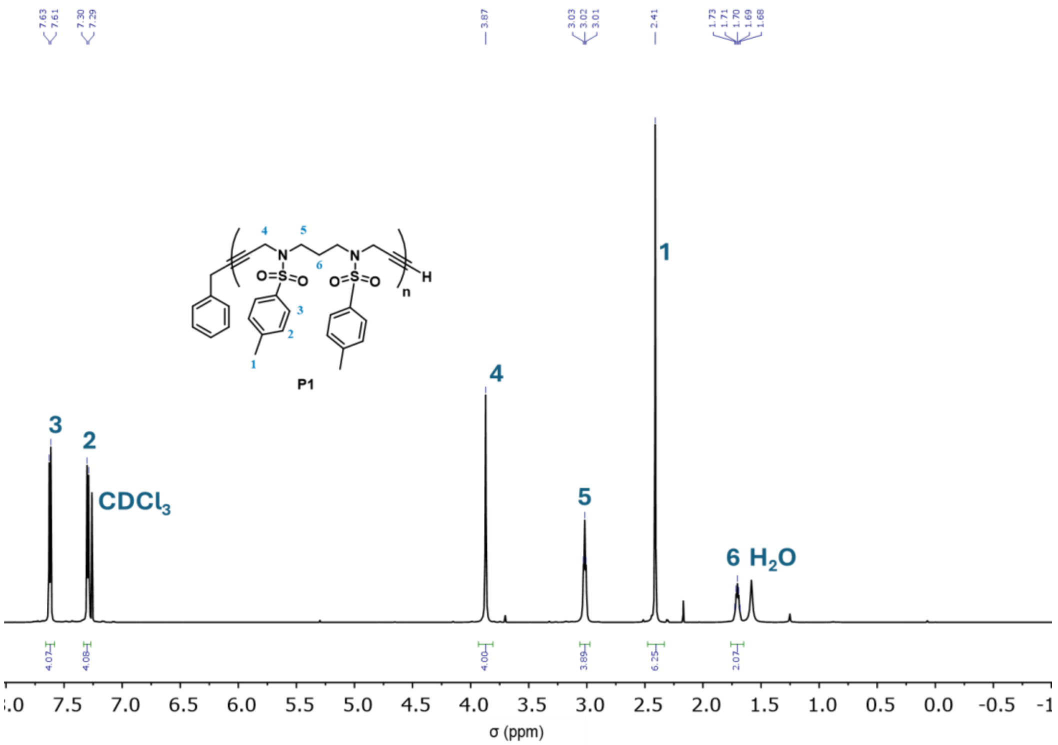

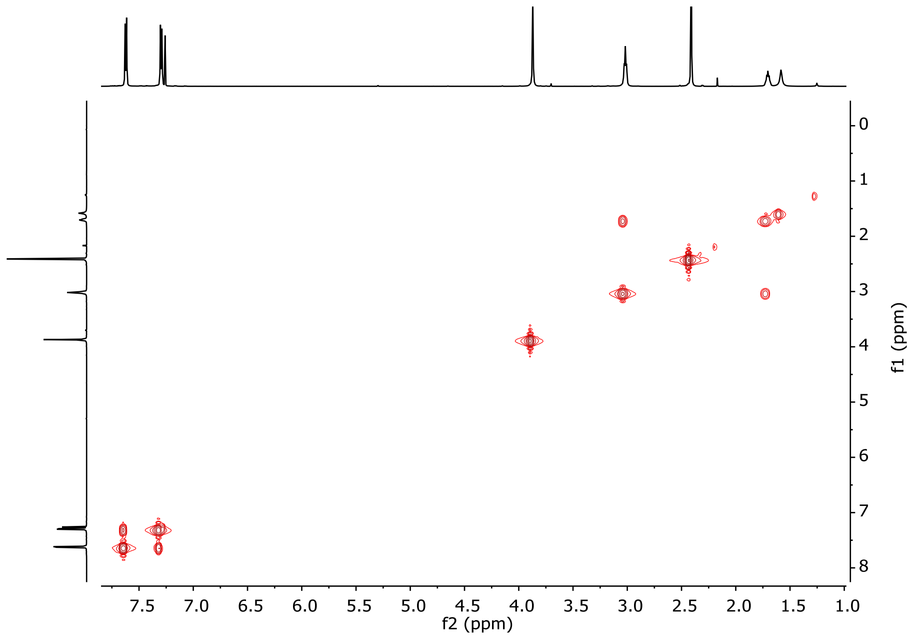

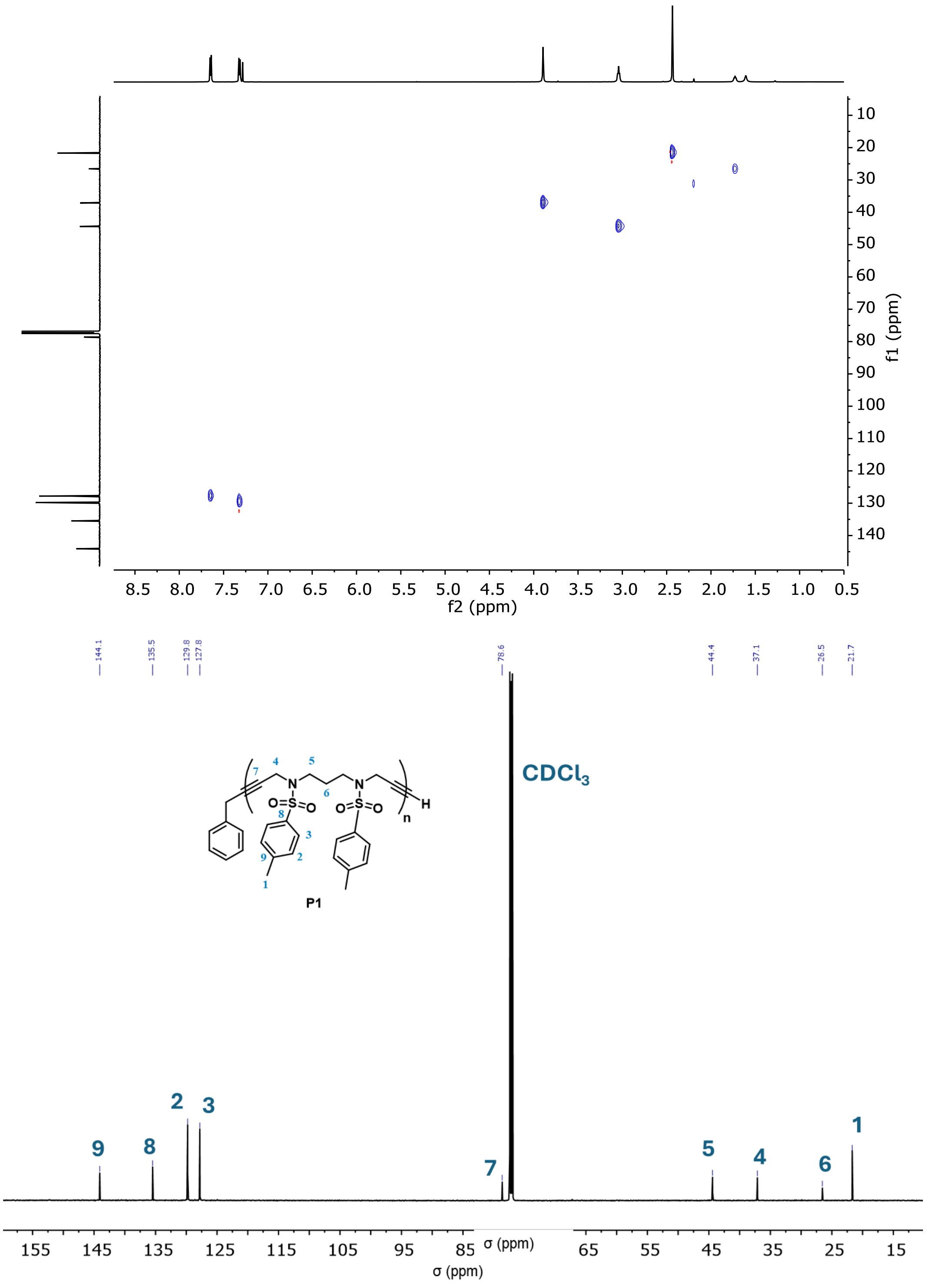

Figure S11. ^{1}H NMR, COSY NMR, HSQC NMR, and ^{13}C NMR spectra for **P1** [600 MHz, $CDCl_3$].

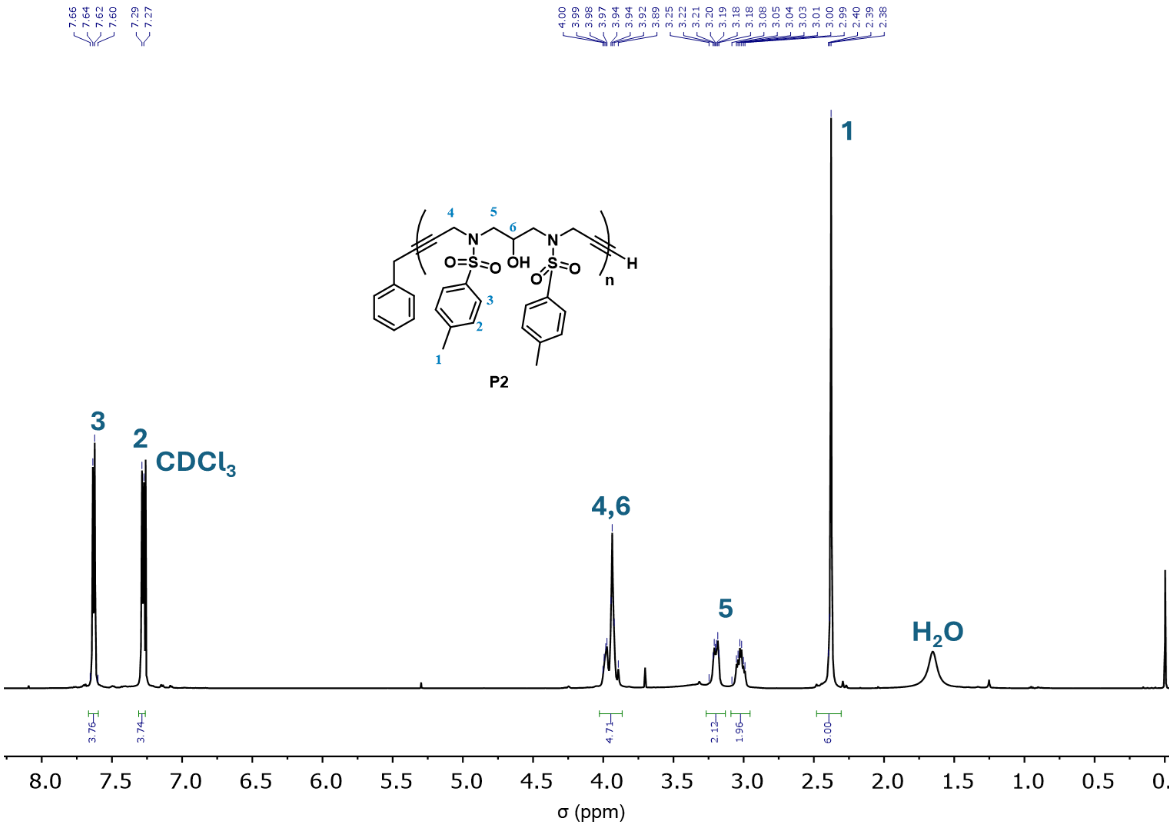

7.66
7.64
7.62
7.60
7.29
7.27
4.00
3.99
3.98
3.97
3.94
3.94
3.92
3.89
3.25
3.22
3.21
3.20
3.19
3.18
3.18
3.08
3.05
3.04
3.03
3.01
3.00
2.99
2.40
2.39
2.38
1
4
5
6
OH
H
n
3
2
1
P2
3
2
CDCl3
4,6
5
H2O
3.76
3.74
4.71
2.13
1.96
6.00
8.0
7.5
7.0
6.5
6.0
5.5
5.0
4.5
4.0
3.5
3.0
2.5
2.0
1.5
1.0
0.5
0.
σ (ppm)

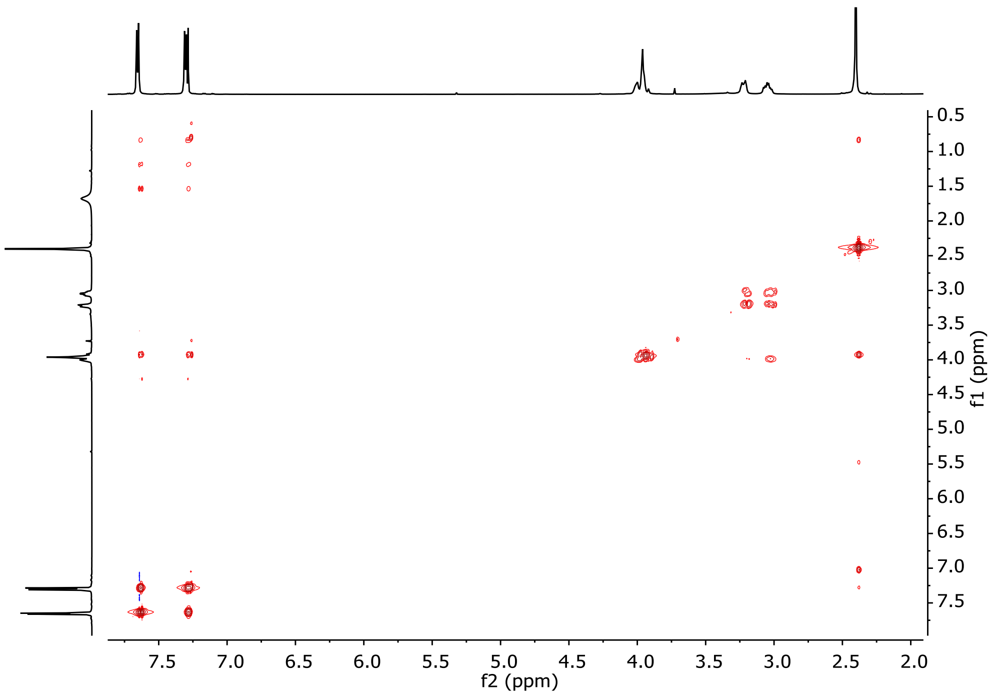

f1 (ppm)
f2 (ppm)

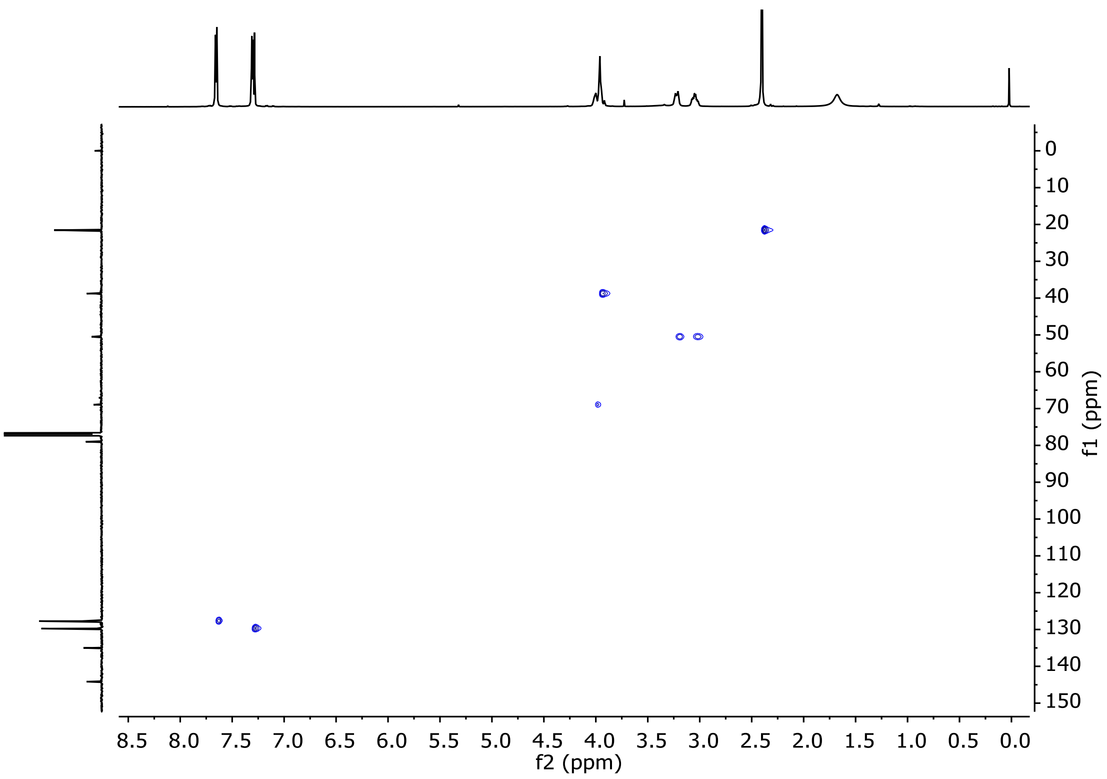

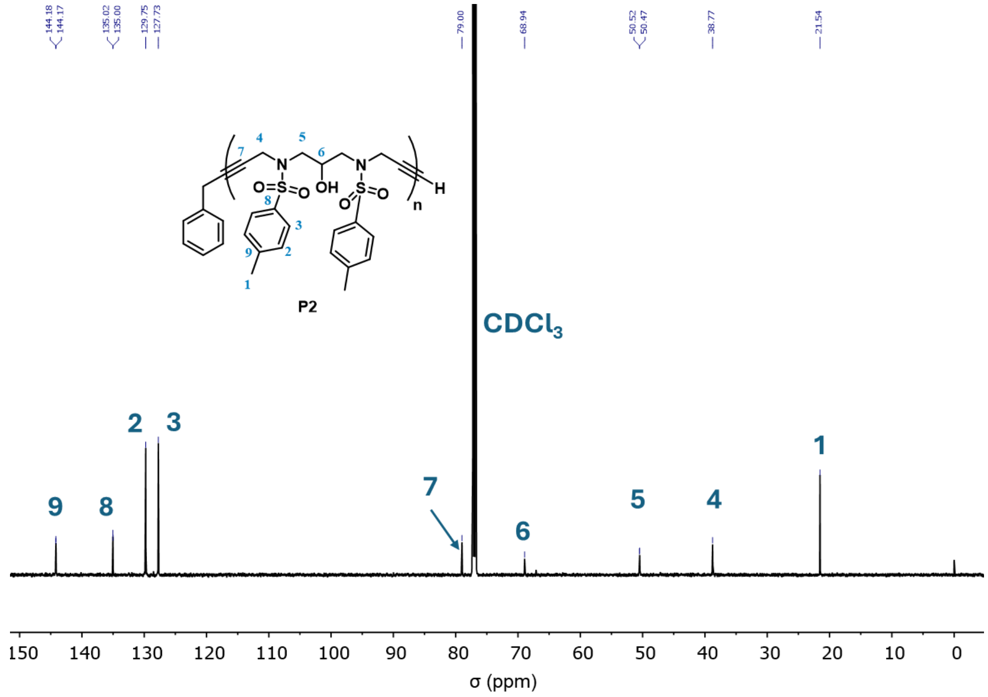

Figure S12. ^{1}H NMR, COSY NMR, HSQC NMR, and ^{13}C NMR spectra for **P2** [600 MHz, $CDCl_3$].

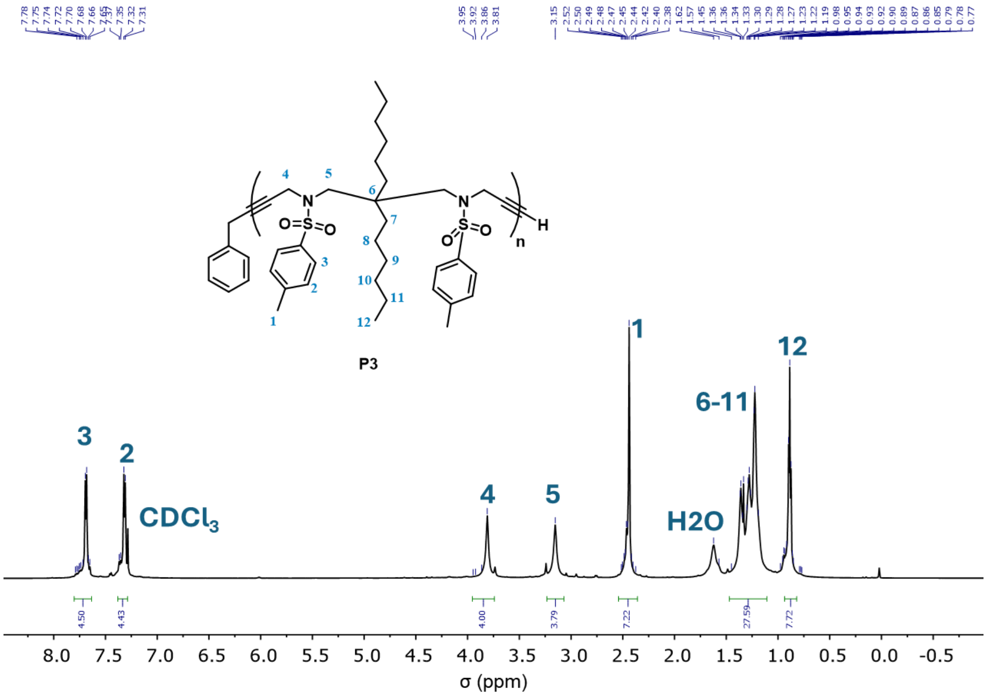
P3
3
2
CDCl3
4
5
1
H2O
6-11
12
σ (ppm)

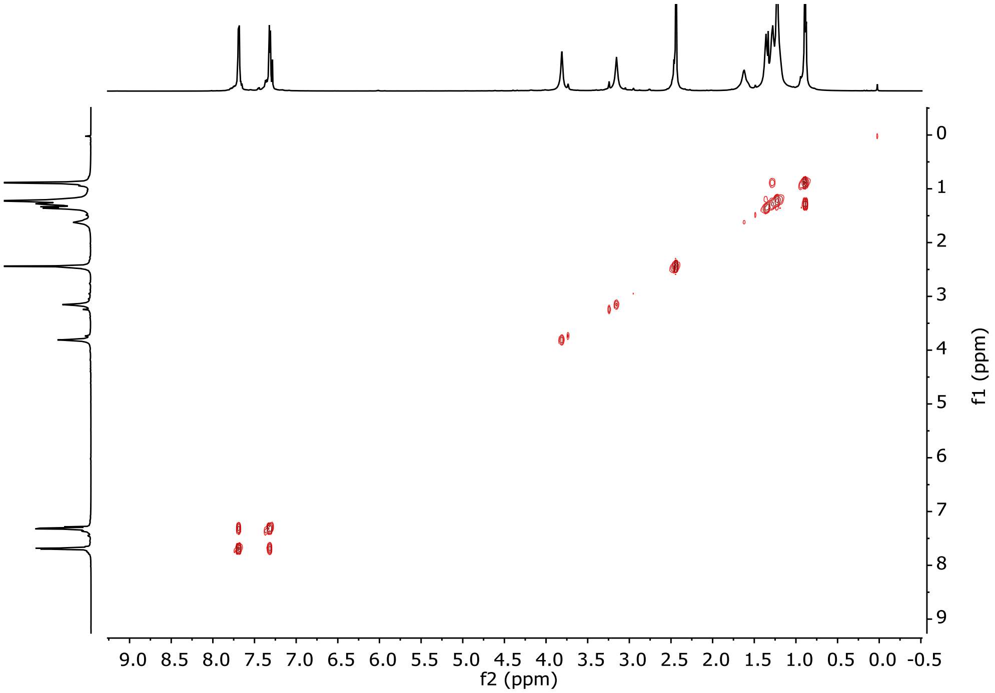
f1 (ppm)
f2 (ppm)

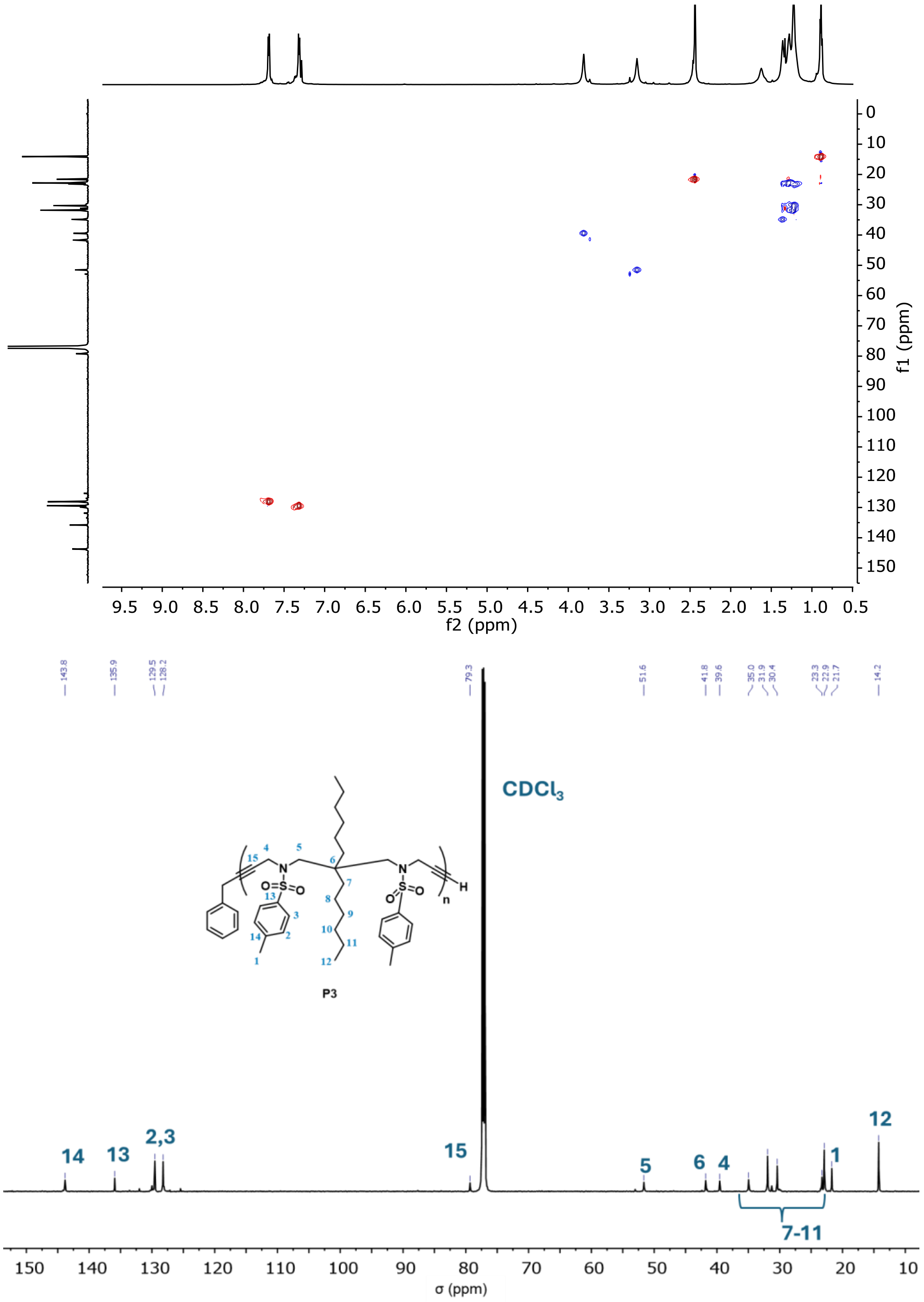

Figure S13. ^{1}H NMR, COSY NMR, HSQC NMR, and ^{13}C NMR spectra for **P3** [600 MHz, $CDCl_3$].

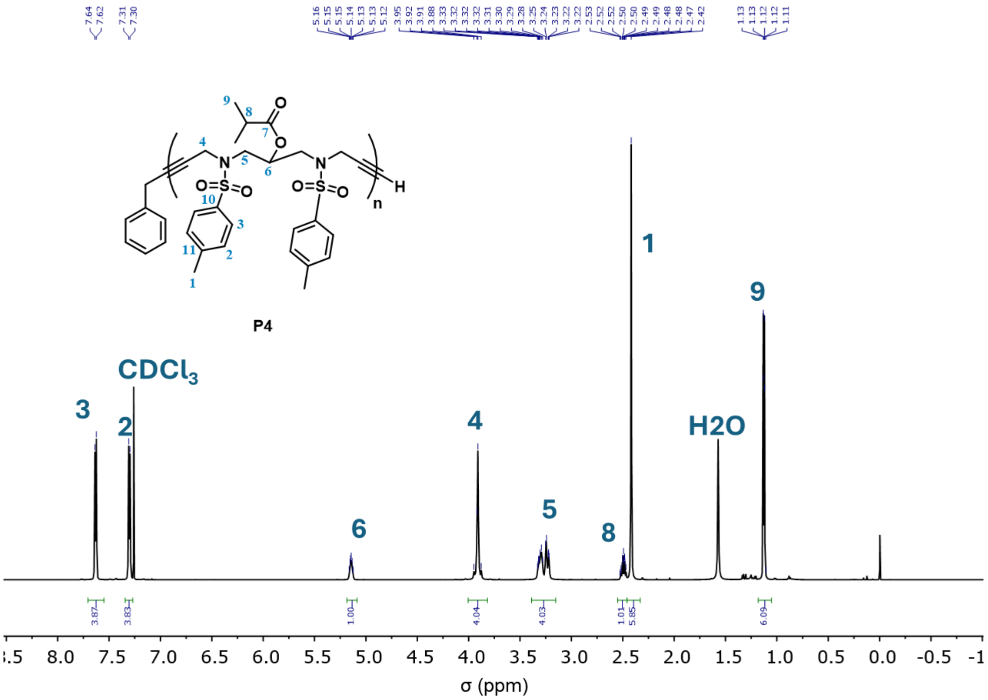
P4
CDCl$_3$
H2O
σ (ppm)

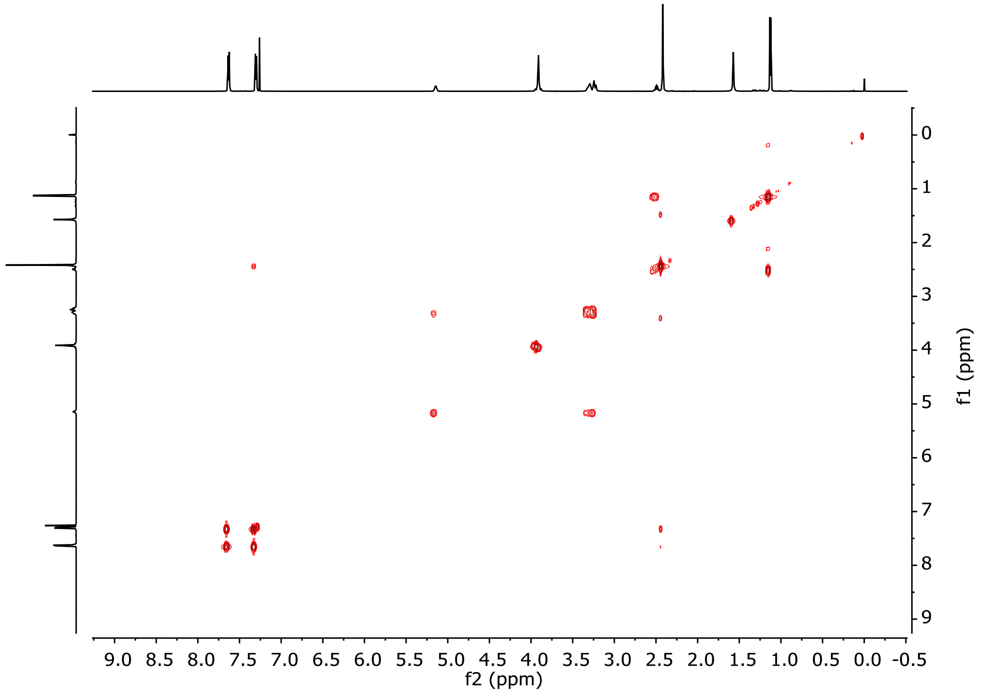
f2 (ppm)
f1 (ppm)

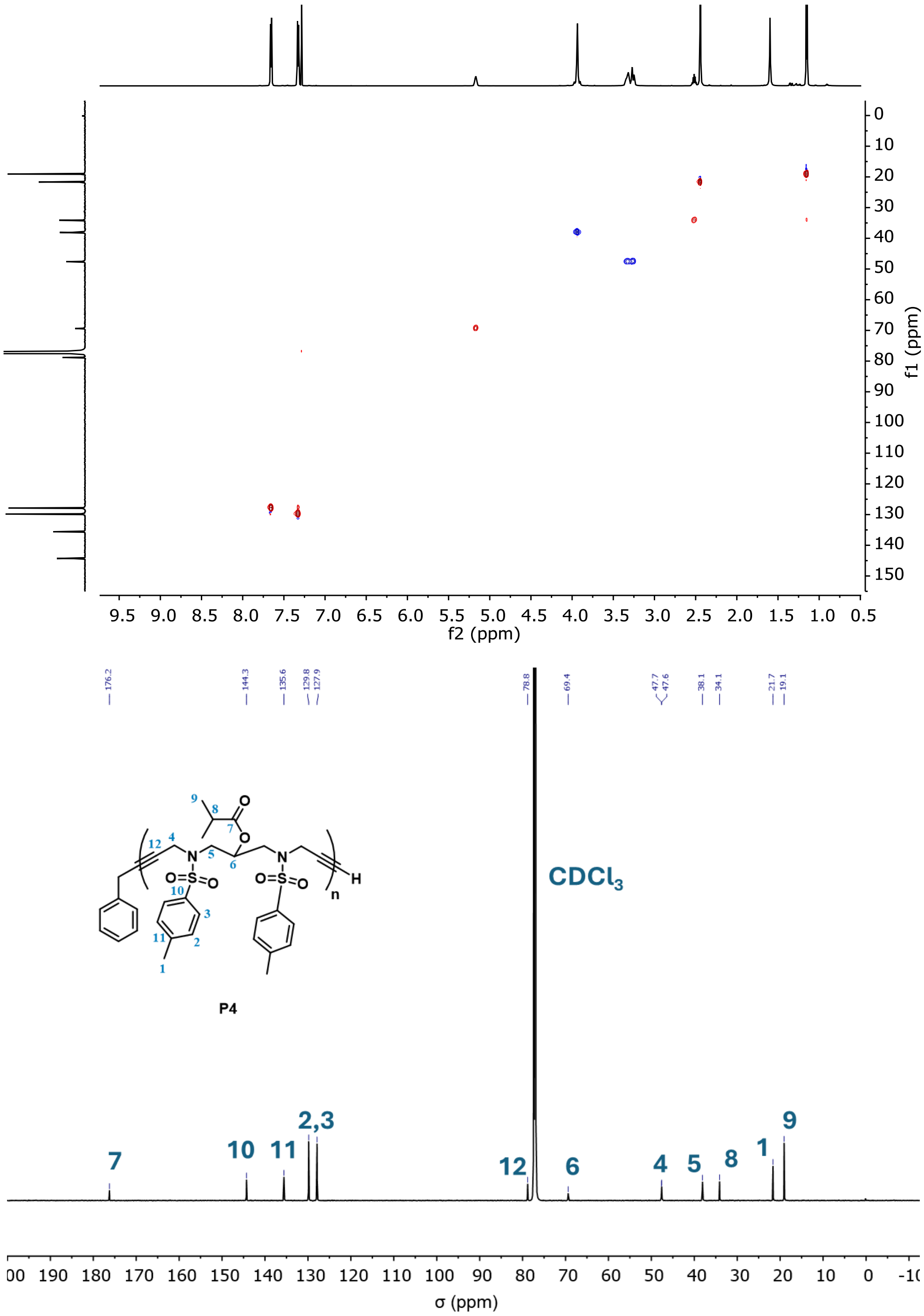

Figure S14. ^{1}H NMR, COSY NMR, HSQC NMR, and ^{13}C NMR spectra for **P4** [600 MHz, $CDCl_3$].

5. Initiation and Termination Study

5.1. Initiation Study

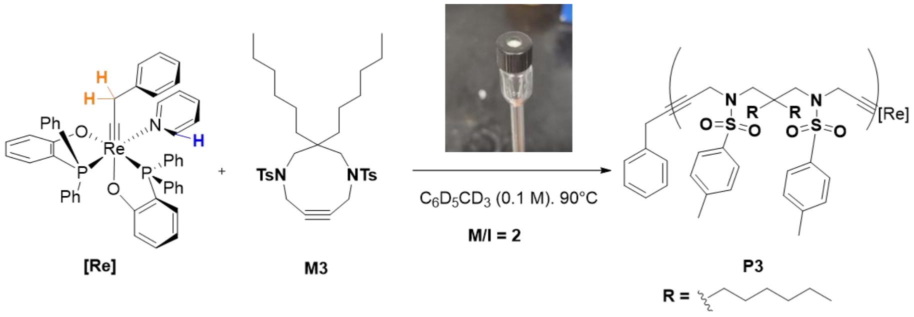

To determine the complete initiation timeframe of catalyst **[Re]** we studied the oligomerization of **M3** by ^{1}H NMR spectroscopy. The monomer was selected as it is soluble at room temperature in perdeuterated toluene.

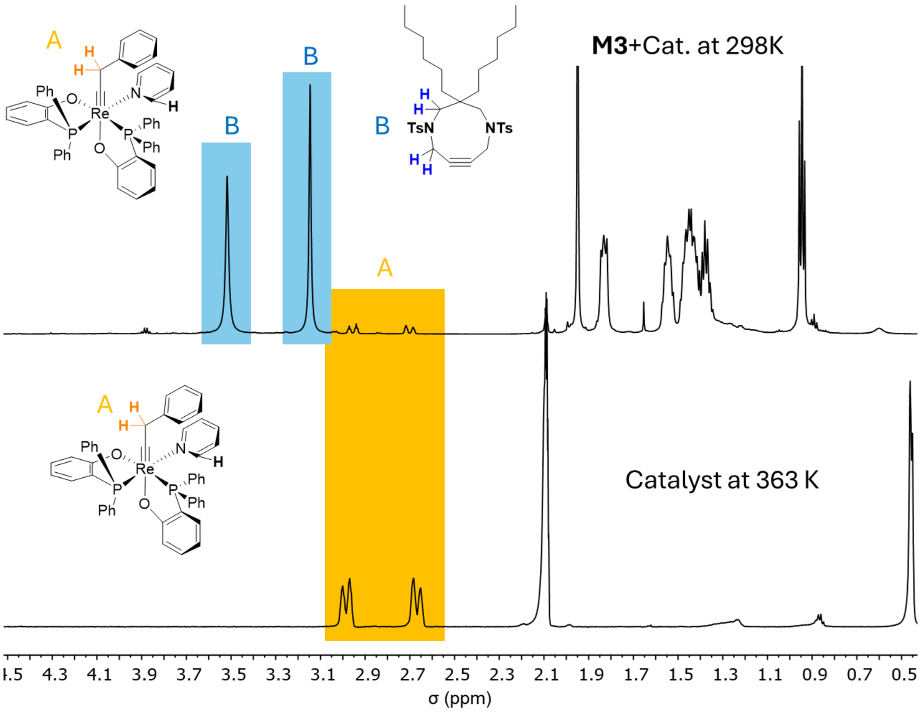

Figure S15. Stacked ^{1}H NMR (600 MHz, $C_6D_5CD_3$) of **M3** + **[Re] cat.** (top, 298K) and **[Re] cat.** (bottom, 363K).

A 4-mL sized screw-cap vial with septum was flame dried and charged with catalyst **[Re]** (9.7 mg, 10.48 μmol), monomer **M3** (12.6 mg, 20.97 μmol) and a magnetic bar. The vial was purged with

argon three times, and degassed toluene-d_8 (1.05 mL, 15 min argon sparging) was added. The mixture was stirred at room temperature for 5 min until completely homogenous. Then 0.5 mL of this stock solution were transferred to an over dried screw-capped NMR tube under argon. A room temperature (298 K) ^{1}H NMR of the mixture was recorded and compared with a ^{1}H NMR spectrum of catalyst **[Re]** at 90 °C (363 K) to ascertain the correspondence of the catalyst's significant proton signals at high temperature.

The sample was removed from the NMR spectroscope and the temperature stabilized at 363K. Then, the sample was injected in the NMR spectroscope and the time was recorded. No further locking or shimming was executed in order to minimize the time lag.

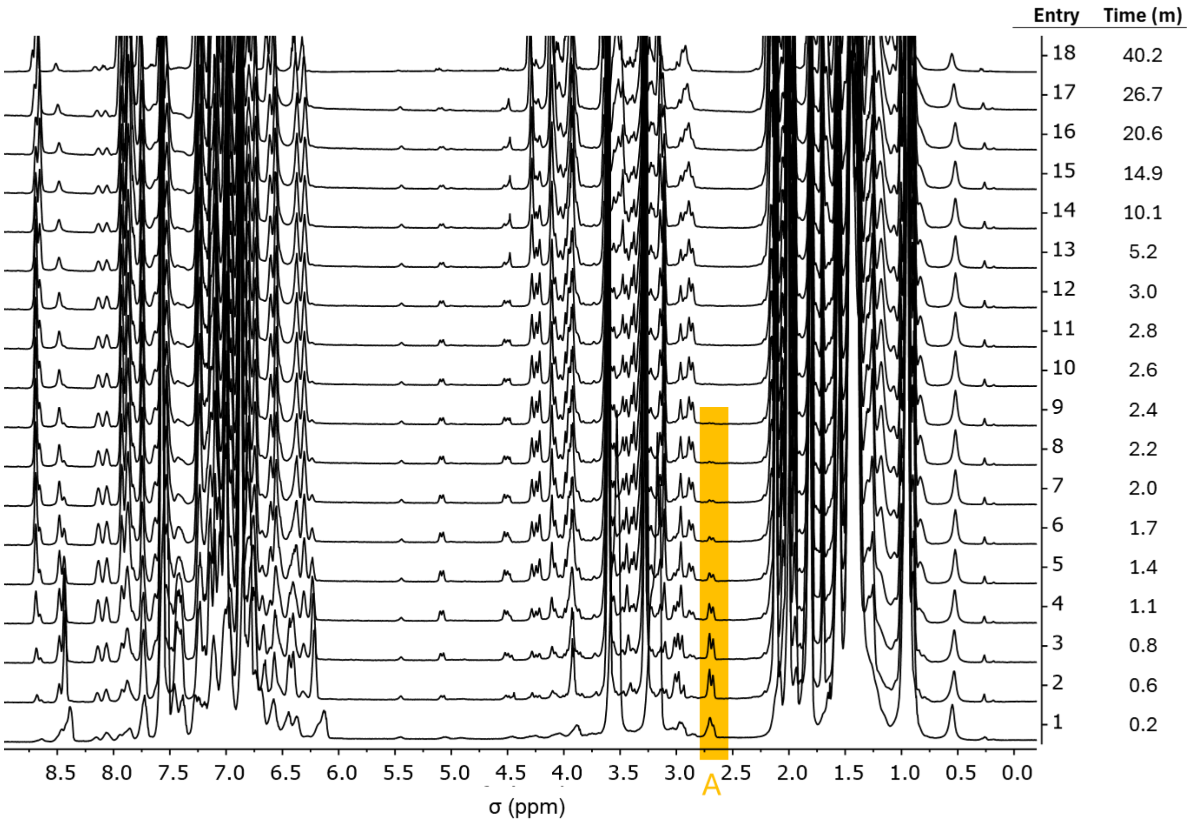

Figure S16. Stacked ^{1}H NMR (600 MHz, $C_6D_5CD_3$) of **M3** + cat. at 363K over time.

It can be observed from **Figure S16**, that the initial catalyst signal A gradually disappears between Entry 1 (0.2 min) and Entry 9 (2.4 min) and it is not observable at Entry 10 (2.6 min).

Additionally, we observed that at ca. 8.5 ppm multiple time-dependent pyridine species are present.

5.2. Termination Study

After the initial optimization of the polymerization of **M1** with catalyst **[Re]**, we investigated the fate of the chain-end. Initially, we run an M/I = 40 polymerization of **M1**, precipitated the polymer and analyzed the polymer and the filtrate by ^{1}H and ^{31}P NMR. By ^{1}H NMR of **P1** it is possible to observe small aromatic signals deriving from the chain-head in the 6.5-7.9 ppm area, while the filtrate ^{1}H NMR shows several aromatic signals, likely belonging compounds with ligands deriving from the initial catalyst (**Figure S17**).

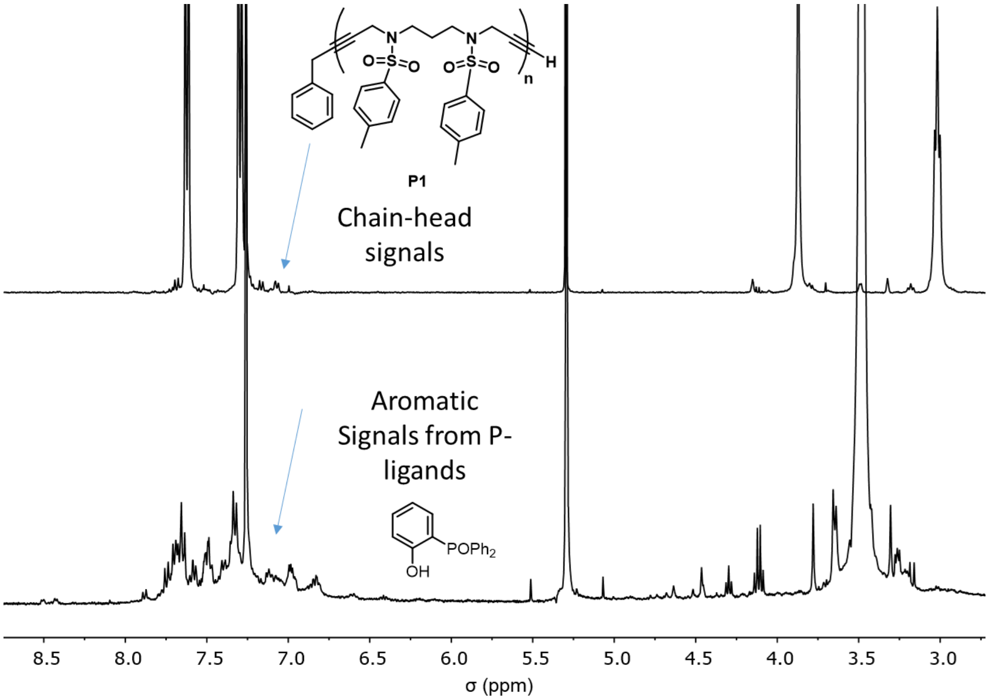

Figure S17. ^{1}H NMR stacked spectra of polymer **P1** (top) and the polymerization filtrate (bottom) [400 MHz, $CDCl_3$].

Indeed, ^{31}P NMR of **P1** does not show any signal of phosphorous containing moiety, suggesting that after reaction the ligands majorly dissociate from the [Re]. Whereas the ^{31}P NMR spectrum of the filtrate shows one clear signal at 39.7 ppm, which corresponds to the reported ^{31}P NMR of (2-hydroxyphenyl)diphenylphosphine oxide (**Figure S18**).[10] For non-oxidized PO ligand, the chemical shift is –28.62 ppm**[11]**

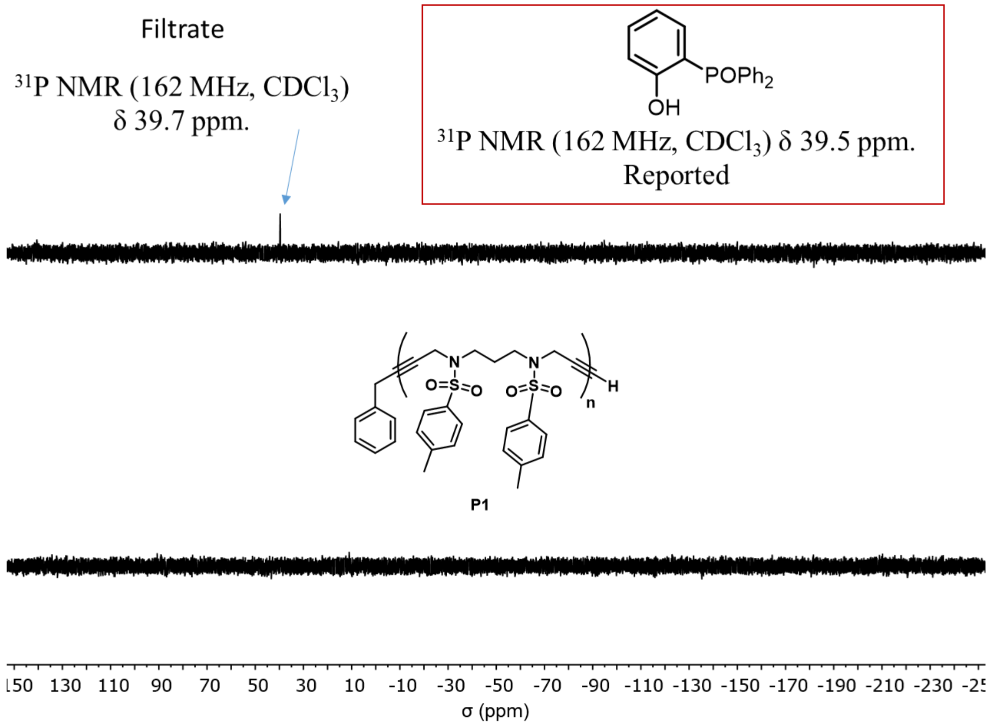

Figure S18. ^{31}P NMR stacked spectra of the polymerization filtrate (top) and polymer **P1** (bottom) [162 MHz, $CDCl_3$].

To identify the ^{1}H NMR signals of the benzyl chain-head, we run an M/I = 5 polymerization of **M1** under standard conditions without adding any quencher (crude NMR). As expected of small oligomers, the signals of **P1** coalesce less, but the signals of the benzyl chain-head could be identified and assigned by 2D NMR analysis (**Figure S19**). Additionally, we measured a 4.9:1 ratio between the chain-end and the repeat unit, which approached the intended M/I = 5.

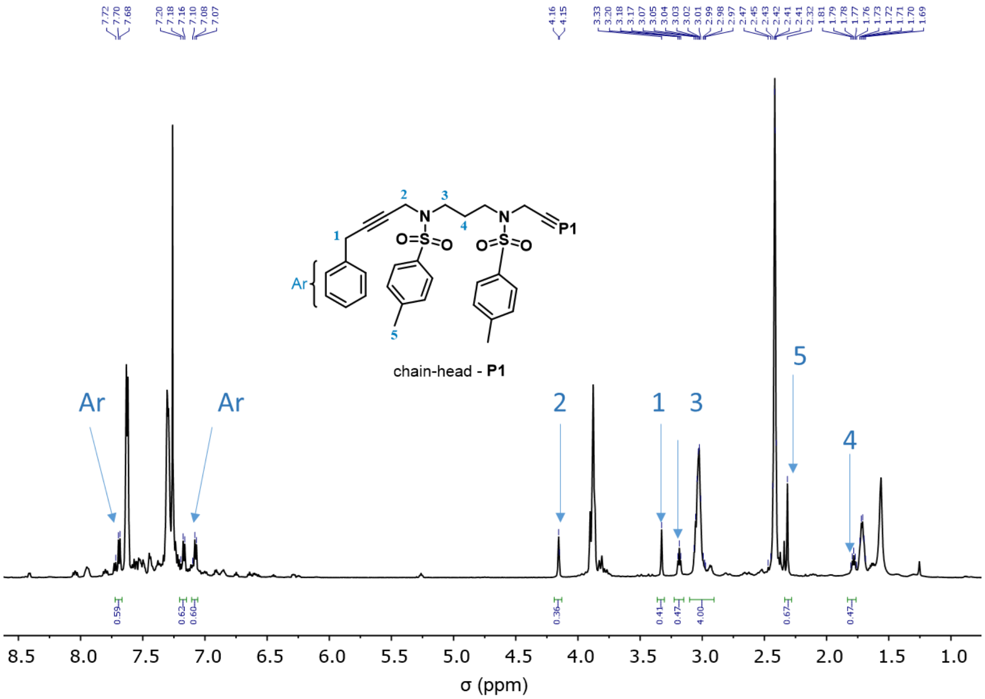
chain-head - P1
Ar
2
1
3
5
4
σ (ppm)

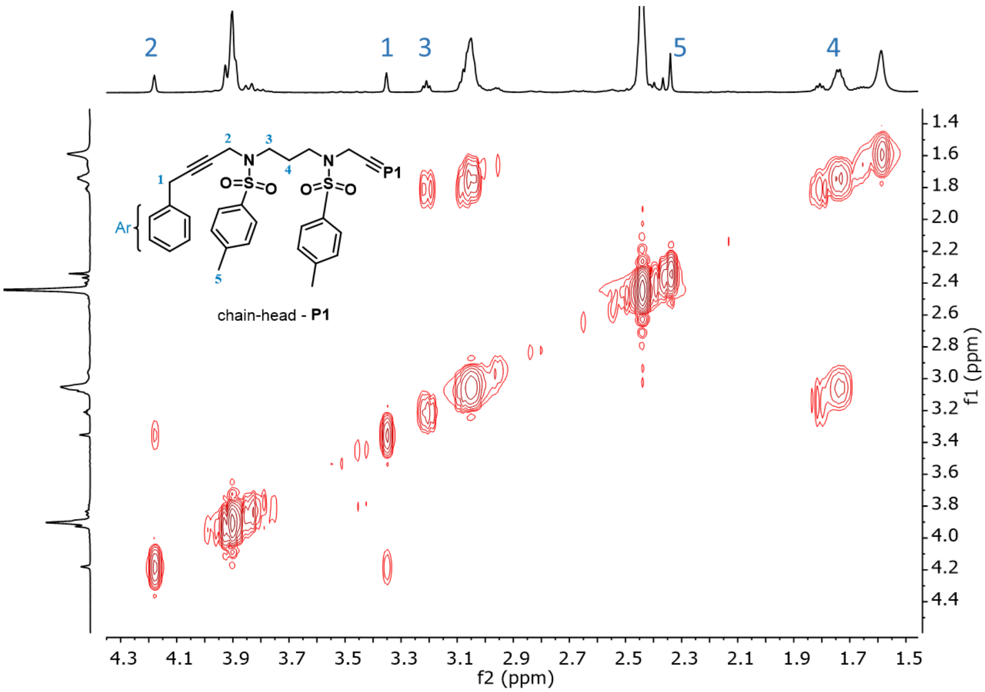
chain-head - P1
f2 (ppm)
f1 (ppm)

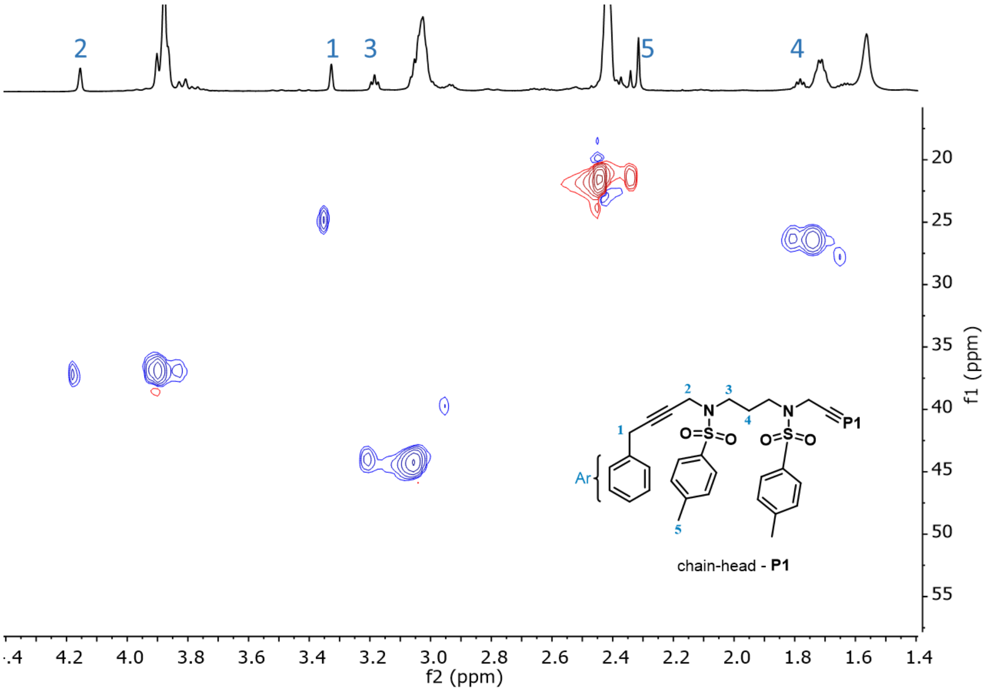

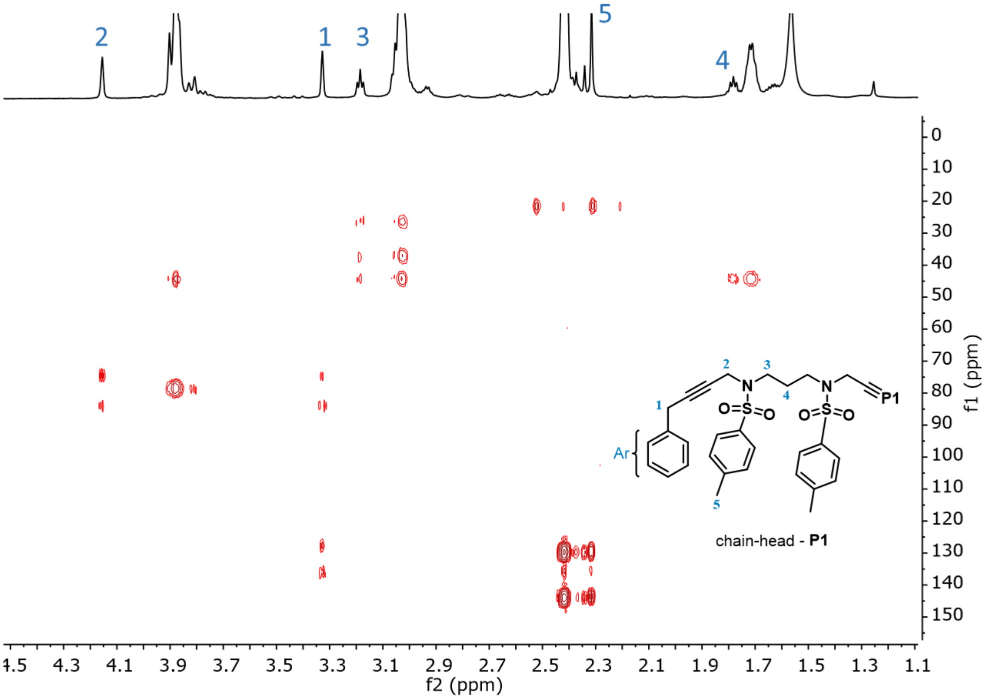

Figure S19. ^{1}H NMR, COSY NMR, HSQC NMR, and HMBC NMR spectra for M/I = 5 **P1** [600 MHz, $CDCl_3$].

Then we repeated the same polymerization. Upon completion (1 h) an aliquot was removed, and 1-(tert-butyl)-4-ethynylbenzenewas added. This was followed by an obvious color change from yellow to brown. The polymer was isolated and analyzed by NMR spectroscopy.

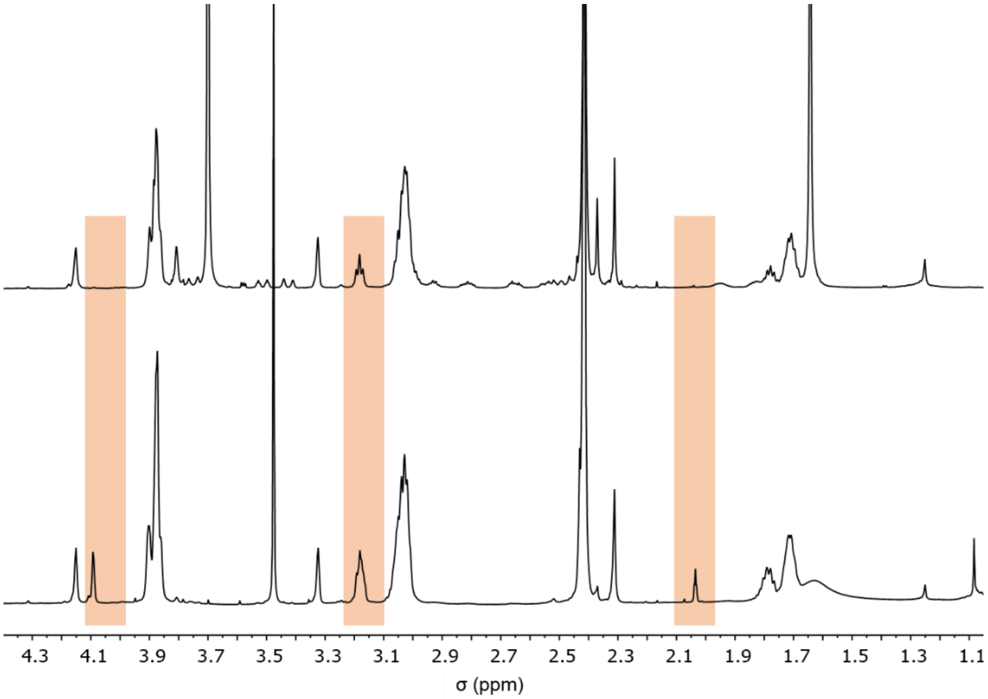

Figure S20. ^{1}H NMR stacked spectra of polymer **P1** before quenching (top) and after quenching filtrate (bottom) [400 MHz, $CDCl_3$].

As can be appreciated from **Figure S20**, before termination the benzylic chain-head signals are well defined and visible, while we could not locate the tail signals. After quenching, a new propargylic signal is located at ca. 4.1 ppm (orange area). Similarly, at ca. 3.2 ppm the alpha-heteroatom protons coalesce in one signal, with corresponding increase in integration, while an acetylenic proton is observable at ca. 2 ppm.

The signals present in the ^{1}H NMR (**Figure S20**) could be assigned using 2D NMR analysis and they corresponded to complete transfer of the acetylenic carbon of the terminating reagent (**Figure S21**).

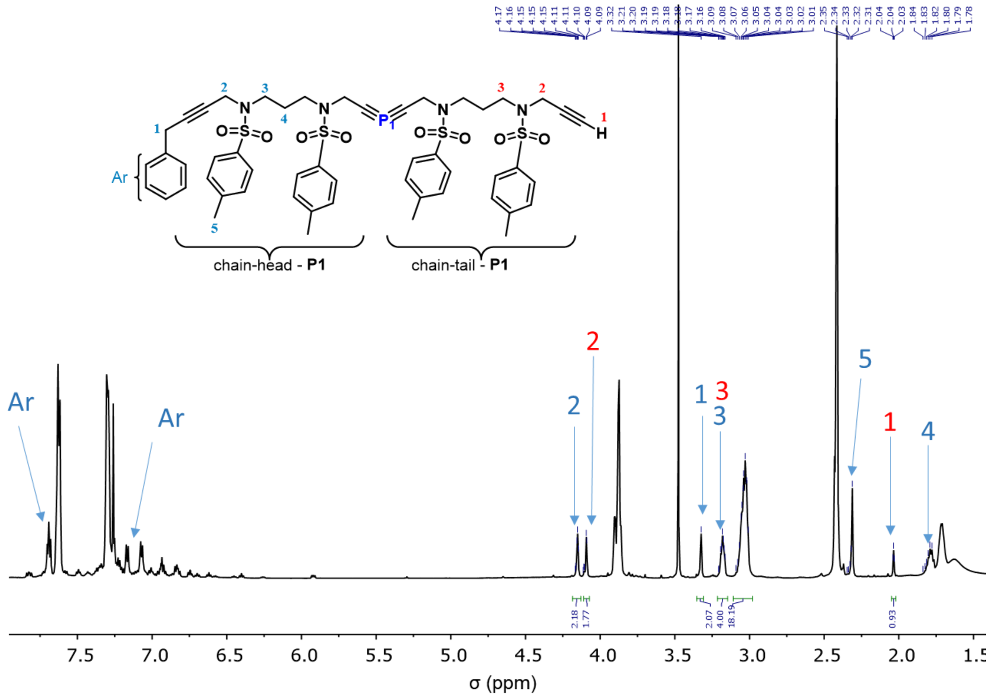
chain-head - P1
chain-tail - P1
Ar
σ (ppm)

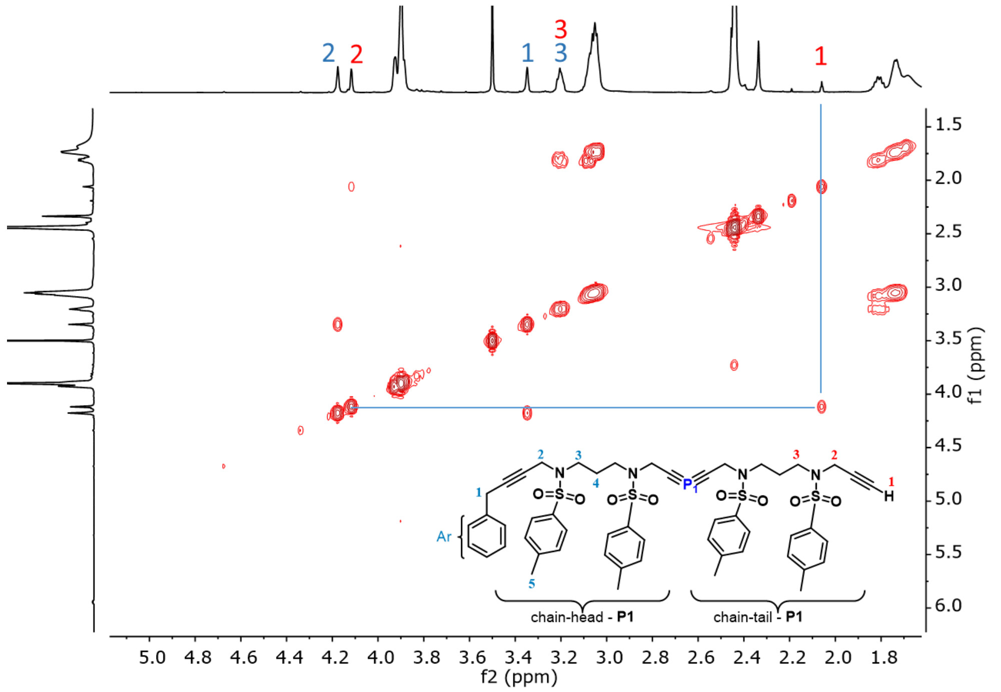
chain-head - P1
chain-tail - P1
Ar
f2 (ppm)
f1 (ppm)

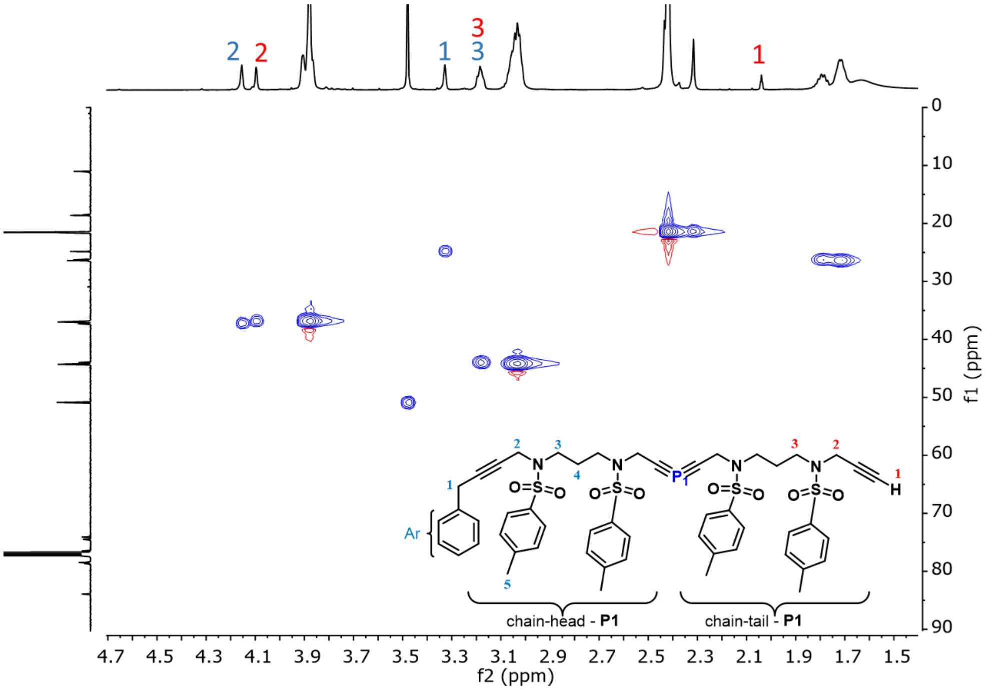
2
2
1
3
3
1
0
10
20
30
40
50
60
70
80
90
f1 (ppm)
4.7 4.5 4.3 4.1 3.9 3.7 3.5 3.3 3.1 2.9 2.7 2.5 2.3 2.1 1.9 1.7 1.5
f2 (ppm)
Ar
P1
H
chain-head - P1
chain-tail - P1

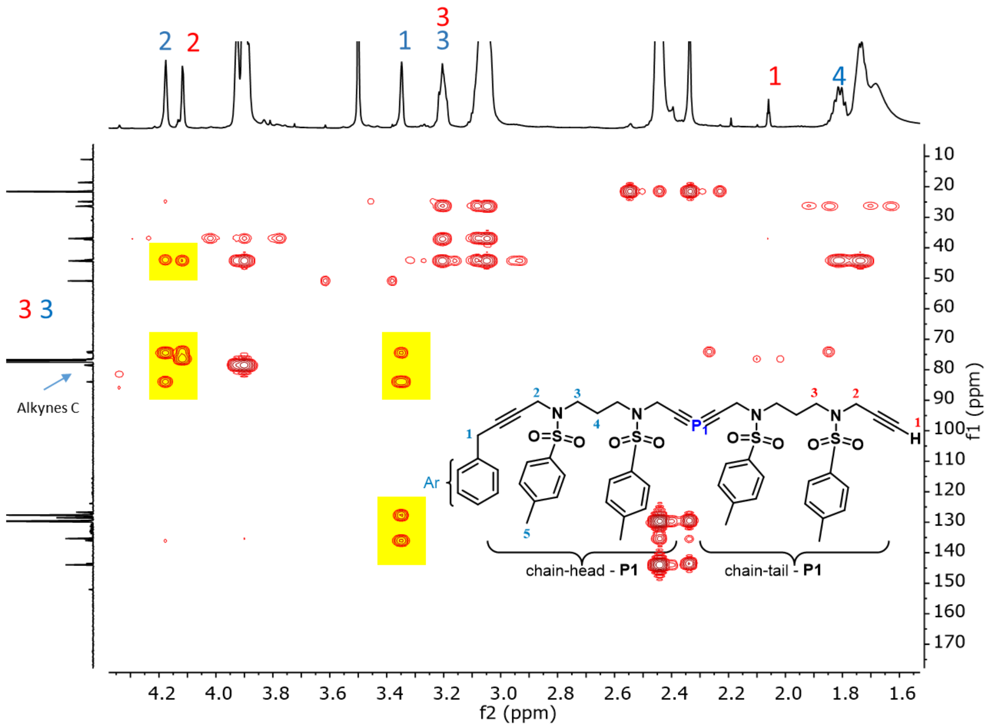
2
2
1
3
3
1
4
3 3
Alkynes C
10
20
30
40
50
60
70
80
90
100
110
120
130
140
150
160
170
f1 (ppm)
4.2 4.0 3.8 3.6 3.4 3.2 3.0 2.8 2.6 2.4 2.2 2.0 1.8 1.6
f2 (ppm)
Ar
P1
H
chain-head - P1
chain-tail - P1

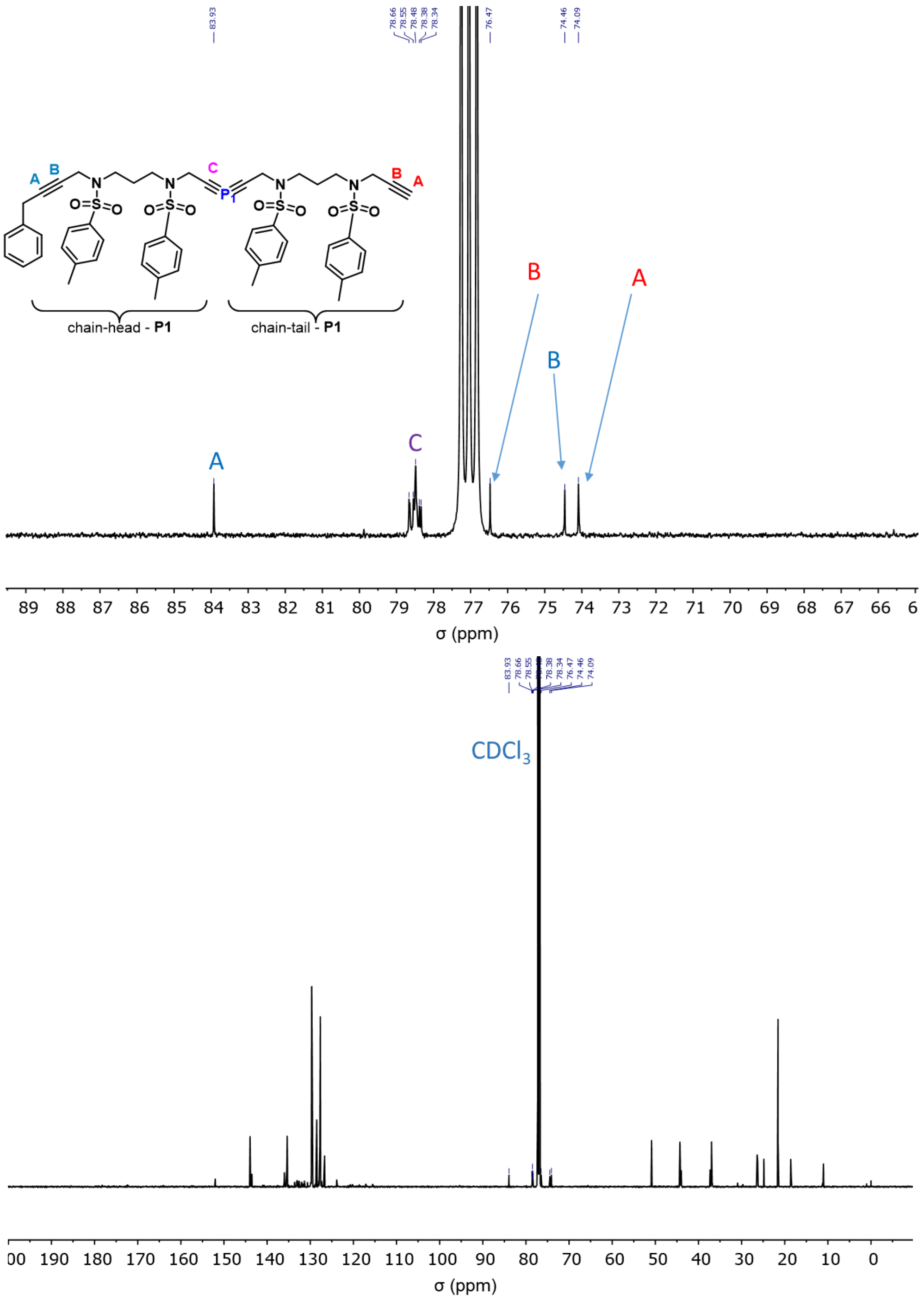

Figure S21. From top to bottom: ^{1}H NMR, COSY NMR, HSQC NMR, HMBC NMR, zoom in of the alkyne area of the ^{13}C NMR and ^{13}C NMR spectra for M/I = 5 **P1** [600 MHz, $CDCl_3$].

6. DSC Analysis

P1, **P2**, and **P4** with M_ns of 258.9, 118.7, and 235.2 kDa, respectively, were characterized with DSC. **P3** was excluded due to the relatively low M_n compared to the other samples. The results are summarized in **Table S1** and the corresponding DSC curves are shown in **Figures S22 – S24**. DSC was conducted using the following general method: segment gas N_2, 50 ml/min; 1) Isotherm: 10 min at -20 °C; 2) temp. ramp up: -20 °C to 120 °C, heating rate of 10 °C/min.

Table S1. Summary of T_g and T_m values.

polymer	T_g (˚C)^A	T_g (˚C)^B
**P1**	16.7	76.0
**P2**	8.7	79.8
**P4**	16.8	79.5

$^{A)}$ Onset values using the glass transition measurement tool in the Mettler Toledo DSC software.

$^{B)}$ Values from the peak measurement tool in the Mettler Toledo DSC software

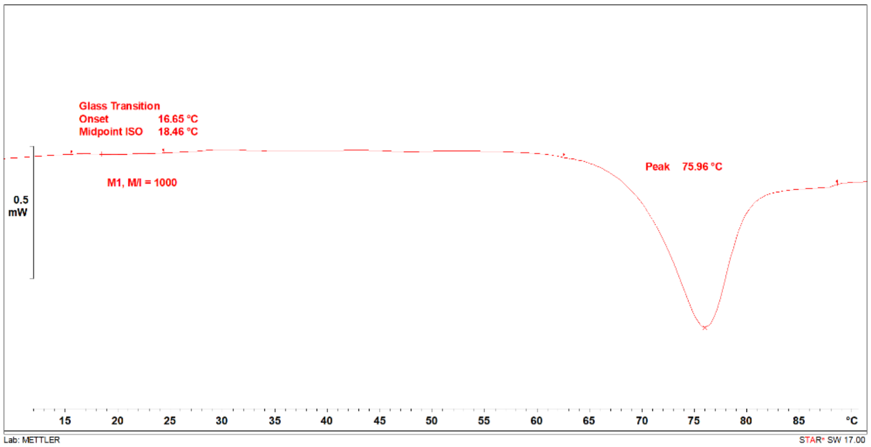

Figure S22. DSC traces for **P1** (258.9 kDa).

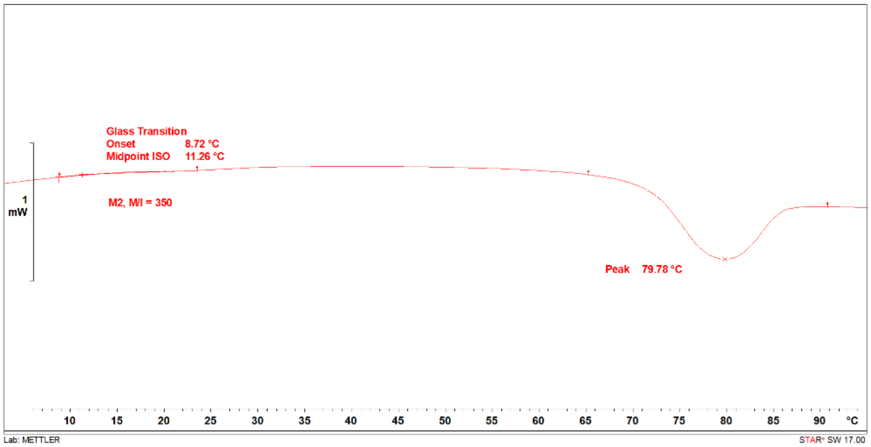

Figure S23. DSC traces for **P2** (118.7 kDa).

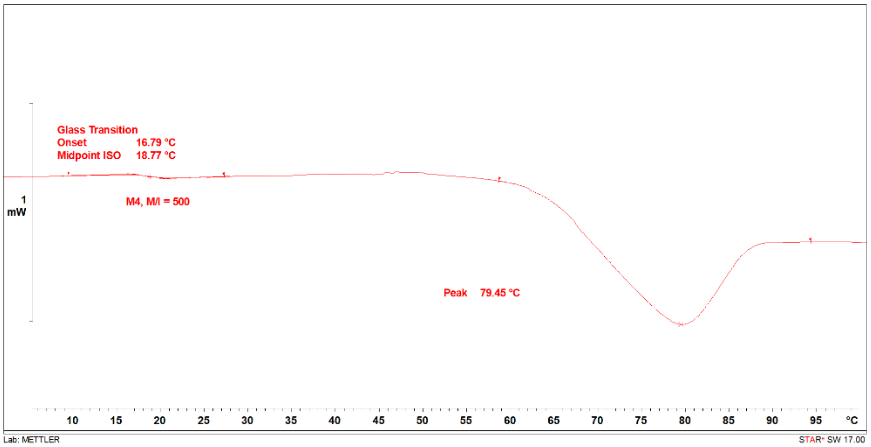

Figure S24. DSC traces for **P4** (235.2 kDa).

7. Hydrolysis of P4 to P2

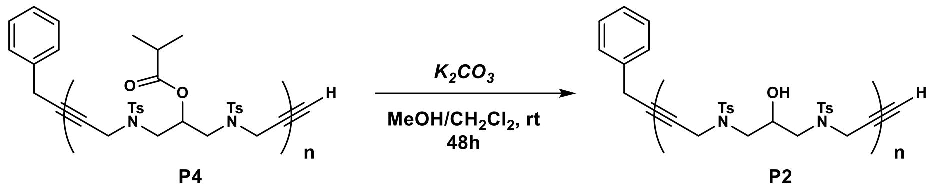

A 5-mL sized screw-cap tube was charged with **P4** (6.5 mg), K_2CO_3 (26.6 mg, ca. 10 equivalents), CH_2Cl_2/MeOH (0.5 mL, 2/1) and a magnetic bar. The mixture was vigorously stirred at room temperature for 48 hours. Then, the reaction was diluted with 1 mL of CH_2Cl_2, filtered on a pad of celite and the solvent removed under reduced pressure. The residue was dissolved in 2 mL of CH_2Cl_2, dried over Na_2SO_4 and the solvent removed under reduced pressure. Polymer P2 was isolated as a grey powder in 83% yield (5.4 mg). The measured DP = 19 by ^1H NMR integration of the chain ends (DP of **P4** = 24), which was within the margin of error of the measurement.

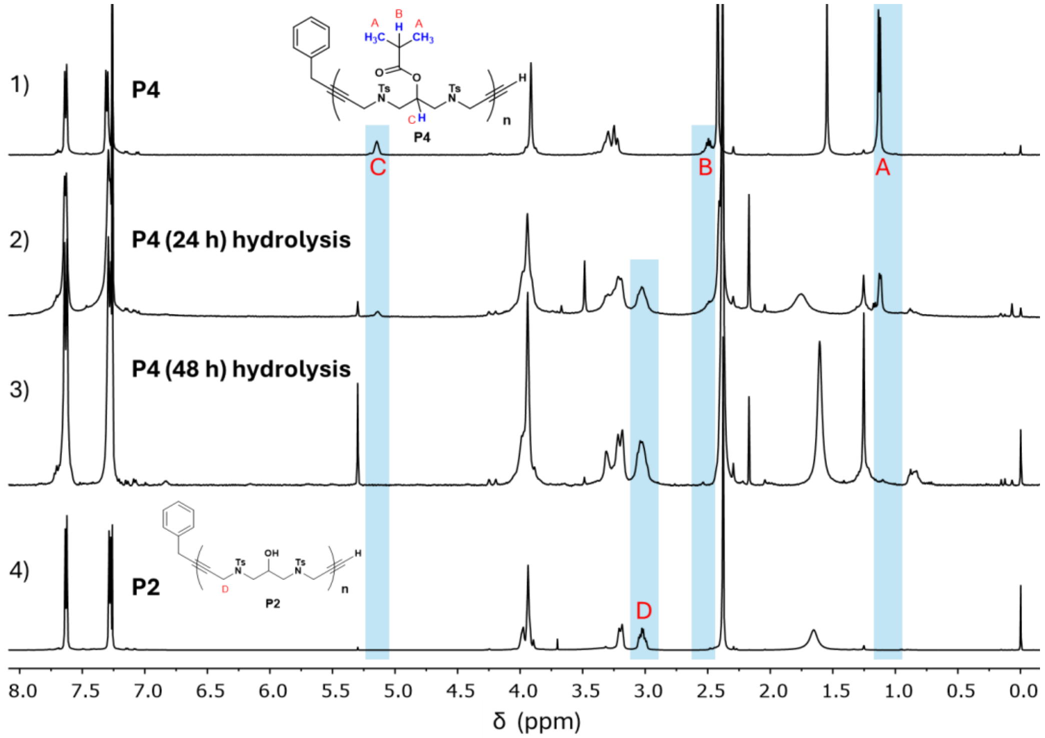

Figure S25. ^1H NMR stacked spectra of polymer **P4** (1), **P4** after 24 hours hydrolysis (2), **P2** isolated after 48 hours hydrolysis (3) and **P2** prepared by polymerization of **M2** (4) [500 MHz, $CDCl_3$].

8. Additional Figures

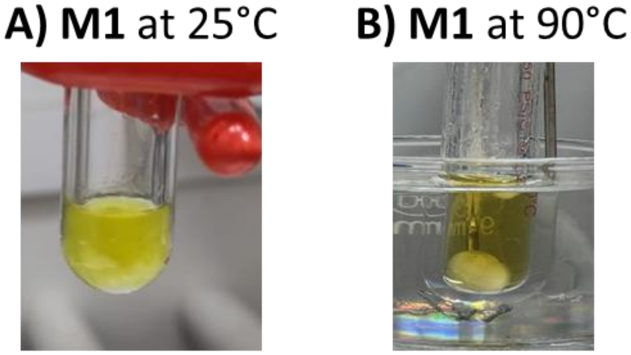

Figure S26. Solubility of **M1** at A) room temperature and B) reaction temperature.

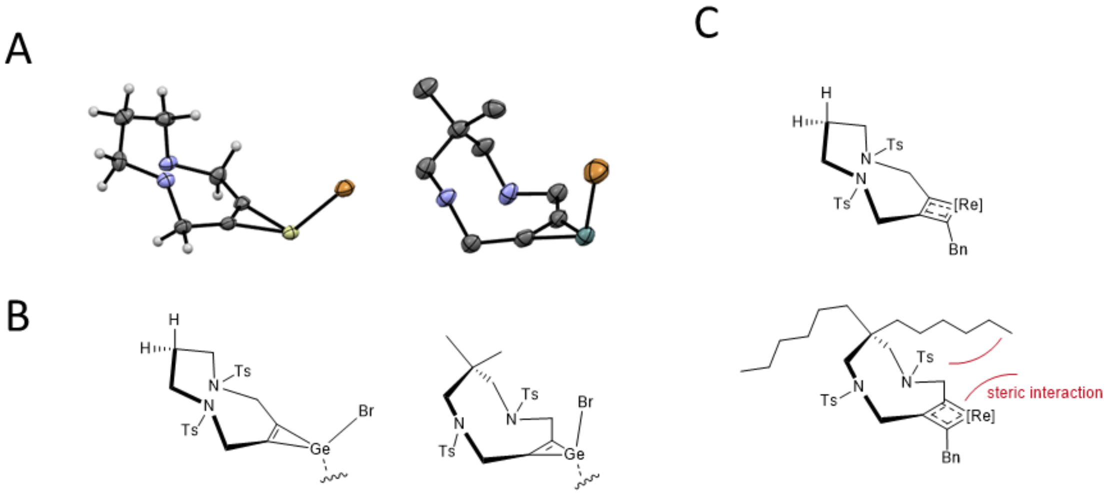

Figure S27. A) ORTEPs of Germanium adducts derived from **M1** and B) geminal dimethyl analogue of **M3** (ORTEP, thermal ellipsoids set at 50% probability, parts of the structure were omitted for clarity, CCDC codes 1907439 - 1907435).[12] B) Renditions of structures in A). C) Proposed difference in transition states conformations for the **[Re]** adducts of **M1** and **M3**.

A reason for the poor reactivity of **M3** might be found in the conformational change of the cyclononayne in respect to **M1**. The X-ray structure of **M1** has been reported[1] but unfortunately not that of a geminal dialkyl analogues. Fortunately, Germanium adducts X-ray structures were recorded and show a stark difference in the cyclononayne conformation. It seems that a geminal substitution points one of the aliphatic chains towards the reactive site. Therefore, it could be

plausible that the conformational difference and the increased shielding of the alkyne in **M3** lead to unsatisfactory reactivity.

Entry	Monomer	M/I	Temp. (°C)	Conv (%)[a]	Time (h)	^{1}H NMR Degree of Polymerization[b]	M_n (kDa)[c]	Đ[c]
1	M1	20	90	>99	2	22	6.7	1.13
2	M2	20	90	>99	2	23	8.3	1.20
3	M3	20	90	>99	2	19	8.7	1.13
4	M4	20	90	>99	2	24	9.1	1.19

[a] Determined by ^{1}H NMR analysis of the crude mixture. [b] Determined by integrating the ^{1}H NMR signals of the repeat unit and the chain-end. [c] Determined by THF size exclusion chromatography calibrated by polystyrene standards.

Figure S28. Tabulation of the degree of polymerization obtained by integrating ^{1}H NMR signals of the repeat unit and the chain-end for M/I = 20 polymers.

9. NMR Spectra

9.1 New Compounds NMR Data

^{1}H NMR of S9 (500 MHz, $CDCl_3$)

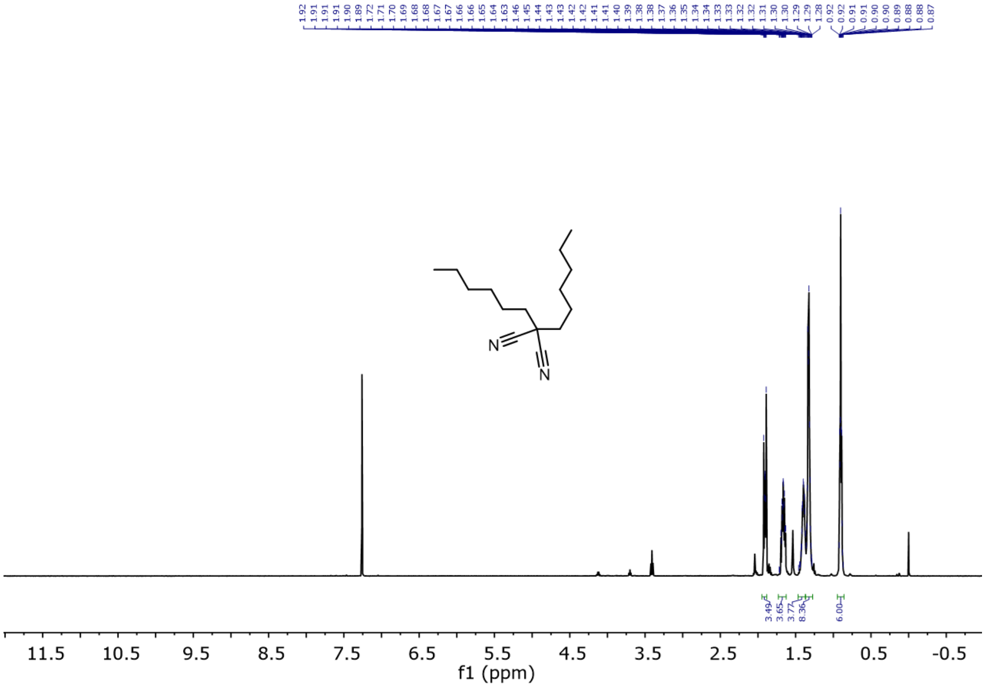

^{13}C NMR of S9 (101 MHz, $CDCl_3$)

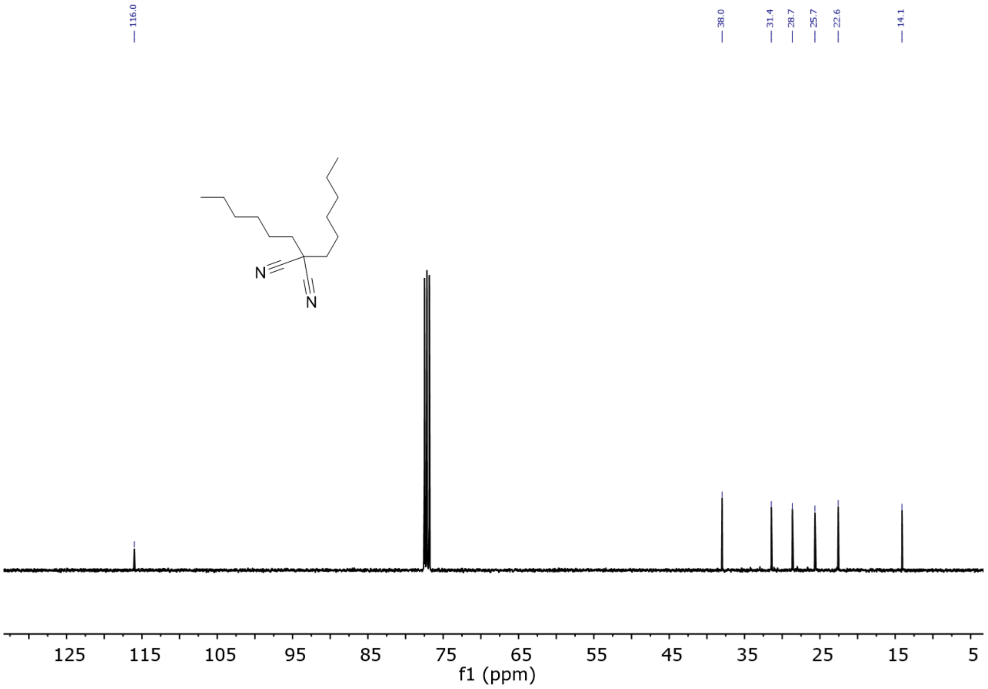

^{1}H NMR of S10 (600 MHz, CD_3OD)

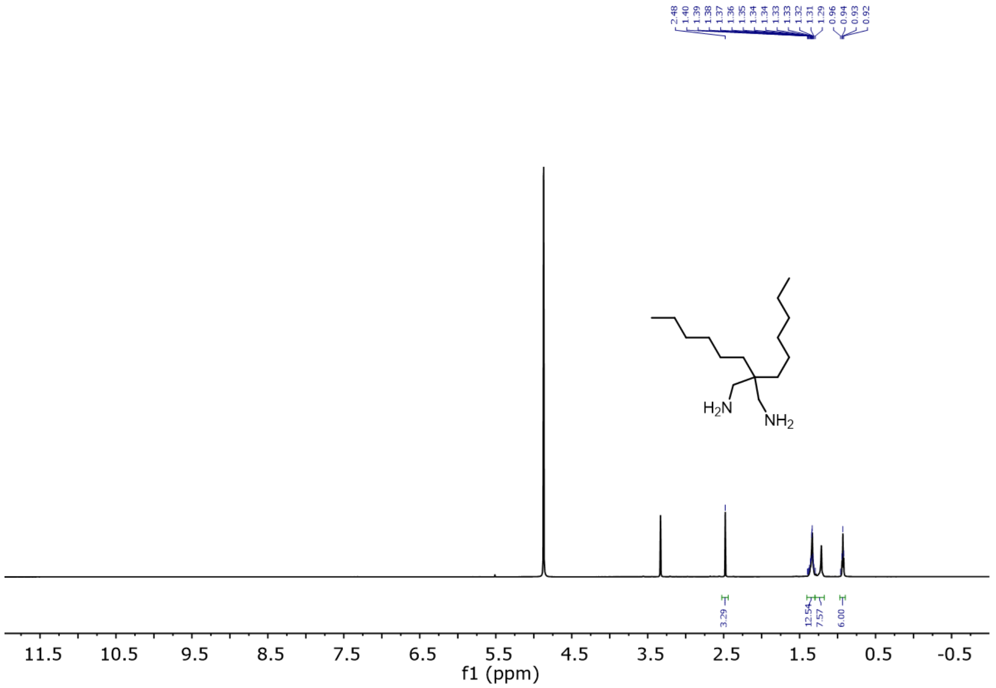

^{13}C NMR of S10 (126 MHz, CD_3OD)

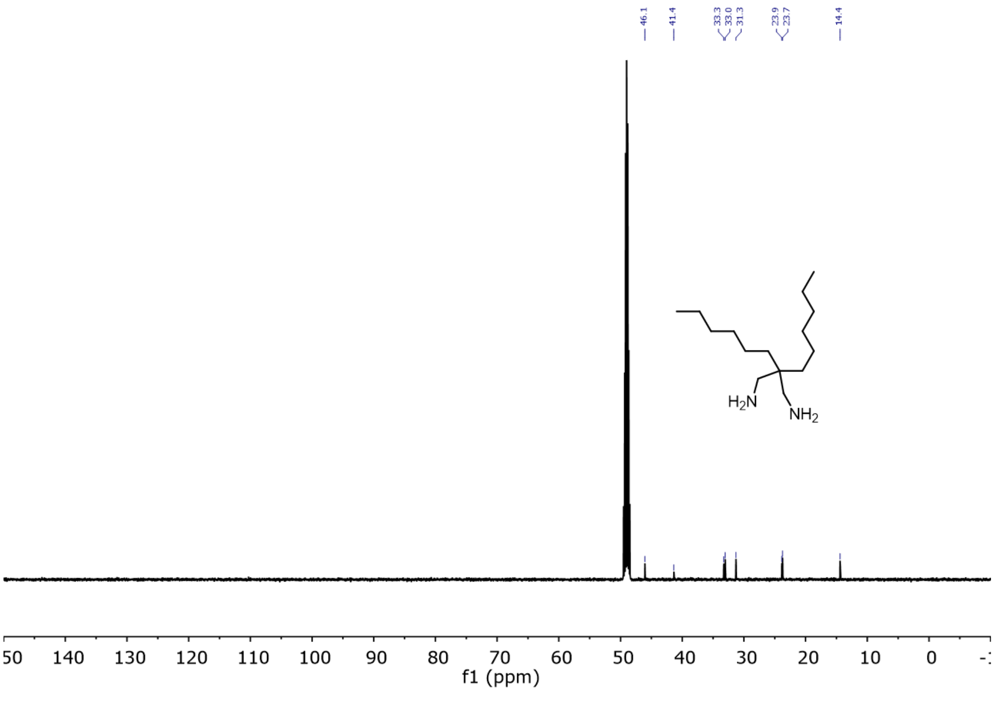

^{1}H NMR of S11 (500 MHz, $CDCl_3$)

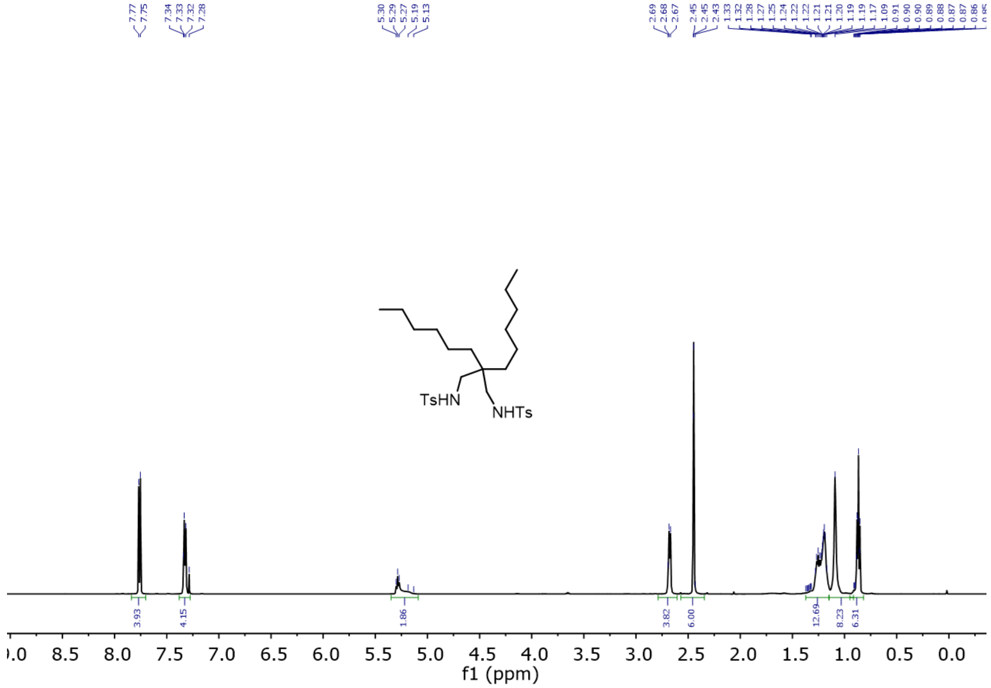

^{13}C NMR of S11 (101 MHz, $CDCl_3$)

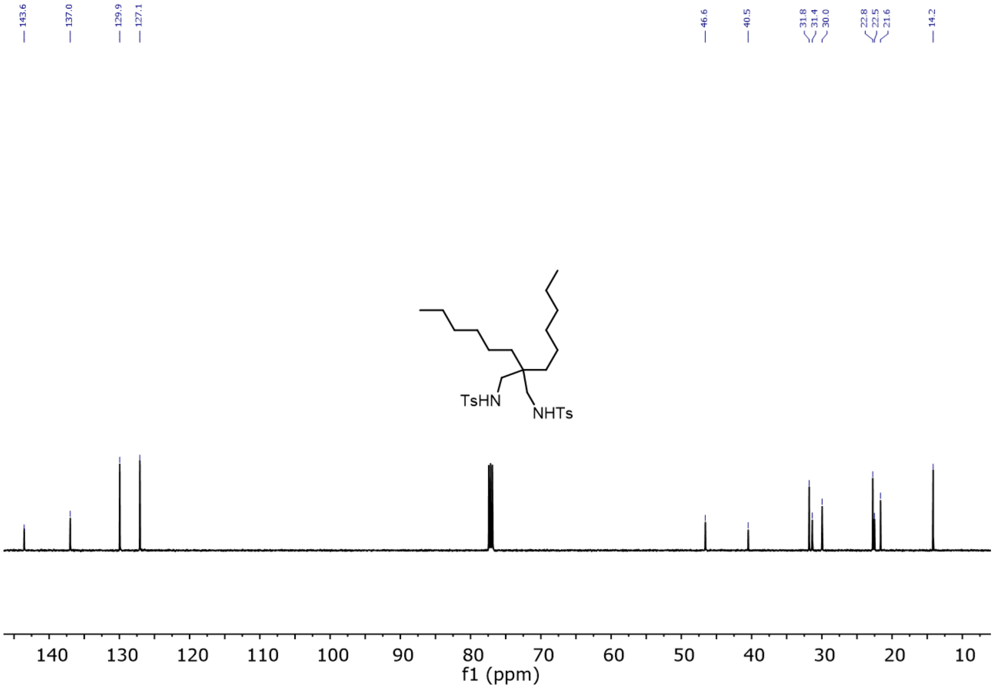

^{1}H NMR of M3 (600 MHz, $CDCl_3$)

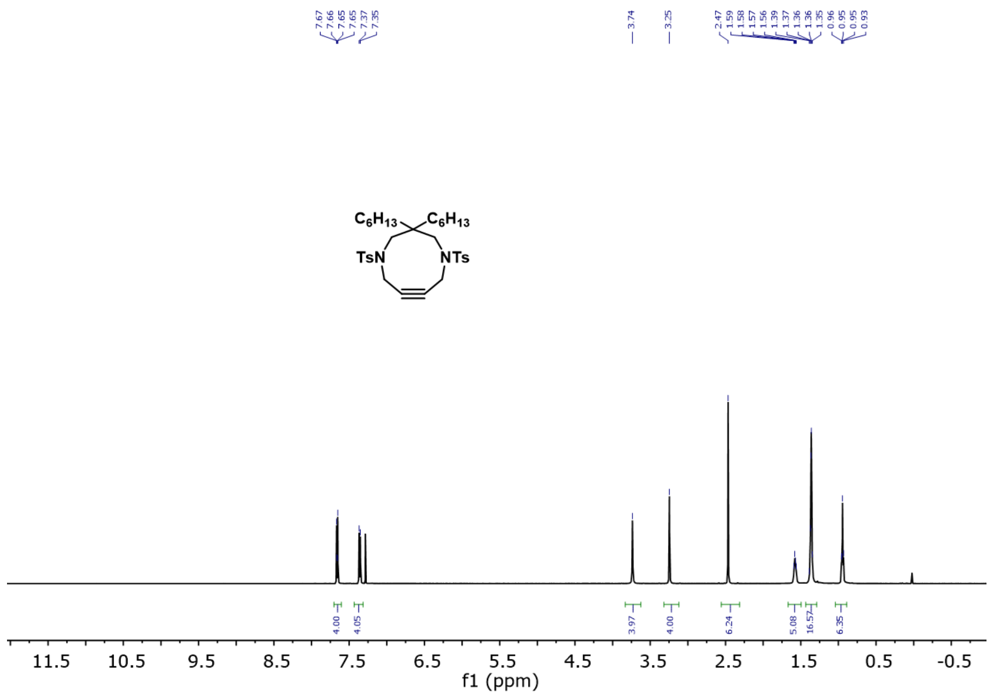

^{13}C NMR of M3 (126MHz, $CDCl_3$)

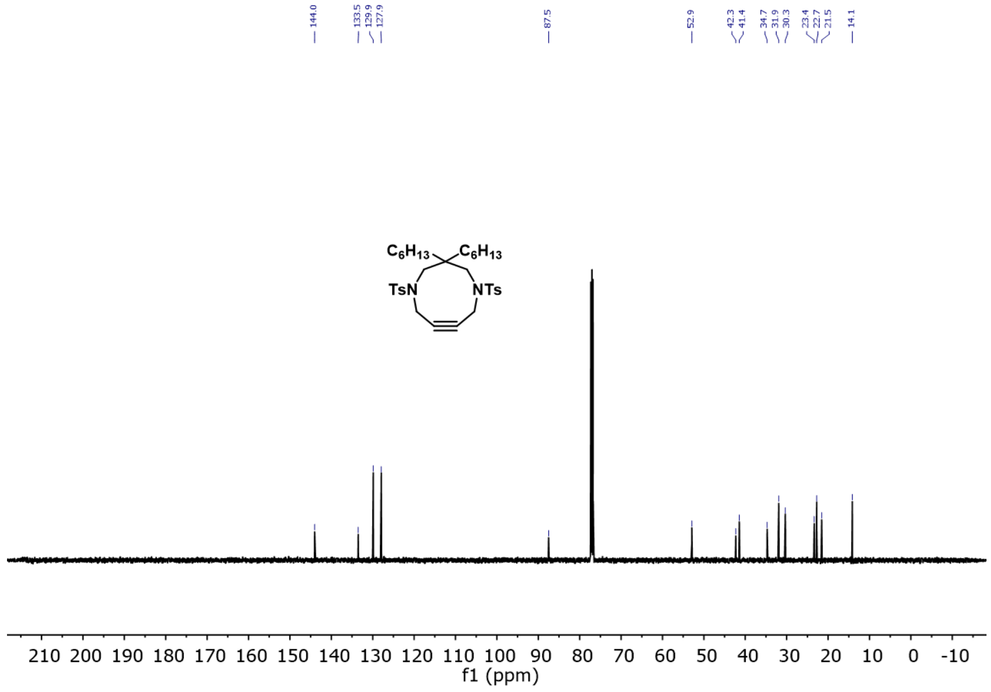

^{1}H NMR of M4 (500 MHz, $CDCl_3$)

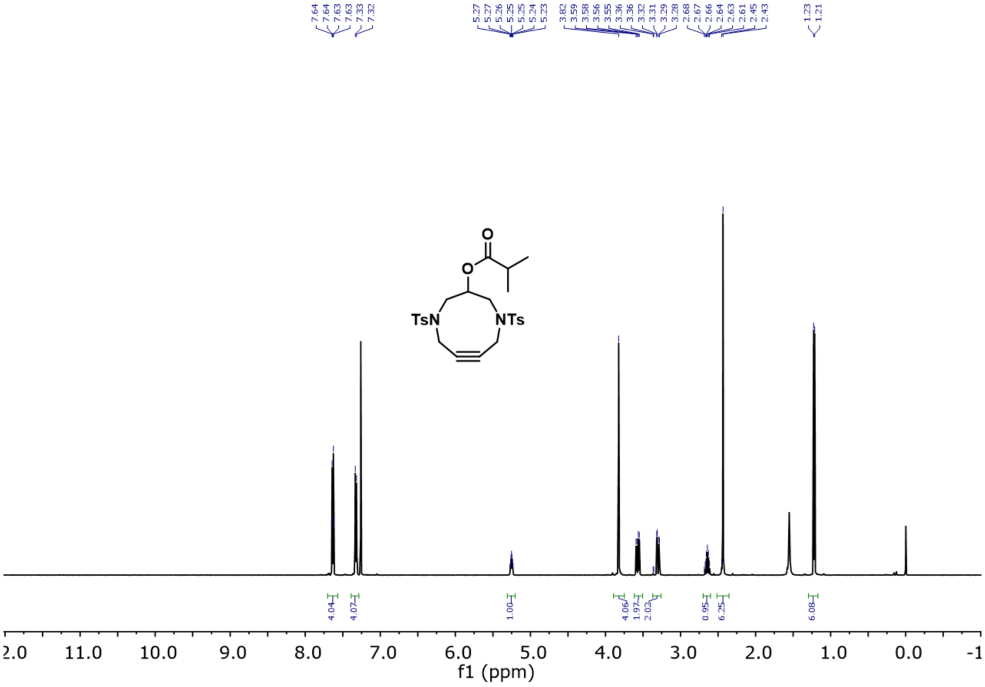

^{13}C NMR of M4 (126MHz, $CDCl_3$)

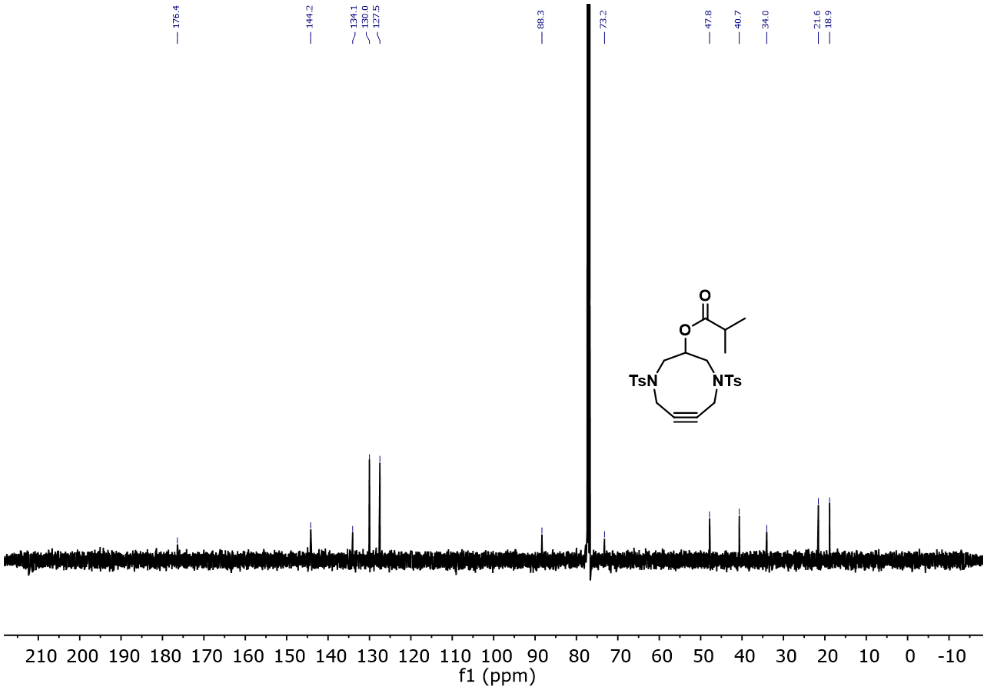

9.2 Polymers NMR Data

^{1}H NMR (600 MHz, $CDCl_3$) of **P1**

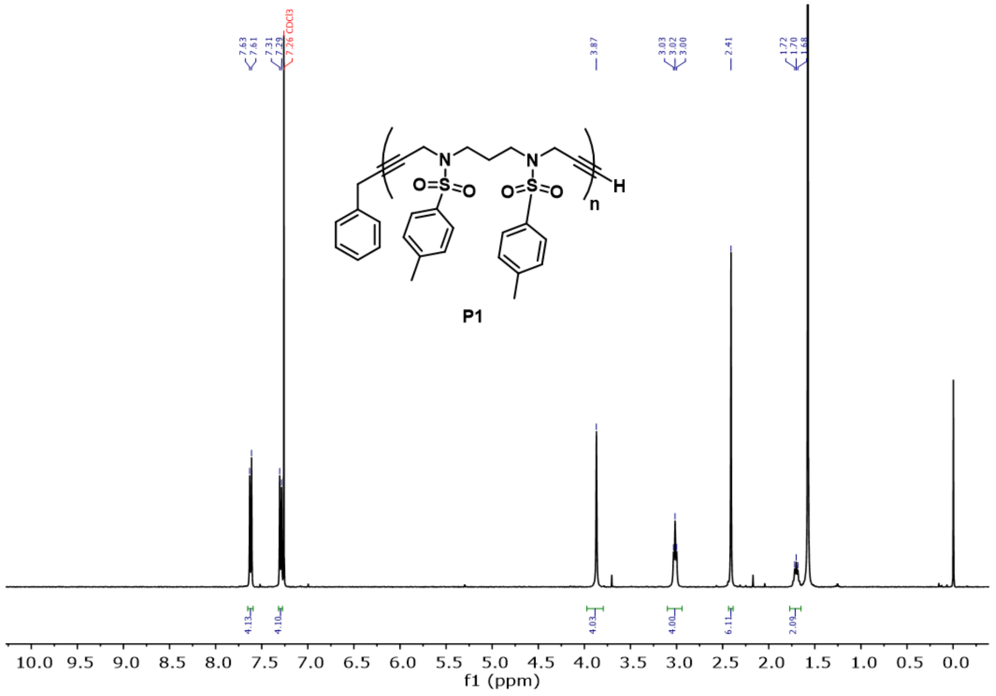

^{13}C NMR (151 MHz, $CDCl_3$) of **P1**

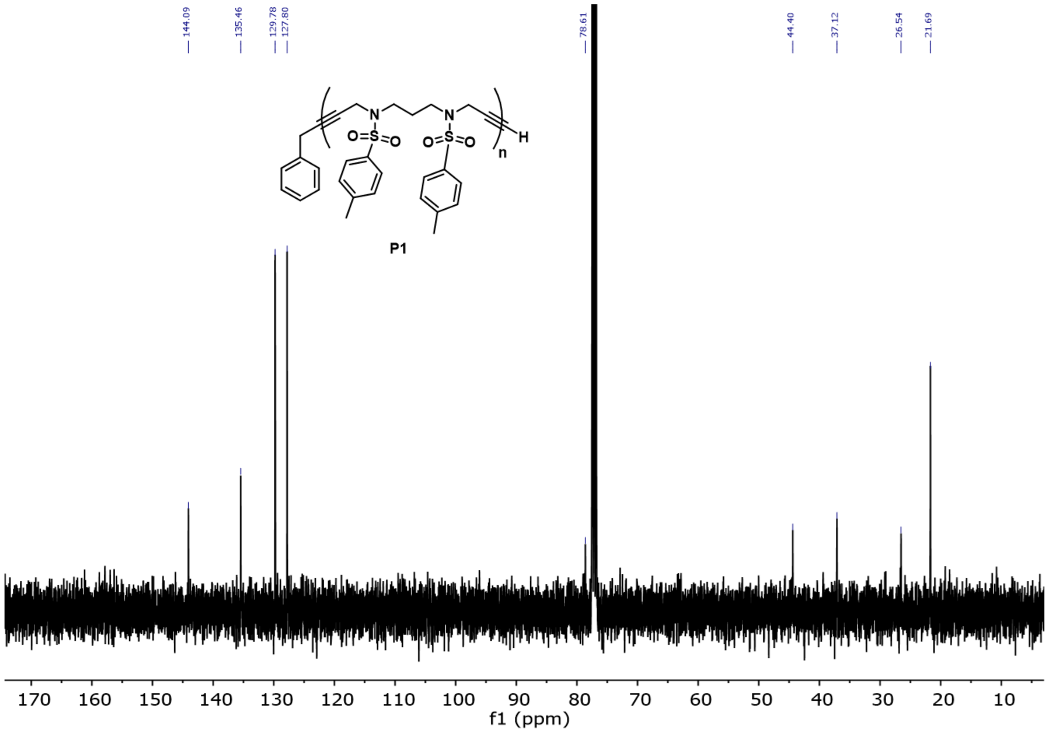

^{1}**H NMR** (600 MHz, $CDCl_3$) of **P2**

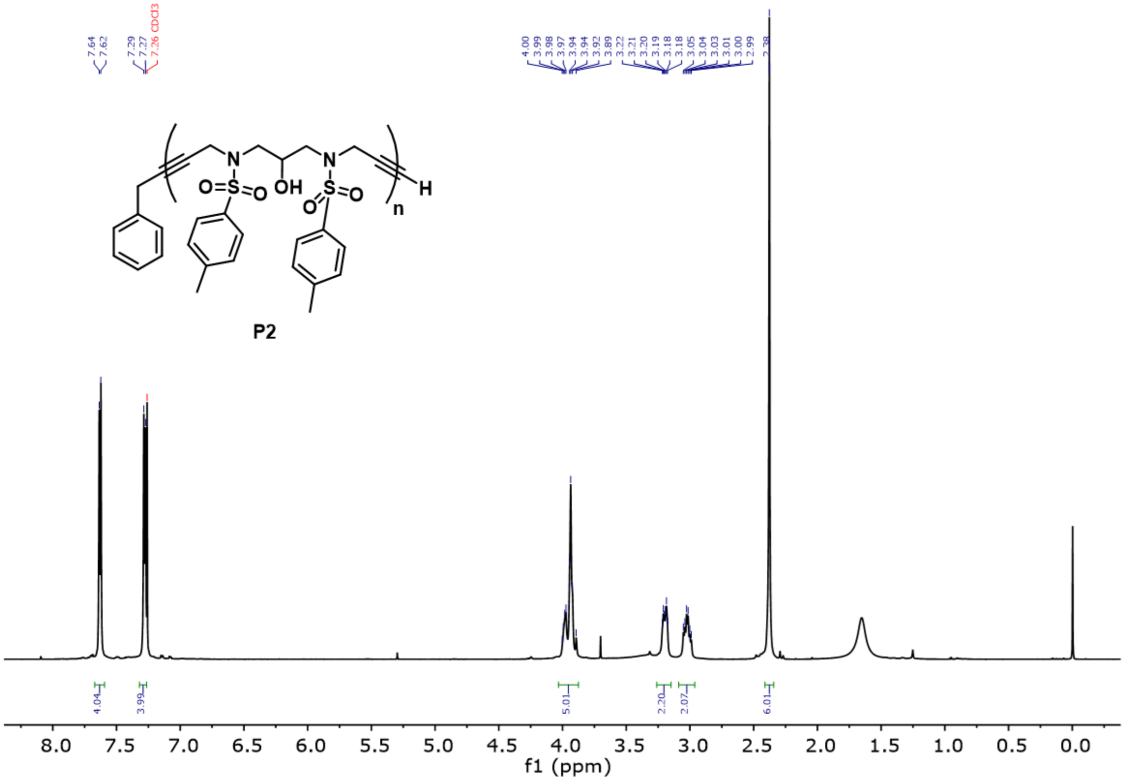

^{13}**C NMR** (151 MHz, $CDCl_3$) of **P2**

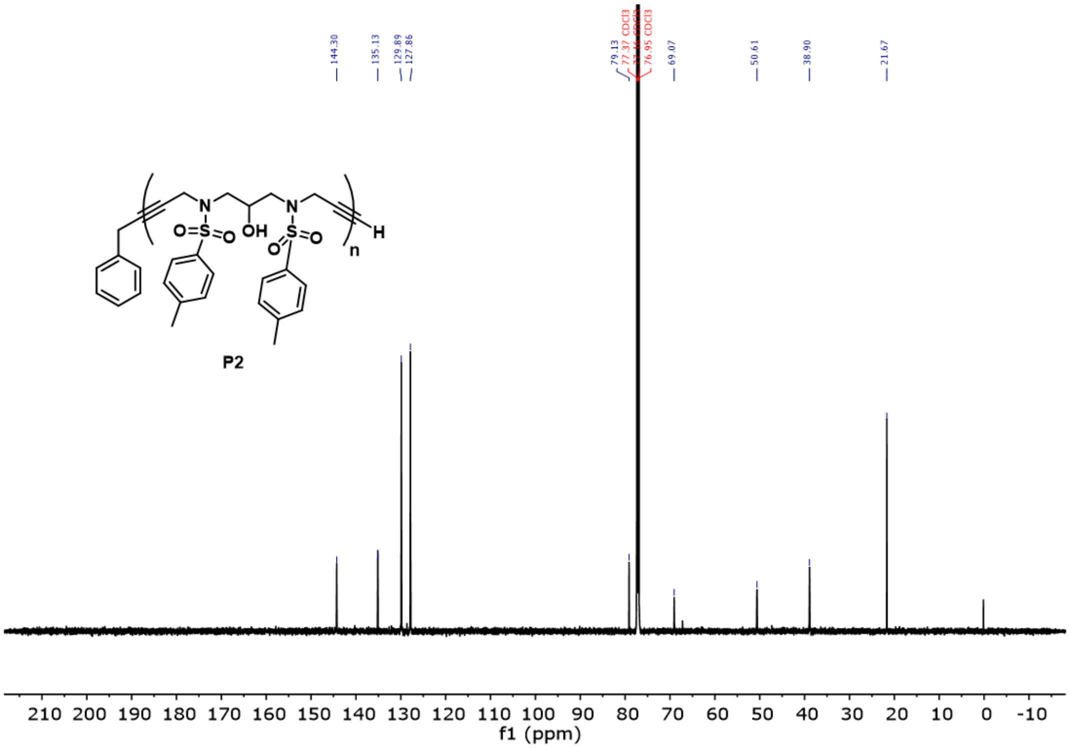

^{1}**H NMR** (600 MHz, $CDCl_3$) of **P3**

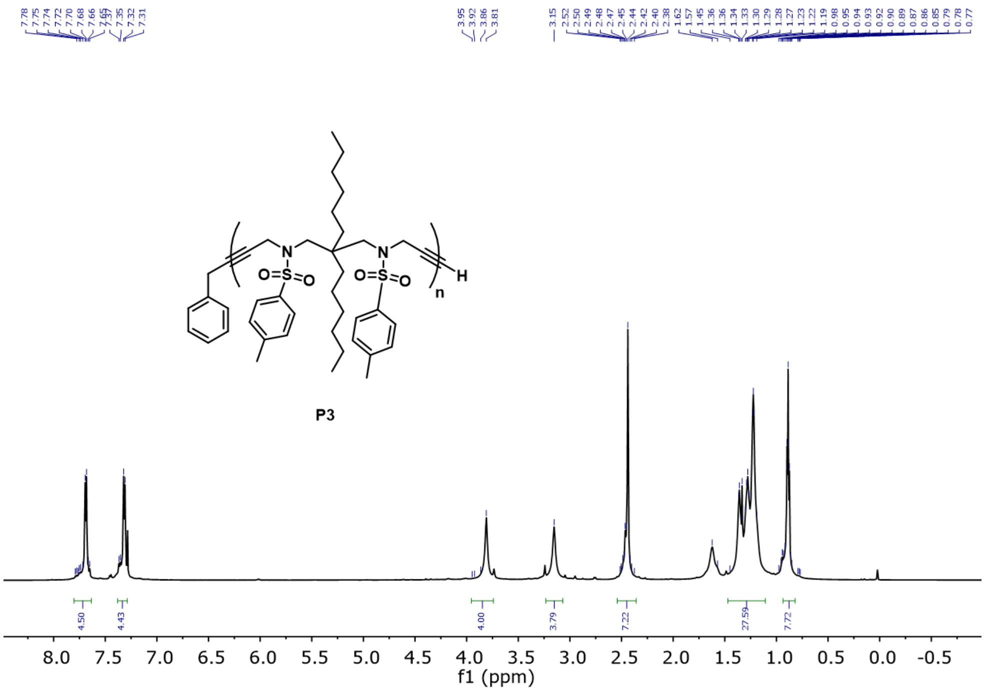

^{13}**C NMR** (151 MHz, $CDCl_3$) of **P3**

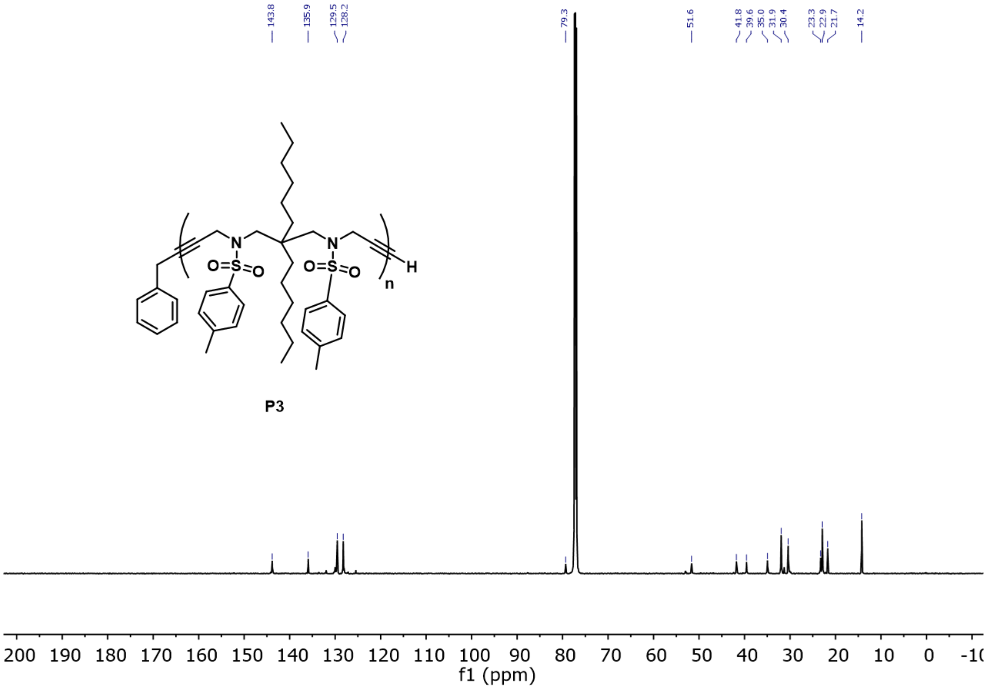

¹H NMR (600 MHz, $CDCl_3$) of **P4**

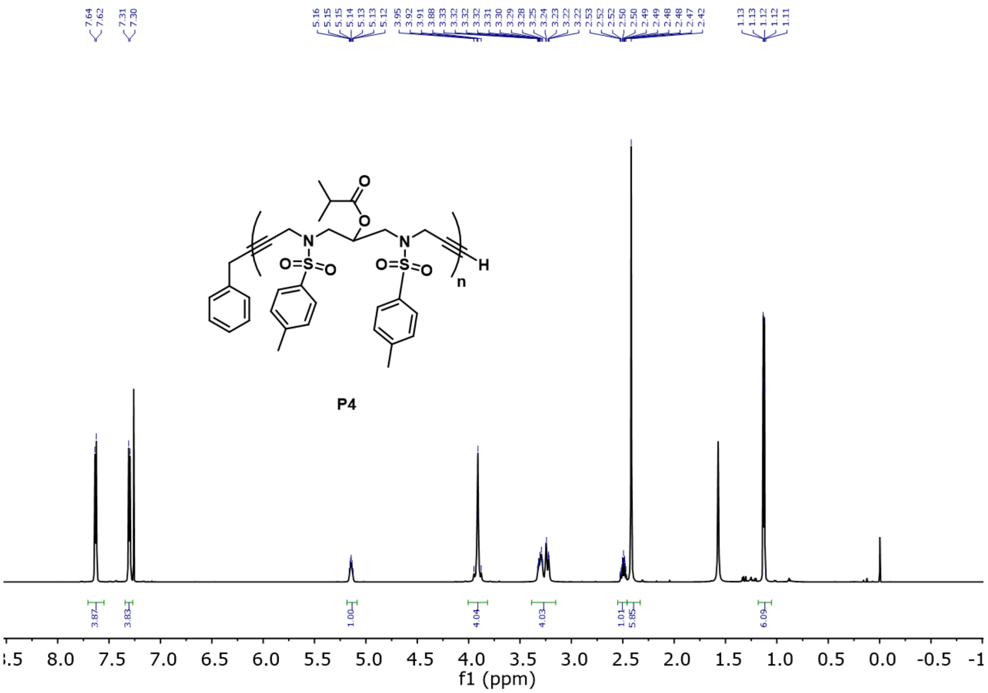

¹³C NMR (151 MHz, $CDCl_3$) of **P4**

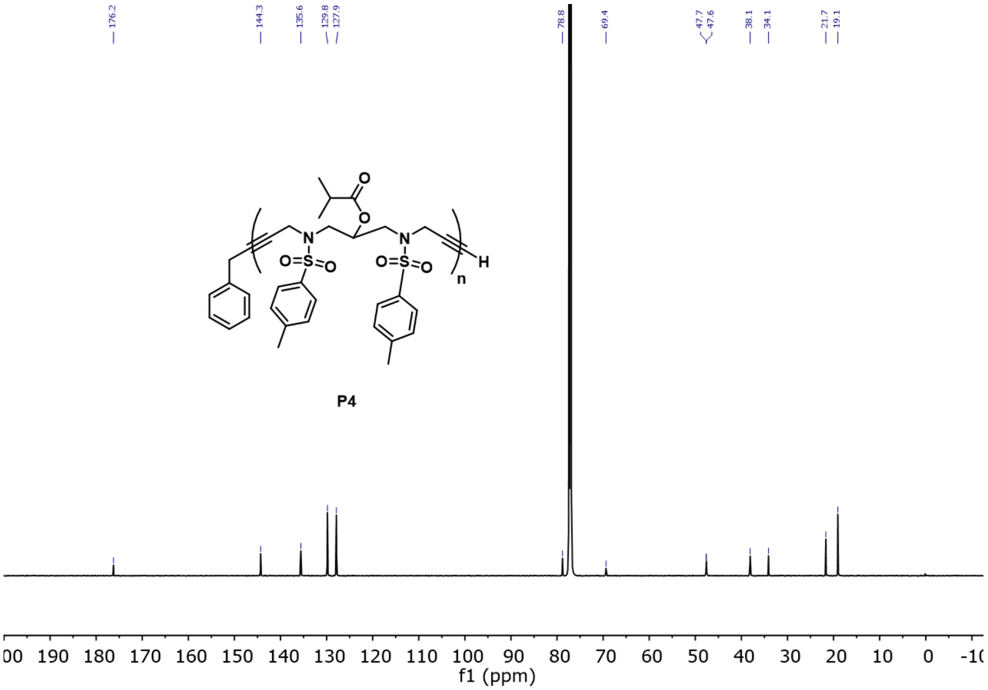

^{1}**H NMR** (600 MHz, $CDCl_3$) of **P2-*b*-P1**

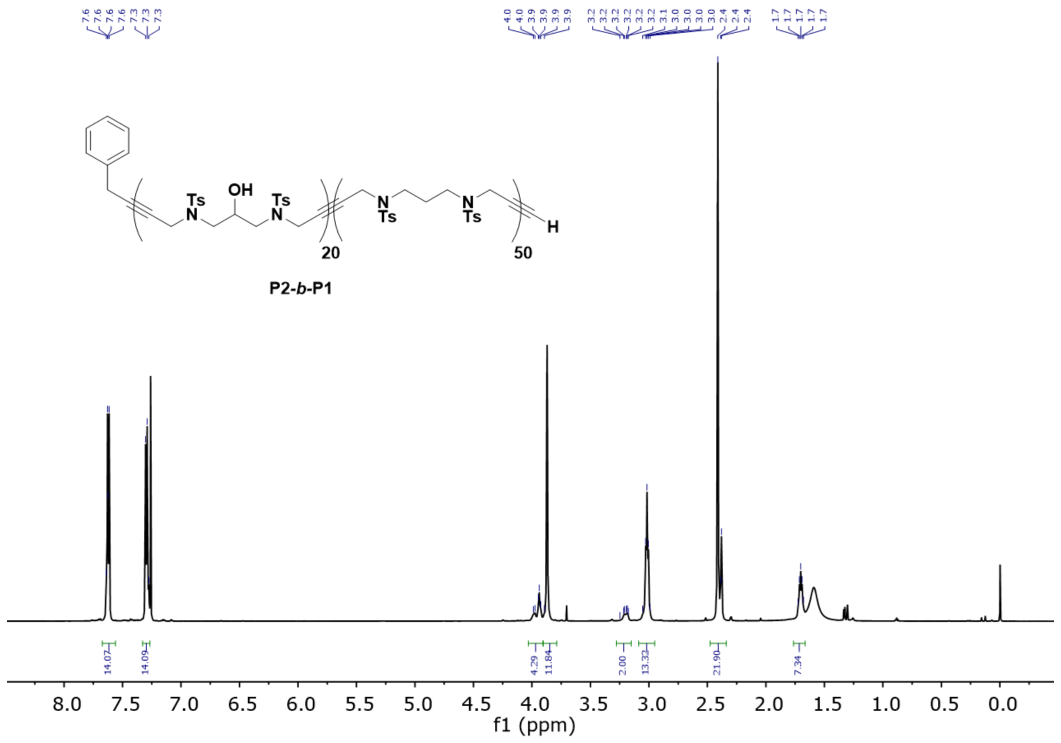

^{13}**C NMR** (151 MHz, $CDCl_3$) of **P2-*b*-P1**

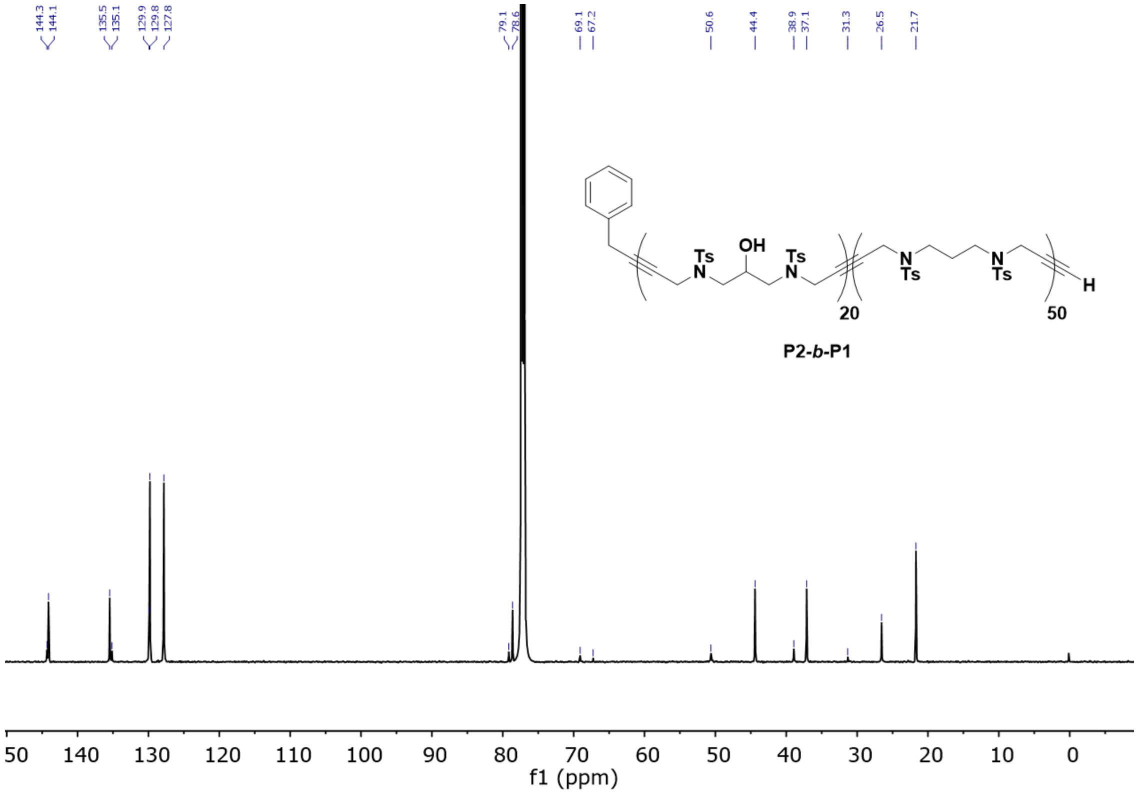

^{1}H NMR (600 MHz, $CDCl_3$) of **P1-*b*-P4**

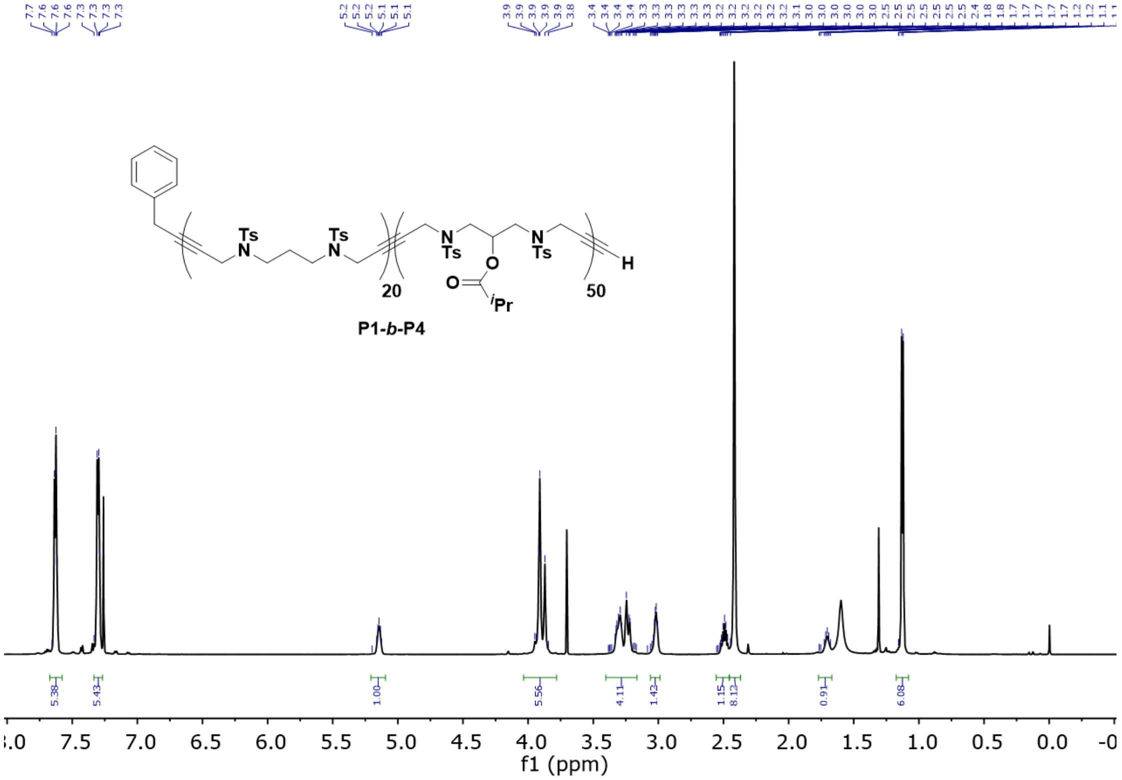

^{13}C NMR (151 MHz, $CDCl_3$) of **P1-*b*-P4**

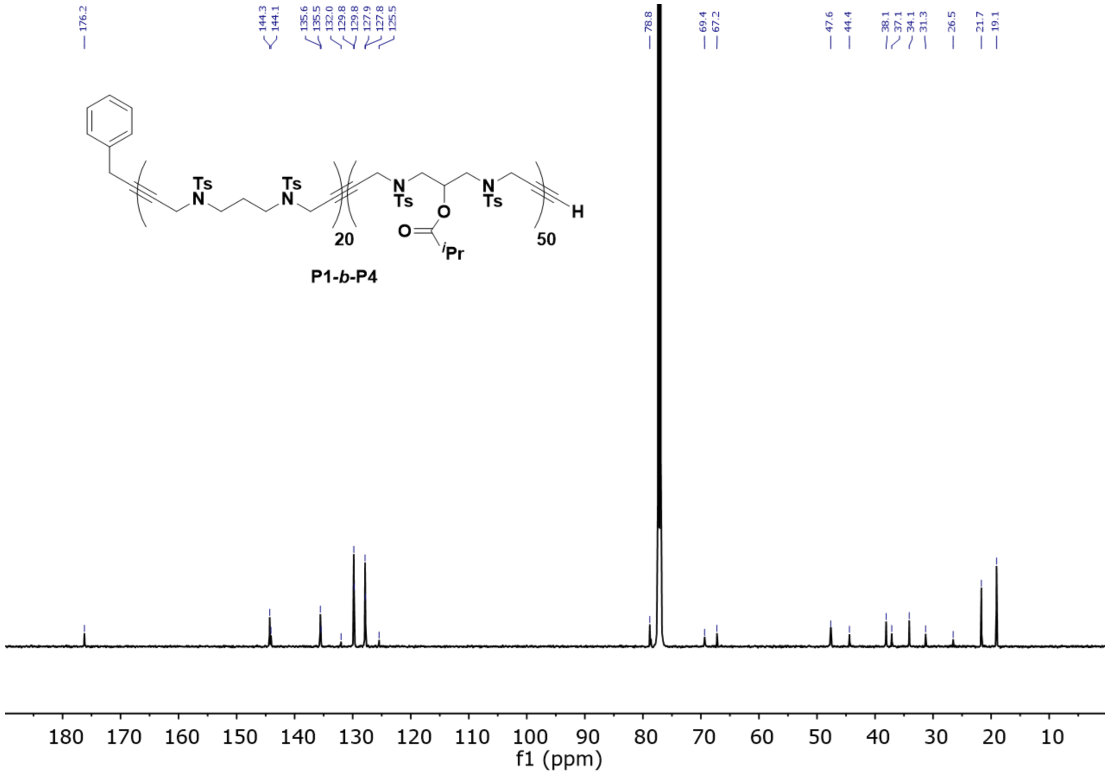

10. References

[1] Ni, R.; Mitsuda, N.; Kashiwagi, T.; Igawa, K.; Tomooka, K. Heteroatom-Embedded Medium-Sized Cycloalkynes: Concise Synthesis, Structural Analysis, and Reactions. *Angewandte Chemie International Edition* **2014**, *54* (4), 1190–1194.

[2] Igawa, K.; Tomooka, K.; Aoyama, S.; Kawasaki, Y.; Kashiwagi, T.; Seto, Y.; Ni, R.; Mitsuda, N. Thieme Chemistry Journals Awardees: Where Are They Now? One-Pot Synthesis of Versatile Buckle Units for Click Chemistry: 4,8-Diazacyclononynes (DACNs). *Synlett* **2017**, *28* (16), 2110–2114.

[3] Kondoh, A.; Terada, M. Nicholas Reaction. *Patai's Chemistry of Functional Groups* **2023**, 1–36.

[4] Cui, M.; Huang, J.; Tsang, L. Y.; Sung, H. H. Y.; Williams, I. D.; Jia, G. Exploring Efficient and Air-Stable D^2 Re(v) Alkylidyne Catalysts: Toward Room Temperature Alkyne Metathesis. *Chemical Science* **2024**, 15, 18318–18326.

[5] Taschner, I. S.; Walker, T. L.; DeHaan, H. S.; Schrage, B. R.; Ziegler, C. J.; Taschner, M. J. Synthesis, Characterization, and Copper(II) Chelates of 1,11-Dithia-4,8-Diazacyclotetradecane. *The Journal of Organic Chemistry* **2019**, *84* (17), 11091–11102.

[6] Danilkina, N. A.; Bukhtiiarova, N. S.; Govdi, A. I.; Vasileva, A. A.; Rumyantsev, A. M.; Volkov, A. A.; Sharaev, N. I.; Povolotskiy, A. V.; Boyarskaya, I. A.; Kornyakov, I. V.; Tokareva, P. V.; Balova, I. A. Synthesis and Properties of 6-Aryl-4-Azidocinnolines and 6-Aryl-4-(1,2,3-1H-Triazol-1-Yl)Cinnolines. *Molecules* **2019**, *24* (13), 2386–2386.

[7] Sugihara, T.; Ban, H.; Yamaguchi, M. Novel Decomplexation Method for Alkyne–Co2(CO)6 Complexes. *Journal of Organometallic Chemistry* **1999**, *554* (2), 163–166.

[8] a) Kaneda, K.; Naruse, R.; Yamamoto, S.; Teppei Satoh. Reactivity of the Sultam and Strained Alkyne Groups in 2-Aminobenzenesulfonamide-Containing Cyclononyne (ABSACN). *Asian Journal of Organic Chemistry* **2018**, *7* (4), 793–801; b) Kaneda, K.; Naruse, R.; Yamamoto, S. 2-Aminobenzenesulfonamide-Containing Cyclononyne as Adjustable Click Reagent for Strain-Promoted Azide–Alkyne Cycloaddition. *Organic Letters* **2017**, *19* (5), 1096–1099.

[9] Fukudome, Y.; Naito, H.; Hata, T.; Urabe, H. Copper-Catalyzed 1,2-Double Amination of 1-Halo-1-Alkynes. Concise Synthesis of Protected Tetrahydropyrazines and Related Heterocyclic Compounds. *Journal of the American Chemical Society* **2008**, *130* (6), 1820–1821.

[10] Wen, J.; Dong, B.; Zhu, J.; Zhao, Y.; Shi, Z. Revealing Silylation of $C(Sp^2)/C(Sp^3)$–H Bonds in Arylphosphines by Ruthenium Catalysis. *Angewandte Chemie International Edition* **2020**, *59* (27), 10909–10912.

[11] Hu, Y.; Wei, Z.; Frey, A.; Kubis, C.; Ren, C.; Anke Spannenberg; Jiao, H.; Werner, T. Catalytic, Kinetic, and Mechanistic Insights into the Fixation of CO_2 with Epoxides Catalyzed by Phenol-Functionalized Phosphonium Salts. *ChemSusChem* **2020**, *14* (1), 363–372.

[12] Sugahara, T.; Espinosa Ferao, A.; Rey Planells, A.; Guo, J.-D.; Aoyama, S.; Igawa, K.; Tomooka, K.; Sasamori, T.; Hashizume, D.; Nagase, S.; Tokitoh, N. 1,2-Insertion Reactions of Alkynes into Ge–c Bonds of Arylbromogermylene. *Dalton Transactions* **2020**, *49* (21), 7189–7196.
